# The phylogeny and evolutionary history of tyrannosauroid dinosaurs

**DOI:** 10.1038/srep20252

**Published:** 2016-02-02

**Authors:** Stephen L. Brusatte, Thomas D. Carr

**Affiliations:** 1 School of GeoSciences, University of Edinburgh, Grant Institute, James Hutton Road, Edinburgh, EH9 3FE, UK; 2 Department of Biology, Carthage College, 2001 Alford Park Drive, Kenosha, WI 53140, USA

## Abstract

Tyrannosauroids—the group of carnivores including *Tyrannosaurs rex*—are some of the most familiar dinosaurs of all. A surge of recent discoveries has helped clarify some aspects of their evolution, but competing phylogenetic hypotheses raise questions about their relationships, biogeography, and fossil record quality. We present a new phylogenetic dataset, which merges published datasets and incorporates recently discovered taxa. We analyze it with parsimony and, for the first time for a tyrannosauroid dataset, Bayesian techniques. The parsimony and Bayesian results are highly congruent, and provide a framework for interpreting the biogeography and evolutionary history of tyrannosauroids. Our phylogenies illustrate that the body plan of the colossal species evolved piecemeal, imply no clear division between northern and southern species in western North America as had been argued, and suggest that *T. rex* may have been an Asian migrant to North America. Over-reliance on cranial shape characters may explain why published parsimony studies have diverged and filling three major gaps in the fossil record holds the most promise for future work.

The 13-meter long, 7-tonne, bone-crunching *Tyrannosaurus rex* is a fossil icon^1^,^2^,^3^. This dinosaur reigned at the top of the food chain in North America at the end of the Cretaceous (~66–67 million years ago), and was among the last survivors of a major group of carnivorous theropods—the Tyrannosauroidea—that originated more than 100 million years earlier^3^,^4^,^5^. The origins, phylogeny, and evolution of tyrannosauroids were long mysterious. However, recent discoveries reveal that these dinosaurs were once widely distributed and were mostly small and ecologically marginal until some species developed enormous size during the final 20 million years of the Age of Dinosaurs, before the end-Cretaceous asteroid struck them down in their prime^3^,^6^,^7^,^8^,^9^,^10^.

The pace of research on tyrannosauroids has recently been frenetic, with more than half of their known diversity discovered over the past decade alone. In 2010 we published a comprehensive phylogenetic analysis of tyrannosauroids^3^, but since that time many new species have been found, which have generated new questions about the biogeography of the group, the quality of their fossil record, and the origin of the characteristic deep-skulled, short-armed body plan of the colossal derived tyrannosaurids (*T. rex* and closest kin)^11^,^12^,^13^,^14^,^15^. These mysteries have deepened with the recent publication of an alternative phylogenetic analysis, which recovers strikingly different relationships from our earlier study^13^.

These developments prompted us to revisit the phylogenetic relationships of Tyrannosauroidea. We present a new analysis, building on our earlier study^3^, which includes all newly discovered species and incorporates data from the alternative analysis of Loewen *et al*.^13^, providing an up-to-date and comprehensive dataset for exploring the genealogy, biogeography, and evolutionary history of these most familiar of dinosaurs.

All previous studies of tyrannosauroid phylogeny have used parsimony^3^,^4^,^5^,^6^,^7^,^8^,^9^,^12^,^13^,^14^,^15^,^16^,^17^,^18^,^19^,^20^. We followed these studies—and the vast majority of phylogenetic studies of fossil organisms using morphological data—in analyzing our new dataset with parsimony. However, we also ran a second series of analyses in which we applied Bayesian techniques to our dataset. This is the first time such techniques have been used to study tyrannosauroid phylogeny. Bayesian methods have become standard in molecular phylogenetic studies and are becoming more widely used in morphological studies^21^,^22^,^23^,^24^,^25^,^26^,^27^. They offer greater flexibility in employing different models of evolution, including more complex models in which characters can evolve at different rates, which can be tested against each other to statistically assess which best fits the data. They also have the attractive property of not being susceptible to long-branch attraction, which can be a common problem in morphological datasets with large amounts of homoplasy. In an operational sense, our Bayesian results also provide a novel viewpoint on tyrannosauroid phylogeny and evolution. In the same way that multiple statistical tests are often used to bolster confidence in a result, we look for congruence between parsimony and Bayesian trees to identify areas of tyrannosauroid phylogeny that are more robustly supported than others.

## Results

**Figure 1:**
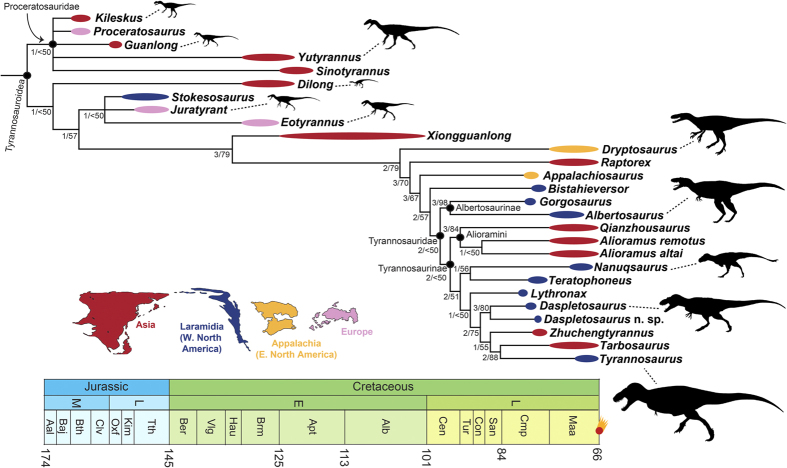
The phylogenetic relationships of Tyrannosauroidea, based on parsimony analysis. Strict consensus topology of five most parsimonious trees recovered from the cladistic analysis. Numbers by nodes indicate Bremer and jackknife support values. Thick lines next to each taxon depict temporal range, which in most cases is age uncertainty and not true range, and colors of lines denote geographic areas. Branches of the phylogeny are not scaled to time. Silhouettes are in relative proportion and scaled to total body length (*T. rex* = 13 meters). Geographic silhouettes from Loewen *et al*.^13^ and taxon silhouettes from phylopic.org (*Kileskus*: T.M. Keesey; *Guanlong*: S. Hartman; *Yutyrannus*: S. Hartman; *Dilong*: FunkMonk; *Juratyrant*: S. Hartman, T.M. Keesey; *Eotyrannus*: S. Hartman; *Dryptosaurus* T.M. Keesey; *Albertosaurus* C. Dylke; *Nanuqsaurus* J. Headden; *Daspletosaurus* S. O’Connor, T.M. Keesey; *Tyrannosaurus* S. Hartman).

The parsimony analysis recovered five most parsimonious trees (765 steps, consistency index = 0.557, retention index = 0.812). The strict consensus topology (Fig. 1) shows that Tyrannosauroidea comprises three major clusters: a basal Proceratosauridae clade of mostly small-bodied species with elaborate cranial crests from the Middle Jurassic–Early Cretaceous (such as *Guanlong* and *Proceratosaurus*), an intermediate grade of mostly small-to-mid sized taxa that lived during the Late Jurassic–Early Cretaceous (such as *Dilong* and *Eotyrannus*), and a derived clade of large-bodied, latest Cretaceous apex predators that includes Tyrannosauridae and their very closest relatives (e.g., *Albertosaurus, Bistahieversor, Tyrannosaurus*). Relationships among the derived large-bodied taxa are mostly robustly supported by jackknife and Bremer values, but support for basal tyrannosauroid interrelationships is much weaker.

Compared to previous analyses based on parsimony, these results generally follow our 2010 analysis^3^, but differ in some ways from the topology of Loewen *et al*.^13^. In our analysis, *Juratyrant* and *Stokesosaurus* form a clade with the ‘intermediate’ taxon *Eotyrannus*; *Bistahieversor* is positioned just outside Tyrannosauridae; the long-snouted alioramins are nested within Tyrannosauridae as basal tyrannosaurines; both species of *Daspletosaurus* form a clade of derived tyrannosaurines closely related to *Tyrannosaurus*; and *Tyrannosaurus* and *Tarbosaurus* are sister taxa. In Loewen *et al*.’s study, *Juratyrant* and *Stokesosaurus* are proceratosaurids; *Bistahieversor* is a derived tyrannosaurid nested deep within Tyrannosaurinae; the alioramins are basal taxa outside of Tyrannosauridae; the species of *Daspletosaurus* are a paraphyletic grade of very basal tyrannosaurines; and *Tarbosaurus* and *Zhuchengtyrannus* are sister taxa exclusive of *Tyrannosaurus*.

**Figure 2:**
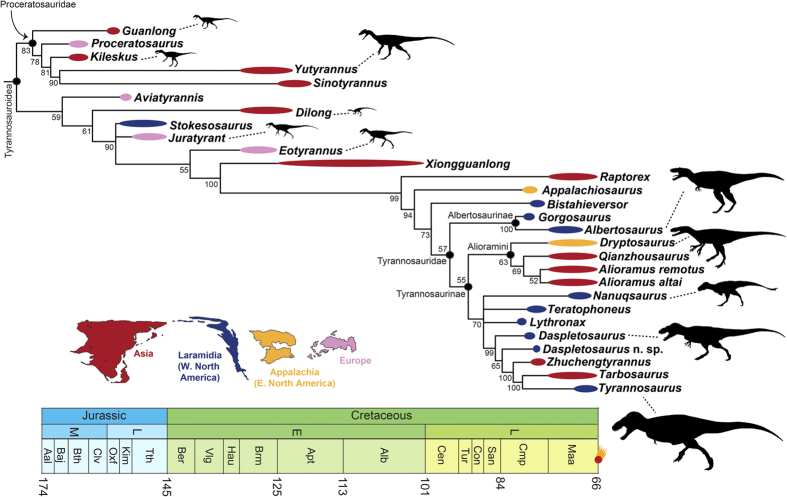
The phylogenetic relationships of Tyrannosauroidea, based on Bayesian analysis. Bayesian consensus topology recovered from the cladistic analysis. Numbers by nodes indicate posterior probabilities of each clade. All other conventions as in Fig. 1.

Our Bayesian consensus topology (Fig. 2) is strikingly similar to our parsimony results. It also divides Tyrannosauroidea into three main clusters: the basal Proceratosauridae, an intermediate grade, and a latest Cretaceous clade of derived, large-bodied species. As with our parsimony results, *Bistahieversor* is recovered outside of Tyrannosauridae and the alioramins are placed as basal tyrannosaurines, contrasting with the parsimony results of Loewen *et al*.^13^. The main differences with our parsimony results are minor: Proceratosauridae is fully resolved in the Bayesian tree (whereas all proceratosaurids form a polytomy in the parsimony tree), *Eotyrannus* is recovered as more closely related to tyrannosaurids than to *Stokesosaurus* and *Juratyrant* (whereas the three form a clade in the parsimony tree), *Dryptosaurus* is placed as a basal tyrannosaurine in a clade with the alioramins (not as a non-tyrannosaurid as in the parsimony analysis), *Daspletosaurus* is paraphyletic, and Tyrannosaurinae is more poorly resolved (particularly the positions of *Teratophoneus, Lythronax*, and *Nanuqsaurus*). Our Bayesian analysis also resolves the position of *Aviatyrannis*, placing it at the base of the clade that includes all non-proceratosaurid tyrannosauroids, whereas this poorly known taxon was a wildcard in the parsimony analysis (i.e., it occupies many different and widely varying positions in the individual most parsimonious trees, so tends to collapse the resolution of many clades in the strict consensus tree).

## Discussion

### Differences between parsimony and Bayesian topologies

In comparing our parsimony and Bayesian phylogenies, the most striking finding is that the two methods produce extremely similar consensus trees. The overall structure of both trees is identical: a basal clade of proceratosaurids, an intermediate grade of small-to-mid-sized tyrannosauroids, and a derived clade of very large apex predators. Most of the small details are identical as well: the fairly large *Sinotyrannus* and *Yutyrannus* group with proceratosaurids instead of the large-bodied tyrannosaurids; *Dilong, Eotyrannus*, and *Xiongguanlong* are successively closer outgroups to Tyrannosauridae; *Bistahieversor* and *Appalachiosaurus* are non-tyrannosaurids; tyrannosaurids are divided into Albertosaurinae and Tyrannosaurinae subclades; and the long-snouted alioramins are basal tyrannosaurines. These results are encouraging, as they show that the major outline of tyrannosauroid phylogeny is recovered by multiple methods that differ substantially in their starting assumptions, at least when these methods are applied to the same dataset.

There are some differences between our parsimony and Bayesian topologies, however, and these deserve further discussion. As outlined above, there are three main differences: the position of *Dryptosaurus* (non-tyrannosaurid in the parsimony tree, nesting with the alioramin tyrannosaurines in the Bayesian tree), the status of *Daspletosaurus* (the two species form a monophyletic cluster in the parsimony analysis but are a grade on the line to more derived tyrannosaurines in the Bayesian analysis), and resolution within Tyrannosaurinae (completely resolved in the parsimony analysis, one main polytomy including *Lythronax* and *Teratophoneus* in the Bayesian analysis). What is particularly interesting is that the basal position of *Dryptosaurus* and the monophyly of *Daspletosaurus* are supported by relatively high Bremer and jackknife values in the parsimony tree, but their alternative placements are supported by relatively high posterior probabilities in the Bayesian tree.

Understanding exactly why parsimony and Bayesian methods produce different results in this case is difficult, as the differences have to do with relatively minor aspects of the topology. However, we note that the most salient differences have to do with the relationships of the oldest and most basal tyrannosaurines. We hypothesize that the conflict reflects, in part, the ~20 million year gap between derived tyrannosaurids and their common ancestor that preceded the transgression of the Western Interior Seaway in North America. This gap reflects a large amount of missing data, in the form of tyrannosauroid taxa that must have been present but are not currently sampled (see further discussion of fossil record biases below). Perhaps the conflicting parts of the phylogeny correspond to clades of closely related species that have long ghost lineages with many missing taxa (e.g., Alioramini, basal Tyrannosaurinae). For example, Tyrannosaurinae almost certainly had a lengthy but unsampled early history in Asia and western North America. There is also potentially a long ghost lineage leading to *Dryptosaurus*, one of the few tyrannosauroids known from the latest Cretaceous of eastern North America^28^. Our hypothesis that ghost ranges and biased sampling may be causing conflict among parsimony and Bayesian results can be tested in the future, both as new discoveries fill these poorly sampled portions of tyrannosaur history and with Bayesian evolutionary models that can better take into account uneven sampling.

### Rectifying differences in tyrannosauroid phylogenetic studies

One of the most vexing roadblocks in understanding tyrannosauroid evolution has been the discrepancy between our original 2010 phylogeny^3^ and the alternative study of Loewen *et al*.^13^, as these genealogies imply different biogeographic and evolutionary histories. Our integration of these two datasets is an attempt to bridge the gap and produce a novel phylogeny that can be used to study tyrannosauroid evolution.

After assimilating the two datasets we recover a phylogeny that is much more similar to our 2010 cladogram, and considerably different from Loewen *et al*.’s topology. This is true of both our parsimony and Bayesian results. This is likely because we discarded several of Loewen *et al*.’s characters that we considered problematic. We argue that many of these are redundant with each other because they relate to overall skull proportions (see supplementary information). An over-abundance of these characters may explain why Loewen *et al*.^13^ found the long-snouted alioramins to nest outside of the deep-snouted tyrannosaurids, and the deep-skulled *Bistahieversor* to group with derived tyrannosaurines that have a similar skull shape, whereas our phylogeny recovers different results in which major clades are not so cleanly diagnosed by similar skull proportions. If our topology is correct, Loewen *et al*.’s findings may result from something analogous to the long-noted ‘longirostrine problem’, which causes crocodylomorphs with similar skulls shapes to artefactually group together in phylogenetic analyses when in fact they are distantly related^29^,^30^.

Given that parsimony and Bayesian analyses of our dataset return similar results, but that our new phylogeny has key differences with the Loewen *et al*.^13^ study, it appears as if data selection (character choice and scorings) are more important drivers of topological differences than is methodology. Aside from the discovery of new specimens, we suggest that future authors pay close attention to character selection and scoring, present detailed rationale for why they have excluded certain published characters or changed scores, and ideally work together to come to a joint understanding of the primary data that is input into TNT, Mr Bayes, and other software to be used for phylogenetic analysis.

### Consensus and conflict in tyrannosauroid phylogeny

Although there are some differences between our trees and the phylogeny of Loewen *et al*.^13^, and some differences between the parsimony and Bayesian analyses of our new dataset, we are generally optimistic about the state of consensus in tyrannosauroid phylogenetics. All of these varying analyses, using different character datasets and now different methods, agree on the broad framework of tyrannosauroid genealogy: there is a basal cluster of proceratosaurids, an intermediate grade of species like *Dilong* and *Eotyrannus*, and a derived group of the largest tyrannosauroids from the latest Cretaceous. This clade of large, derived species is divided into clusters centered on *Albertosaurus* and *Gorgosaurus* (Albertosaurinae) and *Tyrannosaurus* and *Daspletosaurus* (Tyrannosaurinae). The largest Early Cretaceous tyrannosauroids like *Sinotyrannus*^31^ are primitive proceratosaurids, not close relatives of *Tyrannosaurus* or *Albertosaurus*. Seeing as these results are now commonly found in recent phylogenetic analyses, we consider them to be a well-supported hypothesis.

### Tyrannosauroid body plan evolution

Our new phylogeny illustrates that the characteristic body plan of the colossal latest Cretaceous tyrannosaurids did not develop rapidly, but in a more piecemeal fashion. There is a general increase in body size across tyrannosauroid phylogeny (Figs 1 and 2), features that enabled ever-stronger bite forces evolved incrementally, and cranial ornamentation gradually became more elaborate on the line to the very largest tyrannosauroids like *T. rex* (see expanded discussion in supplementary information).

The basal tyrannosauroids *Yutyrannus* and *Sinotyrannus* are early examples of fairly large body size in the group (~8–9 meters long and 1.5 tons in mass^12^,^31^), but these taxa are not closely related to the derived latest Cretaceous tyrannosaurids, as they are part of the early-flourishing proceratosaurid radiation and not on the direct line to *T. rex* and close relatives. Their body plan is also quite different from the characteristic colossal bauplan of the latest Cretaceous apex tyrannosaurids. The skulls of *Yutyrannus* and *Sinotyrannus* are shallower and less robust than *T. rex* and kin, with smaller jaw muscles and thinner teeth, and, at least in *Yutyrannus*, an elaborate midline cranial crest rather than the prescribed series of circum-orbital cranial ornaments in tyrannosaurids (although some of these ornamental features are present in *Yutyrannus*, their size, shape, and position drastically differ from taxa like *T. rex* and *Albertosaurus*). The postcranial skeletons are also quite different, as most notably *Yutyrannus* has a large and three-fingered arm that does not resemble the withered two-fingered forearm of *T. rex* and close relatives. With its gaudy midline skull crest, huge external naris, and proportionally long arms, *Yutyrannus* (and probably *Sinotyrannus*) resemble overgrown versions of *Guanlong*, not proto-tyrannosaurids.

The peculiar early Early Cretaceous *Yutyrannus* and *Sinotyrannus* indicate that tyrannosauroids were capable of evolving moderately large sizes during the middle portion of their evolutionary history. But colossal size (>10 metres in body length, >1.5 tons in mass) came much later, first appearing in the Campanian, ca. 80 million years ago, although this observation is probably clouded by sampling biases (see below). Many of the features so characteristic of the colossal end-Cretaceous taxa—such as a broad U-shaped snout, a ventrally convex maxilla, and asymmetrical carinae on the maxillary teeth—make their first appearance in the late Early Cretaceous *Xiongguanlong*^32^, which we consider as the earliest taxon to exhibit something of a ‘tyrannosaurid-grade bauplan’ (see supplementary information). With that said, *Xiongguanlong* does not show any clear signs of developing gigantism, as its body mass is estimated at a modest 170 kg, an order of magnitude lower than tyrannosaurids^33^.

### Tyrannosauroid biogeography

Our new phylogeny helps elucidate the biogeographic history of tyrannosauroids, and implies a much different narrative than the topology of Loewen *et al*.^13^. They recover a series of tyrannosaurids (albertosaurines and basal tyrannosaurines) from northern Laramidia (western North America) on the line to a derived subclade of southern Laramidian taxa, which they interpret as support for a major biogeographic division between northern and southern faunas during the latest Cretaceous^13^,^34^. Both our parsimony and Bayesian phylogenies find no clear division between northern and southern species, which are interspersed with each other and with Asian taxa (Figs 1 and 2). In some cases we find close relationships between taxa that are widely separated latitudinally, such as *Nanuqsaurus* (Alaska) and *Teratophoneus* (Utah) in our parsimony analysis. Rather than depicting tyrannosaurids as provincial animals with a highly ordered geographic distribution, our topology suggests that they were dynamic organisms capable of recurrent faunal interchange.

The derived large-bodied tyrannosaurids inhabited both Asia and North America during the final ~20 million years of the Cretaceous. Loewen *et al*.^13^ argued for a single dispersal between western North America, which they considered the point of origin for Tyrannosauridae, and Asia when global sea levels fell during the late Campanian. Our phylogeny implies more frequent interchange that is not so clearly linked to sea level changes. The placement of the Asian alioramins as basal tyrannosaurines indicates at least one other dispersal episode, prior to the middle Campanian (ca. 80 million years ago, based on the age of the more highly nested *Lythronax*). Additionally, we find *Tyrannosaurus* nested within a subclade of tyrannosaurines that otherwise includes two Asian taxa (*Tarbosaurus* and *Zhuchengtyrannus*). It is equally parsimonious that the two Asian lineages dispersed from North America independently or that this subgroup originated in Asia and *Tyrannosaurus* then immigrated to North America. In other words, it may be that *T. rex* was an invasive migrant species that spread across Laramidia. This may help explain why the latest Maastrichtian North American record is so unusual in yielding only a single large tyrannosaurid species, whereas sympatry of multiple tyrannosaurids is seen in the late Campanian of Alberta and Montana and the Maastrichtian of Asia.

Ultimately, these competing biogeographic scenarios could be tested by more explicit quantitative analysis, such as the likelihood-based techniques employed by Loewen *et al*.^13^. We argue, however, that despite the flurry of recent discoveries the tyrannosauroid fossil record is so incomplete (see below) that the results of these analyses may not be robust, as they can only incorporate known taxa and cannot easily compensate for sampling biases.

### Fossil record biases and future directions

Tyrannosauroids are the subject of more research and popular interest than most, or perhaps all, other dinosaurs. However, their fossil record is frustratingly incomplete and patchy. Three main biases currently hamper our ability to understand long-term biogeographic and evolutionary patterns in tyrannosauroids. First, there is a gap of at least 20 million years, and perhaps up to 45 million years, between the large-bodied latest Cretaceous clade and its sister taxon in which the first whispers of its body plan appear, the late Early Cretaceous *Xiongguanlong*. Filling this gap is critical to determining where Tyrannosauridae originated and how it dispersed in concert with the extreme sea level fluctuations of the middle Cretaceous.

Second, the diversity of large-bodied tyrannosauroids in the latest Cretaceous of Asia is clearly underestimated, as all but one taxon is Maastrichtian in age. There is high Campanian diversity in North America and long ghost lineages that extend to Asian taxa, suggesting that many tyrannosauroids probably lived in the Campanian of Asia as well, but have yet to be sampled. Finding these taxa will be critical in better understanding the number and nature of interchange events between North America and Asia, and how these were affected by sea level change.

Third, we know very little about the tyrannosauroids that lived in Appalachia (eastern North America) during the terminal Cretaceous, as only two species have been recovered from this landmass, despite its large size. These taxa—*Appalachiosaurus* and *Dryptosaurus*—are both somewhat basal, non-tyrannosaurid tyrannosauroids^17^,^28^ (although our Bayesian analysis suggests that *Dryptosaurus* may be more derived, and within Tyrannosauridae). Future discoveries are needed to assess whether tyrannosaurids proper lived in Appalachia, or whether this microcontinent was a refugium for more primitive tyrannosauroids while the super-sized, derived tyrannosaurids were thriving in Laramidia.

We hold that filling these gaps will be the next big step in tyrannosauroid research, as they may help rectify the differences between competing phylogenies of Tyrannosauroidea, make parsimony and Bayesian analyses more congruent with each other, and lead to a breakthrough in our understanding of the biogeographic and evolutionary history of these most famous of dinosaurs.

## Methods

### Character dataset

Several major developments in the field of tyrannosauroid palaeontology have occurred since the publication of our original phylogenetic analysis^3^. A major alternative phylogenetic dataset, compiled by an independent team of researchers, was published by Loewen *et al*.^13^. Five new tyrannosauroids were discovered and named: *Lythronax*^13^, *Nanuqsaurus*^14^, *Qianzhousaurus*^15^, *Yutyrannus*^12^, and *Zhuchengtyrannus*^11^. The British taxon *Stokesosaurus langhami* was removed from the genus *Stokesosaurus*, based on a holotype from North America, and transferred to a new genus, *Juratyrant*^20^. Important new material of *Teratophoneus* was described^13^, greatly expanding the available morphological data for this taxon. It was recognized that a specimen of *Daspletosaurus* does not belong to the type species, *D. torosus*, but is a new taxon^35^. The status of the controversial small-bodied *Raptorex* was reviewed and this taxon was shown to be latest Cretaceous in age^36^, not Early Cretaceous as originally described^37^.

Our new dataset here incorporates all of this new information. This new dataset includes 366 discrete anatomical characters scored for 28 tyrannosauroids and four outgroups. The dataset begins with characters from our original 2010 study^3^ and additional characters added by subsequent authors^15^,^20^. We critically reviewed the characters of Loewen *et al*.^13^, accepting many and incorporating these into our dataset, but discarding those that are already in our dataset or are invariant among tyrannosauroids, and those that we judged as problematic (see the supplementary information). We consider the resulting dataset presented herein to be a merging of the two studies^3^,^13^, although we recognize that other workers may merge the two datasets slightly differently, given the subjectivity that can be associated with the writing and scoring of morphological cladistic characters.

We include all species-level tyrannosauroids that are valid taxa, with a few exceptions. *Coelurus* and *Tanycolagreus* may be basal tyrannosauroids^38^,^39^, but because this has yet to be established convincingly they are excluded here. *Alectrosaurus* is excluded because it is currently under monographic study by one of us (TDC) and will be incorporated into our dataset as part of that project. *Bagaraatan* is likely a chimaera of specimens belonging to tyrannosauroids and other taxa^40^, so we do not include it. Recent work has suggested that megaraptorans may be nested within Tyrannosauroidea^41^ and not among basal tetanurans as found in previous studies^42^,^43^. Because the tyrannosauroid affinities of megaraptorans have yet to be tested in a higher-level theropod analysis that includes a wealth of data pertinent to coelurosaurs and basal tetanurans analyzed simultaneously, we do not include megaraptorans here. However, we are open to the possibility that they may be tyrannosauroids and hope to test this in the future.

We do not include the controversial taxon ‘*Nanotyrannus lancensis*’ in our dataset, because our previous work has argued that this taxon is a nomen dubium, as it is represented by material that belongs to juvenile *Tyrannosaurus rex*^44^,^45^. Several purported diagnostic characters seen in the type specimen of ‘*Nanotyrannus lancensis*’ (Cleveland Museum of Natural History, CMNH 7541) have been shown to reflect the specimen’s juvenile maturity and/or postmortem damage to the skull^44^. Additionally, several other purported diagnostic features are only seen in adult *T. rex* among derived tyrannosaurids, and many other features of the specimen are juvenile conditions well known in other tyrannosaurids, lending further evidence that CMNH 7541 is a juvenile tyrannosaurid referable to *T. rex*^44^. Therefore, we exclude ‘*Nanotyrannus lancensis*’ from our study because the weight of current evidence shows that it is an invalid taxon. We do note, however, that some workers argue that ‘*Nanotyrannus lancensis*’ may be valid^46^. The recent discovery of a nearly complete skeleton of a small-bodied tyrannosaurid from the latest Cretaceous of Montana may hold the key to solving this debate. However, this specimen is currently unavailable for study^47^. We look forward to adding this specimen to our dataset once it becomes available, and suggest that such an exercise will be critical in determining whether ‘*Nanotyrannus lancensis*’ is a valid taxon or a juvenile *T. rex*.

The character list, data matrix, detailed analytical protocols, and scoring sources for each taxon (which in the vast majority of cases are based on personal examination of specimens) are given in the supplementary information.

### Parsimony Analysis

We first analyzed the dataset using parsimony in TNT v. 1.1^48^. Four outgroups were scored and included in the analysis. In keeping with the previous practice of our prior tyrannosauroid analysis^3^ and TWiG coelurosaur phylogenetic analyses [e.g., ref. 39], *Allosaurus* was used to root the tree in the primary analysis. However, as a sensitivity analysis, we also experimented with using the other three outgroups to root the tree, and in all four cases identical results were achieved.

Our search strategy that began with a ‘New Technology’ search (with default parameters for sectorial search, ratchet, tree drift, and tree fusion), which recovered a minimum length tree in 10 replicates. This process serves to broadly sample tree space and identify major tree islands. This returned four most parsimonious trees (MPTs) (765 steps, consistency index = 0.557, retention index = 0.812). These trees were then subjected to additional TBR branch swapping, which more fully explores each of the tree islands identified in the New Technology search. This procedure recovered an additional most parsimonious tree. Therefore, five total MPTs were recovered, and their common components were summarized in a strict consensus (Fig. 1). *Aviatyrannis*, known from just a single bone, was identified as a wildcard and removed from the strict consensus a posteriori. Jackknife resampling (1,000 replicates, 36% character removal) and Bremer values were used to assess clade support. The analysis was run both with and without the problematic taxon *Raptorex*^36^,^37^, and the topology of the remaining tyrannosauroids was identical in both cases.

We consider the results of the parsimony analysis to be our overall favored topology, because maximum parsimony is the standard method for reconstructing phylogeny using morphological characters in fossil vertebrates, relies on the fewest assumptions about how discrete morphological characters evolve, and is the method that all previous tyrannosauroid analyses have used (so therefore our parsimony results can be most easily compared to previous studies).

### Bayesian Analysis

We also analyzed our dataset using Bayesian analysis in Mr Bayes V3.2.5^49^. We followed the standard protocol employed by the few studies thus far that have applied Bayesian analyses to reconstruct the phylogeny of fossil vertebrates using morphological data [e.g., refs 22, 23], which based their analytical strategies on the recommendations of Lewis^21^. These recommendations outline a series of steps that model the evolution of discrete characters in simple framework, using a minimum of assumptions.

We applied Lewis’ Mk model, which assumes that a morphological character can change its state at any time, with equal probability for all time intervals along a branch. In doing so, we set our datatype as ‘standard’, which permits a variable number of states for each character, employed the 4by4 evolutionary model (the standard substation model), and specified that all substitutions have the same rates (nst = 1, which assumes equal character state frequencies and equal transition rates between states, essentially equivalent to the Jukes-Cantor^50^ and Felsenstein81^51^ models for molecular sequence evolution). All other parameters were set to their defaults, with one exception: we first ran the analysis assuming equal rates of character change (rates = equal) and then ran a second version that employed a gamma shape parameter, which allows for variable rates of character change (rates = gamma, ngammacat = 4), following Nylander^52^. For both analyses we ran 1,000,000 generations (four chains, two independent runs), with a tree sampled every 100 generations. The first 25% of samples were discarded as burn-in. Stationarity was achieved with a standard deviation of split frequencies less than 0.012.

We selected a preferred Bayesian topology by comparing the results of our two analyses (i.e., with and without the inclusion of the gamma parameter). This was done by comparing the harmonic means of the log-likelihood of each of the two analyses. The analysis with the harmonic mean closest to 0 is preferred. This serves to determine whether the addition of the gamma parameter improved the fit of the model of evolution to the data^53^. The harmonic mean of the analysis without a gamma parameter was −2745.57, and that of the analysis with the gamma parameter was −2726.15. The significance of this difference was checked by calculating a Bayes factor, which is two times the difference in the harmonic means. A value greater than 10 is generally considered as strong support that one analysis is a better fit to the data than the other^54^. In our case, the Bayes factor was 38.84, which is deemed significant. As a result, we selected the analysis with the gamma parameter as our preferred Bayesian topology, as its harmonic mean is closer to zero and the Bayes factor comparing it to the gamma-free analysis is significant. This topology is shown in Fig. 2.

### Data archiving

All primary data, including the phylogenetic character list and character-taxon matrix, are available in the supplementary information.

## Additional Information

**How to cite this article**: Brusatte, S. L. and Carr, T. D. The phylogeny and evolutionary history of tyrannosauroid dinosaurs. *Sci. Rep.* **6**, 20252; doi: 10.1038/srep20252 (2016).

## Supplementary Material

Supplementary Information

Supplementary Dataset 1
