# Supplementary material for: The phylogeny and evolutionary history of tyrannosauroid dinosaurs
Source: Sci Rep. 2016 Feb 2;6:20252. doi: 10.1038/srep20252 (PMC4735739; doi:10.1038/srep20252)
Supplement: Supplementary Information [file srep20252-s1.pdf]

Supplementary information for:

The phylogeny and evolutionary history of tyrannosauroid dinosaurs

Stephen L. Brusatte[1#*] & Thomas D. Carr[2#]

[1]School of GeoSciences, University of Edinburgh, Grant Institute, James Hutton Road, Edinburgh EH9 3FE, UK
[2]Department of Biology, Carthage College, 2001 Alford Park Drive, Kenosha, WI 53140, USA
[#]Authors contributed equally and are listed alphabetically.
*Correspondence to: Stephen.Brusatte@ed.ac.uk

1. Materials and Methods
2. Character List
3. Phylogenetic Taxon-Character Matrix
4. Comments on Loewen *et al*. (2013)
5. Extended Discussion on the Tyrannosaurid Body Plan
6. References

1. Materials and Methods

Background: The phylogenetic dataset presented here is an updated version of the dataset of Brusatte et al. (2010). This dataset was originally published by the authors (SLB and TDC) and later updated with the addition of new characters by Brusatte and Benson (2013) and Lü et al. (2014).

Several major developments in the field of tyrannosauroid palaeontology have occurred since the publication of Brusatte et al. (2010). A major alternative phylogenetic dataset, compiled by an independent team of researchers, was published by Loewen et al. (2013). Five new tyrannosauroids were discovered and named: *Lythronax* (Loewen et al. 2013), *Nanuqsaurus* (Fiorillo and Tykoski 2014), *Qianzhousaurus* (Lü et al. 2014), *Yutyrannus* (Xu et al. 2012), and *Zhuchengtyrannus* (Hone et al. 2011). The British taxon *Stokesosaurus langhami* was removed from the genus *Stokesosaurus*, based on a holotype from North America, and transferred to a new genus, *Juratyrant* (Brusatte and Benson 2013). Important new material of *Teratophoneus* was described by Loewen et al. (2013), greatly expanding the available morphological data for this taxon. It was recognized that a specimen of *Daspletosaurus* does not belong to the type species, *D. torosus*, but is a new taxon (Carr and Varricchio 2014). The status of the controversial small-bodied *Raptorex* was reviewed and this taxon was shown to be latest Cretaceous in age (Fowler et al. 2011), not Early Cretaceous as originally described (Sereno et al. 2009).

The New Dataset: The wealth of new data described above is incorporated into the phylogenetic dataset presented in this paper. The five new tyrannosauroids have been added, *Daspletosaurus* has been split into two OTUs, and the new material of *Teratophoneus* has been used to score this taxon. We have also critically reviewed the characters in the Loewen et al. (2013) dataset, accepting many and incorporating these into our dataset, but discarding those that are already in our dataset or are invariant among tyrannosauroids, and those that we think are problematic (e.g., because they are redundant with each other or difficult for us to understand; see our rationale in Section 4 below). We consider the resulting dataset presented in this paper to be a merging of the Brusatte et al. (2010) and Loewen et al. (2013) datasets, although we recognize that other workers may merge the two datasets slightly differently, given the subjectivity that can be associated with the writing and scoring of morphological cladistic characters.

Our new dataset presented here includes 366 discrete anatomical characters scored for 28 tyrannosauroids and four outgroups. The full character set is listed in Section 2 below. The first 307 characters are from Brusatte et al. (2010), six are characters added by Brusatte and Benson (2013) and three added by Lü et al. (2014), seven are completely new characters used here for the first time (gleaned from our comparative studies of tyrannosauroid anatomy as part of this project), 36 are from Loewen et al. (2013) and incorporated into our dataset for the first time, and eight are new characters outlined by Carr and Varricchio (2014).

We include all species-level tyrannosauroids that are valid taxa, with a few exceptions. *Coelurus* and *Tanycolagreus* may be basal tyrannosauroids (e.g., Senter 2007; Brusatte et al. 2014), but because this has yet to be established convincingly they are not included here. *Alectrosaurus* is excluded because the lectotype is a single hindlimb with incomplete pelvic material, which limits the comparisons that can be made, and the referral of other fossils to the taxon has not been convincingly made (Perle 1977; Mader and Bradley 1990). *Alectrosaurus* is currently under monographic study by TDC and will be incorporated into our dataset after this

study is finished. *Bagaraatan* is likely a chimaera of specimens belonging to tyrannosauroids and other taxa (see discussion in Brusatte 2013), so is excluded. Recent work has suggested that megaraptorans may be nested within Tyrannosauroidea (Novas et al. 2013), but this is currently a minority view that has been recoved by one working group and remains to be properly tested in a higher-level theropod analysis that includes a wealth of data pertinent to coelurosaurs and basal tetanurans, analyzed simultaneously. Therefore, we do not include megaraptorans here but are open to the possibility that they may be tyrannosauroids.

Scoring Sources: The vast majority of taxa and characters were scored based on first-hand examination of specimens by SLB and/or TDC. The following sources were used to score the tyrannosauroid taxa in our dataset. The author who was in charge of scoring each taxon is also listed (in all cases the other author reviewed the scores).

Kileskus: ZIN (Paleoherpetological Collection, Zoological Institute of the Russian Academy of Sciences, St. Petersburg, Russia) PH 5/117—13/117. Observed and scored by SLB. Additional data from Averianov et al. (2010).

Guanlong: IVPP (Institute of Vertebrate Paleontology and Paleoanthropology, Beijing, China) V14531, V14532. Observed and scored by SLB. Additional data from Xu et al. (2006).

Proceratosaurus: NHMUK (Natural History Museum, London, England, UK) R4860. Observed and scored by SLB. Additional data from Rauhut et al. (2010).

Yutyrannus: ZCDM (Zhucheng Dinosaur Museum, Shandong, China) V5000—50001 and ELDM (Erlianhaote Dinosaur Museum, Inner Mongolia, China) V0001. Observed and scored by SLB. Additional data from Xu et al. (2012).

Dilong: IVPP 14243. Observed and scored by SLB. Additional data from Xu et al. (2004).

Sinotyrannus: We have not been able to observe this taxon personally and base all scores on Ji et al. (2009).

Eotyrannus: IWCMS (Dinosaur Isle Visitor Centre, Sandown, Isle of Wight, England, UK) 1997.550. Observed and scored by SLB. Additional data from Hutt et al. (2001) and Naish et al. (2001).

Juratyrant: OUMNH (Oxford University Museum of Natural History, Oxford, England, UK) J.3311-1—J.3311-30. Observed and scored by SLB. Additional data from Benson (2008) and Brusatte and Benson (2013).

Aviatyrannis: We have not been able to observe this taxon personally and base all scores on Rauhut (2003).

Stokesosaurus: UMNH (Utah Museum of Natural History, Salt Lake City, Utah, USA) VP 7434, VP 6386. Observed and scored by SLB.

Xiongguanlong: We have not been able to observe this taxon personally and base all scores on Li et al. (2010) and high-resolution photos provided by Peter Makovicky.

Raptorex: LH (Long Hao Institute of Geology and Paleontology, Hohhot, Inner Mongolia, China) PV18. Observed and scored by SLB and TDC. Additional data from Sereno et al. (2009).

Dryptosaurus: ANSP (Academy of Natural Sciences, Philadelphia, Pennsylvania, USA) 9995 and AMNH (American Museum of Natural History) FARB 2438. Observed and scored by SLB and TDC. Additional data from Carpenter et al. (1997) and Brusatte et al. (2011).

Appalachiosaurus: RMM (McWane Center, Birmingham, Alabama, USA) 6670. Observed and scored by TDC. Additional data from Carr et al. (2005).

Albertosaurus: AMNH FARB 5222; CMN (Canadian Museum of Nature, Aylmer, Quebec, CAN) 5600, 5601; ROM (Royal Ontario Museum, Toronto, Ontario, CAN) 807; TMP (Royal Tyrrell Museum of Palaeontology, Drumheller, Alberta, CAN) 1981.010.0001, 1985.098.0001, 1986.064.0001, 1998.063.0088. Observed and scored by TDC.

Gorgosaurus: AMNH FARB 5336, 5458, 5664; CMN 2120; ROM 1247; TMP 1986.144.0001, 1991.036.0500, 1994.012.0602, 1999.033.0001, 2000.012.0011, 2009.012.0014; UALVP (University of Alberta Vertebrate Paleontology, Edmonton, Alberta, CAN) 10. Observed and scored by TDC.

Alioramus remotus: We have not been able to observe this taxon personally, and base all scores on Kurzanov (1976) and high-resolution photos provided by Philip Currie, Tetsuto Miyashita, Robert Reisz, and Alexander Averianov.

Alioramus altai: IGM (Institute of Geology, Ulaan Baatar, Mongolia) 100/1844. Observed and scored by SLB and TDC. Additional data from Brusatte et al. (2009, 2012).

Qianzhousaurus: GM (Ganzhou Museum, Ganzhou City, Jiangxi Province, China) F10004-1—10004-8. Observed and scored by SLB. Additional data from Lü et al. (2014).

Daspletosaurus: AMNH FARB 5346; CMN 8605, 11594; FMNH PR (Field Museum of Natural History, Chicago, Illinois, USA) 308; TMP 1985.062.0001, 1994.143.0001, 2001.036.0001. Observed and scored by TDC.

Daspletosaurus n. sp.: MOR (Museum of the Rockies, Bozeman, Montana, USA) 590, MOR 1130. Observed and scored by TDC.

Tarbosaurus: ZPAL (Institute of Paleobiology of the Polish Academy of Sciences, Warsaw, Poland) and PIN (Borissiak Paleontological Institute of the Russian Academy of Sciences, Moscow, Russia) collections of numerous *Tarbosaurus* individuals. Observed by SLB and scored by SLB and TDC.

Tyrannosaurus: AMNH FARB 5027, 5117, 5029; CM (Carnegie Museum of Natural History, Pittsburgh, Pennsylvania, USA) 9380; FMNH PR2081; LACM (Los Angeles County Museum of Natural History, Los Angeles, California, USA) 23844, 23845; MOR 555, 980;, RSM (Royal Saskatchewan Museum, Eastend, Saskatchewan, CAN) 2523.8; TMP 1981.006.00001. Observed and scored by TDC.

Bistahieversor: NMMNH (New Mexico Museum of Natural History and Science, Albuquerque, New Mexico) P-27469, P-25049, P-32824; OMNH (Oklahoma Museum of Natural History) 10131. Observed and scored by TDC. Additional data from Carr and Williamson (2010).

Teratophoneus: BYU (Brigham Young University, Provo, Utah, USA) 8120/9396, 8120/9397, 826/9402, 9398, 13719. Observed and scored by TDC. Additional data from high-resolution photographs of UMNH VP 16690 provided by Mark Loewen and Loewen et al. (2013), and from Carr et al. (2011).

Lythronax: We have not been able to observe this taxon personally, and base all scores on high-resolution photographs of UMNH VP 20200 provided by Mark Loewen and Loewen et al. (2013).

Zhuchengtyrannus: ZCDM V0031. Observed by SLB and scored by SLB and TDC. Additional data from Hone et al. (2011).

Nanuqsaurus: We have not been able to observe this taxon personally, and base all scores on Fiorillo and Tykoski (2014).

Parsimony: Analytical Protocols and Results: The dataset was subjected to a parsimony analysis in TNT v. 1.1 (Goloboff et al. 2008). *Allosaurus* was used as the outgroup to root the tree (we also performed sensitivity analyses in which the other three outgroups were used to root the tree instead of *Allosaurus*, and each of these three analyses returned identical results to when *Allosaurus* was used for rooting). We first analyzed the matrix with a 'New Technology Search', using sectorial search, ratchet, tree drift, and tree fuse options with default parameters. The minimum length tree was found in 10 replicates. This process aims to sample as many tree islands as possible, and recovered four most parsimonious trees (MPTs) of 765 steps (consistency index=0.557, retention index=0.812). These four trees were then subjected to traditional TBR branch swapping, which more fully explores each tree island. This procedure found an additional MPT. Therefore, five total MPTs were recovered. The poorly known *Aviatyrannis*, known from just a single bone, was identified as a wildcard and pruned from the strict consensus a posteriori. Clade support was quantified using Bremer support values and jackknife scores (1000 replicates, 36% character removal probability).

We also ran the phylogenetic analysis by removing *Raptorex* a priori but keeping all other analytical protocols the same. *Raptorex* is a controversial taxon because its holotype and only known specimen is a very young individual (Sereno et al. 2009; Fowler et al. 2011). This exercise showed that the topology of the remaining tyrannosauroids is identical whether *Raptorex* is included or not, indicating that this taxon has little bearing on the phylogentic relationships of other tyrannosauroids.

Bayesian: Analytical Protocols and Results: We also subjected our dataset to Bayesian analysis in Mr Bayes V3.2.5 (Ronquist et al. 2011). We employed a series of steps, recommended by Lewis (2001), that model the evolution of discrete characters in a simple framework, using a minimum of assumptions. We applied Lewis' Mk model, which assumes that a morphological character can change its state at any time, with equal probability for all time intervals along a branch. In doing so, we set our datatype as 'standard', which permits a variable number of states for each character, employed the 4by4 evolutionary model (nucmodel=4by4, the standard substation model), and specified that all substitutions have the same rates (nst=1, which assumes equal character state frequencies and equal transition rates between states, essentially equivalent to the Jukes-Cantor [Jukes and Cantor 1969] and Felsenstein81 [Felsenstein 1981] models for molecular sequence evolution). All other parameters were set to their defaults, with one exception: we first ran the analysis assuming equal rates of character change (rates=equal) and then ran a second version that employed a gamma shape parameter, which allows for variable rates of character change (rates=gamma, ngammacat=4). For both analyses we ran 1,000,000 generations (four chains, two independent runs), with a tree sampled every 100 generations (mcmcp ngen=1000000 nruns=2 nchains=4 samplefreq=100). The first 25% of samples were discarded as burn-in (mcmcp burninfrac=0.25). Stationarity was achieved with a standard deviation of split frequencies less than 0.012.

We selected a preferred Bayesian topology by comparing the results of our two analyses (i.e., with and without the inclusion of the gamma parameter). This was done by comparing the harmonic means of the log-likelihood of each of the two analyses. The analysis with the harmonic mean closest to 0 is preferred. This serves to determine whether the addition of the gamma parameter improved the fit of the model of evolution to the data. The harmonic mean of the analysis without a gamma parameter was -2745.57, and that of the analysis with the gamma parameter was -2726.15. The significance of this difference was checked by calculating a Bayes

factor, which is two times the difference in the harmonic means. A value greater than 10 is generally considered as strong support that one analysis is a better fit to the data than the other (Kass and Raftery 1995). In our case, the Bayes factor was 38.84, which is deemed significant. As a result, we selected the analysis with the gamma parameter as our preferred Bayesian topology, as its harmonic mean is closer to zero and the Bayes factor comparing it to the gamma-free analysis is significant.

2. Character List

The following is the full list of characters in the dataset. Multistates are designed as either ordered or unordered. Changes in character wording from Brusatte et al. (2010) are denoted in red text.

Characters 1-307 from Brusatte et al. (2010)

General Skull Features

1) Skull, occipital region, orientation: posteriorly (0); posteroventrally (1). (Carr and Williamson 2010:3)

2) Skull, general shape: long and low, length: height ratio greater than 3.2 (0); deep, length: height ratio less than 3.2 (1). (Carr and Williamson 2010:4) Note: Length is premaxilla-quadrate condyle length; height is maximum height of the upper jaw, not counting any cranial crests.

3) Skull, anteroposterior length: less (0) or greater than (1) 40% trunk length. (Brusatte et al. 2010:3) Trunk length is the anterior extremity of the pectoral girdle to the posterior extremity of the pelvic girdle, as defined by Sereno et al. 2009.

4) External naris, less (0) or greater than (1) 20% of skull length. (Carr and Williamson 2010:54). Note: In the case that an entire skull is not preserved, this character can be scored accurately by comparing the length of the naris to the length of the antorbital fenestra: those taxa with state 0 have a naris that is considerably smaller than the fenestra, whereas those with state 1 have a naris that is approximately the same anteroposterior length as the fenestra (Loewen et al. 2013:1).

5) Lateral temporal fenestra, orientation of long axis relative to long axis of orbit: posterodorsal (0); approximately parallel (1). (Brusatte et al. 2010:5)

Premaxilla

6) Premaxilla, nasal processes of opposing premaxillae, orientation: divergent from each other, with small process of nasals fitting in between them (0); closely appressed to each other (1). (Carr and Williamson 2010:6)

7) Premaxilla, deep foramen or fossa on the lateral surface of the base of the nasal process, within the anteroventral corner of the narial fossa: absent (0); present (1). (Carr and Williamson 2010:7)

8) Premaxilla, main body, dorsoventral depth: less than or equal to (0); between 1-1.9 times (1); greater than 2 times (2) anteroposterior length. ORDERED. (Modified by Brusatte et al. 2010:8 from Carr and Williamson 2009:8 and Sereno et al. 2009:1).

9) Premaxilla, maxillary process orientation: mostly laterally (and resultantly widely visible in lateral view) (0); dorsolaterally (facing almost equally dorsally and laterally) (1); dorsally (and resultantly mostly hidden in lateral view) (2). ORDERED (Carr and Williamson 2010:10).

10) Premaxilla, orientation of tooth row: strongly parasagittally (anteroposteriorly) (0); first two teeth oriented mediolaterally and third and fourth teeth oriented parasagittally (1); entire tooth row oriented mediolaterally and all teeth visible in anterior view (2). ORDERED (Modified by Brusatte et al. 2010:10 from Carr and Williamson 2010:12).

11) Premaxilla, form of narial fossa ventral to external naris: shallowly excavated (0); deeply excavated, anterior margin invaginated as a deep groove (1). (Brusatte et al. 2010:11)

12) Premaxilla, extent of narial fossa: limited to region immediately ventral to external naris (0); extensive, covers most of main body of premaxilla (1). (Brusatte et al. 2010:12)

13) Premaxilla, orientation and shape of anterior margin: smoothly curved and projection posterodorsally, angle between ventral margin of premaxilla and anterior margin is less than 90 degrees (0); smoothly curved and projecting vertically or slightly anterodorsally, angle between ventral margin of premaxilla and anterior margin is equal to or greater than 90 degrees (1); projecting vertically or slightly anterodorsally, with a discrete inflection point between a more vertical ventral portion and a more horizontal dorsal portion (2). ORDERED (Brusatte et al. 2010:13). This character is slightly modified following Brusatte et al. (2014:485). We have transformed the original character of Brusatte et al. (2010:13) into an ordered multistate by adding a new intermediate state referring to an approximately vertical (or slightly anterodorsally inclined) anterior margin of the ventral portion of the premaxilla. State 2 now refers to a special condition of this state, in which the anterior margin is vertical (or anterodorsally inclined) and there is also a discrete inflection point between the more vertical ventral portion of the anterior margin and the more horizontal dorsal portion of the margin. Taxa with this condition, therefore, do not possess a smoothly curved anterior margin of the premaxilla. Tyrannosauroids are characterized by either state 1 or state 2, with state 2 referring to a subset of basal tyrannosauroids (*Dilong*, *Guanlong*, *Kileskus*, *Proceratosaurus*, *Sinotyrannus*).

14) Premaxilla, position of palatal process: immediately above interdental plates (0); separated from interdental plates by deep lingual surface of premaxilla (1). (Brusatte et al. 2010:14)

Maxilla

15) Maxilla, promaxillary fenestra, exposure: visible in lateral view (0); obscured in lateral view by the lateral lamina of the ascending ramus (fenestra faces completely anteriorly) (1). (Carr and Williamson 2010:14)

16) Maxilla, promaxillary fenestra, position: anterior margin of antorbital fossa (0); extreme anteroventral corner of antorbital fossa (1). (Brusatte et al. 2010:16)

17) Maxilla, maxillary fenestra, location: posterior to (0) or partially overlapped laterally by (1) the anterior margin of the antorbital fossa (lateral lamina of maxilla). (Carr and Williamson 2010:15, 16; Sereno et al. 2009:12).

18) Maxilla, maxillary fenestra, location: dorsal to (0) or abuts (1) ventral margin of antorbital fossa. (Sereno et al. 2009:13).

19) Maxilla, maxillary fenestra, anteroposterior length compared to the distance between the anterior margins of the antorbital fossa and fenestra: less than half (0); greater than half (1); greater than half and also greater than half of the length of the eyeball-bearing portion of the orbit (2). ORDERED (Modified by Brusatte et al. 2010:19 from Carr and Williamson 2010:17; Sereno et al. 2009:11).

20) Maxilla, maxillary fenestra, position within maxillary antrum: does not (0) or does (1) abut dorsal border of the antrum in medial view. (Brusatte et al. 2010:20)

21) Maxilla, antorbital fossa, extent: reaches (0) or does not reach (1) the nasal suture. (Carr and Williamson 2010:20)

22) Maxilla, interfenestral strut, anteroposterior length: greater than (0) or less than (1) 50% of the long axis of the maxillary fenestra. (Modified by Brusatte et al. 2010:22 from Carr and Williamson 2010:22)

23) Maxilla, main body, dorsoventral depth at midpoint of antorbital fenestra: less than (0) 16%; between 16-22% (1); or greater than (2) 22% depth of skull at midpoint of antorbital fenestra. ORDERED. (Modified by Brusatte et al. 2010:23 from Carr and Williamson 2010:23)

24) Maxilla, antorbital fossa, trend of dorsoventral depth across main body: uniform depth (0); diminishes in depth posteriorly (1). (Carr and Williamson 2010:25)

25) Maxilla, subcutaneous flange bordering the antorbital fossa laterally on the posterior end of the main body, resulting in the fossa forming a channel between the flange and the main body: absent (0); present (1). (Carr and Williamson 2010:26)

26) Maxilla, dorsolateral process, coverage by antorbital fossa: process absent (0); process covered by subcutaneous surface only (1); ventral half of process covered by antorbital fossa (2); antorbital fossa completely excluded (3). ORDERED. (Carr and Williamson 2010:28)

27) Maxilla, narrow region of smooth surface texture between anterior margin of antorbital fossa and the subcutaneous surface: absent (0); present (1). (Carr and Williamson 2010:29)

28) Maxilla, ventral margin of the anterior region of the bone, profile: straight (0); convex (1). (Carr and Williamson 2010:30; Sereno et al. 2009:14). Note: Loewen et al. (2013) divided our “convex” state into two states: “slightly” and “highly” convex. This difference was not quantified, and we have been unable to find a clear numerical cut-off between different degrees

of convexity, so we retain our original character language and score all taxa with a convex profile identically.

29) Maxilla, joint surface for palatine, depth: shallow, does not obscure the tooth root bulges from view (0); deep, obscures tooth root bulges from view (1). (Carr and Williamson 2010:31)

30) Maxilla, anterior ramus (demarcated by concave step in anterior margin of maxilla): absent (0); present (1). (Brusatte et al. 2010:30)

31) Maxilla, form of contact with nasal in subadult to adult specimens: smooth (0); weakly scalloped (1); deeply scalloped with interlocking transverse ridges on both elements (2). ORDERED (Modified by Brusatte et al. 2010:31 from Sereno et al. 2009:15).

32) Maxilla, form of external subcutaneous surface texturing: random foramina and shallow grooves and ridges (0); deep, prominent, dorsoventrally trending grooves and ridges (1), extremely coarse texture (2). UNORDERED (Modified from Brusatte et al. 2010:32 by the addition of a new state)

33) Maxilla, swollen rim separating antorbital fossa and subcutaneous surface: present (0); absent (1). (Brusatte et al. 2010:33)

34) Maxilla, size of ascending ramus, anteroposterior chord directly above maxillary fenestra compared to dorsoventral depth of maxilla below anterior edge of antorbital fenestra: greater than 1.75 times (ascending ramus large) (0); less than 1.60 times (ascending ramus small) (1). (Brusatte et al. 2010:34)

35) Maxilla, posterior region of the main body (portion including the final 3-5 teeth and anterior to the jugal process), shape: maintains a relatively constant dorsoventral depth (0); tapers in depth posteriorly (1). (Brusatte et al. 2010:35)

36) Maxilla, primary row of neurovascular foramina, form: continues as a row of discrete foramina posteriorly (0); transitions into a sharp groove, paralleling the antorbital fossa rim, posteriorly (1). (Brusatte et al. 2010:36)

37) Maxilla, antorbital fossa, extent on main body under midpoint of antorbital fenestra: covers more than 65% (0), between 50-60% (1), or less than 45% (2) of the depth of the bone. ORDERED (Modified version of Brusatte et al 2010: 37 using character scores in Loewen et al. 2013:42)

Nasal

38) Nasals, shape of dorsal surface: flat or slightly convex (0); convex (vaulted) anteriorly, above and immediately posterior to the external naris (1); vaulted across most of their length (2). ORDERED (Modified by Brusatte et al. 2010:38 from Carr and Williamson 2010:37; Sereno et al. 2009:5). Note that crested taxa are scored as inapplicable (“?”).

39) Nasals, midline crest on dorsal surface: absent (0); present (1). (Carr and Williamson 2010:50)

40) Nasal, external texture of midsection of bone: smooth to slightly rugose (0); pronounced rugosities and accessory vascular foramina present (1). (Carr and Williamson 2010:48; Sereno et al. 2009:7)

41) Nasal, shape in dorsal view: expands in width posteriorly (0); relatively constant width across the length of the bone, due to subparallel lateral sides (1); tapers in width posteriorly (2). UNORDERED (Sereno et al. 2009:6)

42) Nasal, frontal process, mediolateral width: unconstricted (0); constricted, less than ½ width of widest point of nasal (1). (Carr and Williamson 2010:41)

43) Nasal, internal pneumatic recess: present (0); absent (1). (Carr and Williamson 2010:39)

44) Nasal, posterolateral process that underlaps the tip of the lacrimal: absent (0); present (1). (Carr and Williamson 2010:40; Sereno et al. 2009:4)

45) Nasal, extent of narial fossa on premaxillary process: limited to ventral margin of process (0); covers entire process, and thus meets opposite fossa on dorsal midline (1). (Sereno et al. 2009:2)

46) Nasal, medial processes of frontal articulation, shape: processes absent (0); lanceolate (1); tapered (2). UNORDERED (Carr and Williamson 2010:44)

47) Nasal, thin, low, and laterally projecting crest at the corner where lateral and dorsal surfaces meet: absent (0); present (1). (Brusatte et al. 2010:47) Note: The derived state describes the Y-shaped crest of *Dilong*, originally described as an autapomorphy, which is also present (albeit in as a much smaller and less pronounced structure) in *Eotyrannus*.

Lacrimal

48) Lacrimal, shape in lateral view: inverted L, angle between anterior and ventral rami approximately 90 degrees (0); 7 shaped, angle between anterior and ventral rami 70-80 degrees (1). (Carr and Williamson 2010:55).

49) Lacrimal, cornual process on dorsal surface: present as a broad, shallow, dorsally convex, laterally overhanging swelling across most of the length of the anterior ramus (0); present as a discrete conical projection (1); small, conical, smooth projection that rises 2-3 millimeters from skull roof (2). UNORDERED (Modified by Brusatte et al. 2010:49 from Carr and Williamson 2010:56; Sereno et al. 2009:18). This character has been modified further here, as the original Brusatte et al. (2010:49) character has been split into two: the current character and character 318 in this dataset.

50) Lacrimal, cornual process, form: smoothly rounded (0); discrete apex present (1). (Carr and Williamson 2010:57)

51) Lacrimal, cornual process, position of apex: dorsal to ventral ramus (0); anterior to ventral ramus (1). (Carr and Williamson 2010: 9; Sereno et al. 2009:19)

52) Lacrimal, pneumatic foramina opening laterally at the junction of the anterior and ventral processes above the antorbital fenestra: absent (0); present, extent of pneumaticity limited to partially hollowing the bone in the region where the anterior and ventral rami meet (1); present and extensive, completely hollowing the bone where the anterior and ventral rami meet (2). ORDERED (Sereno et al. 2009:16) We here modify this character following Brusatte et al. (2014:38), making the description more specific than in Brusatte et al. (2010:52).

53) Lacrimal, anterior ramus: not inflated (0); inflated by pneumatic recess (1). (Carr and Williamson 2010: 64; Sereno et al. 2009:17).

54) Lacrimal, size of primary external opening for lacrimal recess: small, anterior end located approximately at the same level as the anterior end of the ventral ramus (0); large, anterior end located far anterior to the ventral ramus (1). (Carr and Williamson 2010:60)

55) Lacrimal, lacrimal pneumatic recess, transition between antorbital fossa and the subcutaneous surface of the ventral ramus, form, lateral view: surfaces are continuous with each other (0); fossa is deeply inset, forming a ridge along the subcutaneous surface (1). Note: The description of this character has been modified from Brusatte et al. (2010:55) in order to make it clearer. This has necessitated some taxa being scored differently.

56) Lacrimal, accessory external openings for lacrimal recess on the anterior ramus: absent (0); present (1). (Modified from Brusatte et al. 2010:56). Note: The original Brusatte et al. (2010:56) character was a complex multistate character having to do with both the presence/absence and position of the external pneumatic openings in front of the main lacrimal pneumatic foramen. Upon reexamination, we find that there is incredible variation in the position of the accessory openings, including individual and ontogenetic variation, which is often the case for pneumatic features. We here change this character to a simpler presence/absence character relating to the accessory openings.

57) Lacrimal, pneumatic recess opening internally onto medial surface of bone as a discrete pneumatic fenestra (pneumatopore): absent (0); present (1). (Carr and Williamson 2010:68) Note that this character has been rewritten slightly so that it refers specifically to the presence/absence of a large pneumatic opening on the medial surface of the lacrimal, following the description in Gold et al. (2013).

58) Lacrimal, dorsal prong of anterior ramus for articulation with maxilla (“anterodorsal process”): absent or small (0); present and elongate (1). (Modified by Brusatte et al. 2010:58 by Carr and Williamson 2010:65)

59) Lacrimal, ventral ramus, extent of medial lamina: greater (0) or less than (1) half of the dorsoventral depth of the ramus. (Carr and Williamson 2010:72)

60) Lacrimal, orbitonasal ridge on medial surface, position: anterior (0) or adjacent to or contacting (1) posterior margin of ventral ramus. (Carr and Williamson 2010:69).

61) Lacrimal, articulation with frontal, form: squamous (0); conical lacrimal process set into deep pit in frontal (1). (Sereno et al. 2009:22)

62) Lacrimal, posterior process for articulation with frontal, inflated by pneumatic recess: no (0); yes (1). (Brusatte et al. 2010:62)

63) Lacrimal, extent of antorbital fossa on ventral ramus: covers greater (0) or less than (1) 60% of anteroposterior length along the contact with the jugal. (Brusatte et al. 2010:63)

64) Lacrimal, maxillary process of anterior ramus, visibility in lateral view: both dorsal and ventral margins visible (0); dorsal margin concealed by subcutaneous surface above antorbital fossa and only ventral margin visible (1). (Carr and Williamson 2010:66)

Jugal

65) Jugal, maxillary ramus, depth: shallow, not expanded relative to suborbital portion of bone (0); deep, expanded relative to suborbital portion of bone (1). (Carr and Williamson 2010:73)

66) Jugal, antorbital fossa, extent on maxillary ramus: edge of fossa undercut and continues posterodorsal to jugal recess (0); fossa edge does not extend past the jugal recess (1). (Carr and Williamson 2010:75)

67) Jugal, pneumatic recess, location relative to ventral ramus of lacrimal: ventral (0); anterior (1). (Carr and Williamson 2010:76)

68) Jugal, pneumatic recess, orientation of long axis: approximately horizontal (0); inclined at approximately 45 degrees relative to the ventral skull margin (1). (Carr and Williamson 2010:78)

69) Jugal, secondary fossa for pneumatic recess, position relative to recess: ventral (0); dorsal (1). (Carr and Williamson 2010:80)

70) Jugal, suture with lacrimal, angle of the posterior half of the contact: low (0); steep (1). (Carr and Williamson 2010:81)

71) Jugal, fossa on lateral surface of postorbital ramus, depth inset into bone: shallow (0); deep (1). (Carr and Williamson 2010:82; Sereno et al. 2009:24)

72) Jugal, articulation with postorbital, form of ventral extremity of suture: tapering scarf joint (0); shallow interlocking notch for postorbital (1); deep interlocking notch for postorbital, defined by a flange anterior to the notch on the jugal (2). ORDERED (Carr and Williamson

2010:83; Sereno et al. 2009:26) Modified here to separate the ‘notch’ character of Brusatte et al. (2010:72) into two states (shallow vs. deep) following Loewen et al. (2013:137).

73) Jugal, articulation with postorbital, extent of scarf joint on lateral surface of postorbital ramus: limited, occupies less than 50% of anteroposterior length of the process (0); extensive, occupies approximately 50-75% of the anteroposterior length of the process (1). (Brusatte et al. 2010:73)

74) Jugal, articulation with postorbital, braced by a pronounced ridge on the lateral surface of the postorbital ramus, which borders the postorbital posteriorly: no (0); yes (1). (Brusatte et al. 2010:74)

75) Jugal, postorbital ramus, orientation relative to ventral margin of jugal: approximately perpendicular (0); posterodorsal (obtuse angle between the long axis of the process and the ventral margin) (1). (Brusatte et al. 2010:75)

76) Jugal, cornual process: absent (0); present (1); present and distinctive (mediolaterally thick and heavily rugose (2). ORDERED (Carr and Williamson 2010:84, 85)

77) Jugal, dorsal prong of quadratojugal ramus, slope in lateral view: horizontal (0); posterodorsal (1). (Carr and Williamson 2010:91)

78) Jugal, ventral prong of quadratojugal ramus, slope of joint surface in lateral view: approximately anteroposteriorly oriented, angled less than 45 degrees from horizontal (0); angled anterodorsally at greater than 45 degrees from horizontal (1). (Carr and Williamson 2010:90)

79) Jugal, shape of orbital margin, weakly concave, approximately level with lacrimal-jugal suture (0); U-shaped, extends ventral to lacrimal-jugal suture (1). (Sereno et al. 2009:26)

80) Jugal, raised rim on the lateral surface, paralleling the ventral margin of the bone and anteriorly confluent with the antorbital fossa rim of the maxilla: absent (0); present (1). (Brusatte et al. 2010:80)

Postorbital

81) Postorbital, cornual process: absent (0); limited to rugose rim at posterodorsal corner of orbit (1); present as a rugose, convex boss (2). ORDERED (Sereno et al. 2009:29)

82) Postorbital, cornual process, position: separated from dorsal margin of postorbital by a smooth, convex region (0); approaches or extends past dorsal margin of bone (1). (Carr and Williamson 2010:94).

83) Postorbital, cornual process, position: located at orbital margin (0); located posterodorsal to orbital margin (1). (Carr and Williamson 2010:95)

84) Postorbital, squamosal ramus, form of posterodorsal margin: uninterrupted convex arc or marked by very shallow concave notch (0); emarginated by squamosal (discrete concave notch within the margin) (1). (Carr and Williamson 2010:99)

85) Postorbital, squamosal ramus, extent: reaches or extends posterior to (0) or terminates anterior to (1) posterior margin of lateral temporal fenestra. (Carr and Williamson 2010:100; Sereno et al. 2009:28)

86) Postorbital, suborbital process that extends into the orbit: absent, ventral ramus tapers (0); present and large in adults, small and unpronounced (ventral ramus tongue-shaped) in sub-adults (1); present and large in sub-adults and adults (2). ORDERED (Modified by Brusatte et al. 2010:86 from Carr and Williamson 2010:101; Sereno et al. 2009:30)

87) Postorbital, suborbital process, position: at ventral end of ventral process (0); flange-like, separated from ventral tip of ventral process by a notch (1). (Carr and Williamson 2010:102)

88) Postorbital, anterior ramus, form: short and stout, long axis is approximately half the length of the ventral ramus and the thickness at the base is approximately the same as the thickness of the midpoint of the ventral ramus (0); long and slender, long axis is greater than 60% of the length of the ventral ramus and the thickness at the base is approximately half that of the midpoint of the ventral ramus (1). (Li et al. 2010:252).

89) Postorbital, ventral ramus, anteroposterior width at midpoint: approximately the same width as (0) or substantially wider than (1) ventral ramus of the lacrimal. (Li et al. 2010:268).

Squamosal

90) Squamosal, lateral ridge delimiting supratemporal fossa, form: ridge unpronounced or undivided (0); divided (1). (Carr and Williamson 2010:106)

91) Squamosal, supratemporal fossa, surface morphology: flat or concave (0); convex (1). (Carr and Williamson 2010:107)

92) Squamosal, quadratojugal process, orientation of long axis: dorsoventral or slightly oblique (0); anteroposterior (1). (Carr and Williamson 2010:111)

93) Squamosal, quadratojugal process, morphology of anterior tip in those taxa with horizontal processes: tapered point (0); squared off (1). (Carr and Williamson 2010:110)

94) Squamosal, quadratojugal process, flange that is covered laterally by the quadratojugal, dorsoventral depth of entire process compared to portion of process that is exposed in lateral view when in articulation with quadratojugal: flange absent (0); thinner (1); substantially thicker (2). ORDERED (Carr and Williamson 2010:112)

95) Squamosal, posterior process, inflated by squamosal recess: no (0); yes (1). (Carr and Williamson 2010:114)

96) Squamosal, posterior process, length of the long axis compared to the long axis of the quadratojugal process: long, approximately 1/3-1/2 (0); short, approximately 1/6 (1). (Carr and Williamson 2010:115).

97) Squamosal, anterior process, flange that extends dorsal to the postorbital posterior process: absent (0); present (1). (Sereno et al. 2009:32)

Quadratojugal

98) Quadratojugal and squamosal, constriction of lateral temporal fenestra: absent, anterior margins of both bones are approximately vertical (0); present, convex kink along the suture between the two bones that projects into the fenestra, constricting it to approximately one half of its maximum anteroposterior length (1); present, dorsal region of quadratojugal moderately expanded anteroposteriorly relative to the remainder of the bone, constricting fenestra to approximately one half of its maximum anteroposterior length (2); present, dorsal region of quadratojugal expanded anteroposteriorly by at least twice the minimum anteroposterior dimension of the bone, forming a flange that meets the ventral ramus of the squamosal to nearly divide the fenestra (3). ORDERED (Carr and Williamson 2010:116; Sereno et al. 2009:35).

99) Quadratojugal, dorsal process, ridge along anterior margin of lateral surface: absent (0); present, subtle and fades in strength dorsally (1); present, robust and extends to the dorsal margin of the bone (2). ORDERED. (Modified by Brusatte et al. 2010:99 from Carr and Williamson 2010:117)

100) Quadratojugal, form of jugal articulation: dorsal prong of posterior process of jugal does not (0) or does (1) approach the base of the quadratojugal (the corner where the anterior and dorsal processes of the quadratojugal meet). (Carr and Williamson 2010:120)

101) Quadratojugal, anterior process for articulation with jugal, form of anterior region: tapered (0); rounded (1); squared off or double pronged (2). UNORDERED (Carr and Williamson 2010:121; Sereno et al. 2009:34)

102) Quadratojugal, anterior process, extent: terminates posterior (0) or level with or anterior (1) to anterior margin of lateral temporal fenestra. (Carr and Williamson 2010:88; Sereno et al. 2009:36)

103) Quadratojugal, deep pocket bordering quadrate foramen laterally visible in posterior view, defined by posterolateral edge of quadratojugal shaft curving posteriorly instead of being mediolaterally compressed and flat: absent (0); present (1). (Li et al. 2010: 281) Note that the character description is modified here, relative to Brusatte et al. (2010:103), to make it clearer.

104) Quadratojugal, posterior process, length and orientation: short, oriented mostly laterally (0); elongate, wraps onto the posterior surface of the quadrate condyles (1). (Brusatte et al. 2010:104)

Quadrate

105) Quadrate foramen, size: small, long axis approximately 10% of the dorsoventral depth of the quadrate shaft (0); large, long axis greater than 20% of the dorsoventral depth of the quadrate shaft (1). (Carr and Williamson 2010:125)

106) Quadrate, pneumaticity: absent (0); present (1). (Carr and Williamson 2010:126). This character is modified here, so that the original character of Brusatte et al. (2010:106) is divided into two: one having to do with presence/absence of quadrate pneumaticity (character 106 here) and one having to do with the presence/absence of a deep recess on the anterior surface of the bone leading into the internal pneumatic cavity (character 317 here).

107) Quadrate condyles, position relative to occipital condyle when skull is in articulation: approximately aligned in the dorsoventral plane (0); completely posterior (1). (Carr and Williamson 2010:127; Sereno et al. 2009:42)

108) Quadrate, quadratojugal articulation, extent on lateral surface of lateral condyle: limited, occupies only part of the surface (0); extensive, covers entire lateral surface and extends dorsally to partially enclose quadrate foramen laterally (1). (Brusatte et al. 2010:108)

109) Quadrate, articular surface for quadratojugal on quadrate lateral condyle, orientation of medial margin as seen in posterior view where quadratojugal wraps around quadrate: vertical or dorsomedial (0); dorsolateral (1). (Carr and Williamson 2010:124)

Prefrontal

110) Prefrontal, contacts nasal: yes (0); no, excluded by frontal-lacrimal contact (1). (Brusatte et al. 2010:110)

111) Prefrontal, exposure in dorsal view: widely exposed, forms much of orbital rim and usually separates or nearly separates frontal and lacrimal (0); reduced, not exposed along the orbital rim and allows for wide contact between frontal and lacrimal (1). (Carr and Williamson 2010:131; Sereno et al. 2009:20)

112) Prefrontal, ventral process, extent: large, extends more than halfway down the ventral ramus of the lacrimal to make an extensive contribution to the preorbital bar (0); reduced or absent, ventral process is a thin flange that is continuous with the crista cranii of the frontal, and does not extend more than approximately ¼ of the length of the preorbital bar (1). (Carr and Williamson 2010:132)

Frontal

113) Frontal, shape: triangular (0); posterior end expanded into a rectangular shape, with a small anterior triangle (1). (Sereno et al. 2009:38)

114) Frontal, size of single frontal: ratio of anteroposterior length of exposed portion on skull roof to mediolateral width at midpoint: greater than 2.5 (0); less than 2.0 (1). (Brusatte et al. 2010:114)

115) Frontal, supratemporal fossa, anteroposterior length compared to overall length of exposed portion of frontal on skull roof: less than 30% (0); between 30-50% (1); between 50-60% (2); greater than 60% (3). ORDERED (Carr and Williamson 2010:136-137)

116) Frontal, supratemporal fossa, medial extension: fossa restricted to posterolateral corner of frontal (0); meets opposing fossa at the midline (1). (Sereno et al. 2009:39).

117) Frontal, sagittal crest: absent or subtle, only discernable as a slight midline bulge (0); present and pronounced (dorsoventrally tall), single structure (1); present and pronounced (dorsoventrally tall), paired structure (2). UNORDERED (Carr and Williamson 2010:139). Note: The absent and subtle conditions are pooled into a single character state, because in poorly preserved specimens a subtle crest can easily be mistaken for the absence of a crest.

118) Frontal, sagittal crest: anteroposterior length: absent or subtle and short, less than 15% length of the frontal (0); extensive, approximately 25% of the length of the frontal (1). (Carr and Williamson 2010:141)

119) Frontal, postorbital suture: dorsoventrally shallow and undifferentiated (0); dorsoventrally shallow (approximately 6 times longer than deep) and differentiated into a vertical region anteriorly and a horizontal region posteriorly (1); dorsoventrally deep (approximately twice as long as deep) and subtly differentiated into vertical and horizontal regions) (2). (Modified by Brusatte et al. 2010:119 from Carr and Williamson 2010:145; Sereno et al. 2009:37). ORDERED

120) Frontal, contribution to orbital rim: extensive (0); present but limited to a small notch (1); excluded by postorbital-lacrimal contact in large specimens (2). ORDERED (Carr and Williamson 2010:104; Sereno et al. 2009:21) This character has been modified by including state 3 of Brusatte et al. (2010:120), which is now included in a separate character referring to the presence/absence of palpebral ossifications over the orbit).

Parietal

121) Parietal-frontal suture, form: transversely smooth (0); tab-like wedge from parietal extends anteriorly to overlie frontal on midline (1). (Carr and Williamson 2010:147)

122) Parietal, sagittal crest: absent (0); present (1). (Carr and Williamson 2010:148)

123) Parietal, sagittal crest, form: comprised of two parallel crests (0); comprised of a single midline crest (1). (Carr and Williamson 2010:150)

124) Parietal, skull table between supratemporal fossae, width: broad, 10-30% of the mediolateral width of the fossa (0); extremely reduced, sagittal crest or crests pinched between opposing fossae (1). (Sereno et al. 2009:40)

125) Parietal, sagittal crest, dorsoventral depth: consistent across length of crest (0); peaked anteriorly at frontal-parietal suture (1). (Carr and Williamson 2010:146)

126) Parietal, nuchal crest, dorsoventral depth: as low as or lower (0) or extends higher (1) than the dorsal surface of the interorbital region. (Carr and Williamson 2010:149; Sereno et al. 2009:41)

127) Parietals, fusion: unfused (0); fused on the midline in sub-adults and adults (1). (Brusatte et al. 2010:127)

Vomer

128) Vomer, shape of anterior end: lanceolate (lateral margins parallel-sided) (0); expanded into a diamond (1). (Carr and Williamson 2010:176; Sereno et al. 2009:45).

Ectopterygoid

129) Ectopterygoid, extent of internal recess: does not (0) or does (1) inflate the body of the bone and the pterygoid process. (Carr and Williamson 2010:178; Sereno et al. 2009:44)

130) Ectopterygoid, jugal process, external pneumatic foramina leading into jugal recess: absent (0); present (1). (Carr and Williamson 2010:180)

131) Ectopterygoid, jugal process, is not (0) or is (1) inflated by the internal recess. (Carr and Williamson 2010:181)

132) Ectopterygoid, external opening of pneumatic recess, shape: thin ovoid slot (0); larger, round or triangular (1). (Carr and Williamson 2010:183)

133) Ectopterygoid, surface posteriorly adjacent to external opening of pneumatic recess, form: flat, recess grades smoothly into the floor of the lateral temporal fenestra (=subtemporal fenestra) (0); lip, recess separated from lateral temporal fenestra (=subtemporal fenestra) (1). (Carr and Williamson 2010:185)

Palatine

134) Palatine, vomeropterygoid process, ratio of anteroposterior length of dorsal margin to length of greatest constriction of process neck: greater (0) or less than (1) 2.0. (Modified by Brusatte et al. 2010:134 from Carr and Williamson 2010:188)

135) Palatine, vomeropterygoid process, orientation of neck: inclined anterodorsally (0); vertical (1). (Carr and Williamson 2010:189)

136) Palatine, pneumaticity: absent (0); present (1). (Carr and Williamson 2010: 190)

137) Palatine, pneumatic recess, number of external pneumatic openings: one (0); two (1). (Carr and Williamson 2010: 190) Note that some scores are modified here following Gold et al. (2013).

138) Palatine, primary external opening of palatine recess, location of posterior margin: level with or extends posterior to (0) or located far anterior to (1) posterior margin of the vomeropterygoid process neck. (Carr and Williamson 2010:194)

139) Palatine, primary opening of palatine recess, location of anterior margin: level with or posterior to (0) or anterior to (1) anterior margin of the vomeropterygoid process neck. (Carr and Williamson 2010:195)

140) Palatine, jugal process, location of contact surface for lacrimal: posterior ("distal"), separated from opening of palatine recess by wide margin (0); anterior ("proximal"), closely approaches opening of palatine recess (1). (Carr and Williamson 2010:196)

141) Palatine, maxillary process, form of maxillary articulation: flat (0); deeply excavated as a slot, demarcated dorsally by a pronounced lip of bone (1). (Carr and Williamson 2010:197)

142) Palatine, extension of pneumatic recess into jugal process: no (0); yes, process visibly inflated (1). (Carr and Williamson 2009:199)

143) Palatine, maxillary articulation, form: maxilla abuts lateral surface of maxillary process and anterior region of jugal process (0); contact reinforced by a "brace" at the anteroventral corner of the jugal process, which sits within internal antorbital fossa (1). Brusatte et al. (2010:143)

144) Palatine, morphology of maxillary articulation brace: projects ventrally due to a jugal process that extends further ventrally than the maxillary process, such that there is a discrete corner between the two processes in lateral view (0); projects laterally, with no discrete corner between the smoothly confluent jugal and maxillary processes in lateral view (1). Brusatte et al. (2010:144)

Palatal Openings

145) Internal choana, shape: anteroposteriorly elongate oval (0); nearly circular (1). Brusatte et al. (2010:145)

146) Suborbital fenestra, shape: anteroposteriorly elongate oval (0); nearly circular (1). Brusatte et al. (2010:146)

Braincase

147) Braincase, orientation of occipital surface: faces posteriorly (0) or posteroventrally (1). (Sereno et al. 2009:46)

148) Supraoccipital, contribution to dorsal rim of foramen magnum: forms entire rim (0); makes limited contribution to rim via triangular ventral process (1); completely excluded from rim (2). UNORDERED (Modified by Brusatte et al. 2010:148 from Carr and Williamson 2010:158)

149) Supraoccipital, form of dorsal margin: smoothly convex and undivided (0); divided into two processes ("forked") (1). (Carr and Williamson 2010:172)

150) Exoccipital-opisthotic, paroccipital process, ventral flange at distal end: absent (0); present (1). (Brusatte et al. 2010:150)

151) Exoccipital-opisthotic, paroccipital processes, deep fossa on posterior surface dorsolateral to the foramen magnum: present (0); absent (1). (Brusatte et al. 2010:151)

152) Exoccipital-opisthotic, crista tuberalis (=metotic strut), extent in posterior view: limited, mediolateral width across opposing cristae less than one half the dorsoventral depth of the braincase from the dorsal tip of the supraoccipital to the ventral tip of the basal tubera (0); extensive, width greater than one half the braincase depth (1). (Brusatte et al. 2010:152)

153) Exoccipital-opisthotic, fossa for cranial nerves X-XII: shallow (0); deep (1). (Brusatte et al. 2010:153)

154) Basioccipital, basal tubera, dorsoventral depth: less than (0) or greater than (1) depth of occipital condyle. (Brusatte et al. 2010:154)

155) Basioccipital, basal tubera, concave notch ventrally between opposing tubera, dorsoventral depth: shallow, less than 40% depth of tubera (0); deep, approximately 50% depth of tubera (1). Depth of tubera is defined as the entire dorsoventral depth of the basioccipital below the occipital condyle. (Brusatte et al. 2010:155)

156) Basioccipital, subcondylar recess, depth of pneumatic fossae on posterior surface of basal tubera: absent or shallow (0); deep (1). (Carr and Williamson 2010:168)

157) Basisphenoid, basipterygoid recess (pneumatic recess above basipterygoid processes on lateral surface of braincase), absent or present as shallow pneumatic fossa (0); present as a large rectangular fenestra (1). (Carr and Williamson 2010:160)

158) Basisphenoid, basisphenoid recess, orientation of central axis: vertical, recess obscured in posterior view (0); posteroventral, recess partially visible in posterior view (1); extremely posteroventral, recess compressed anteroposteriorly and widely visible in posterior view, and basipterygoid processes located beneath the basal tubera (2). ORDERED (Modified by Brusatte et al. 2010:158 from Carr and Williamson 2010:163,165; Sereno et al. 2009:50)

159) Basisphenoid, basisphenoid recess, inflation of the ceiling of the recess: absent (0); present (1). (Carr and Williamson 2010:166)

160) Basisphenoid recess, shape in ventral view: funnel-like, expands in mediolateral width posteriorly (0); ovoid or circular, no posterior expansion (1). (Brusatte et al. 2010:160)

161) Basisphenoid, shape of basicranium (rectangle defined by positions of both basal tubera and both basipterygoid processes): anteroposteriorly longer than mediolaterally wide (0); wider than long (1). (Carr and Williamson 2010:169; Sereno et al. 2009:49; Li et al. 2010: 272)

162) Parasphenoid, shape of rostrum: anteroposteriorly expanded, ventral margin is a smooth concave arch (0); dorsoventrally expanded, ventral margin is nearly vertical posteriorly and then abruptly transitions to horizontal trend anteriorly (1). (Brusatte et al. 2010:162)

163) Prootic, dorsal tympanic recess: present (0); absent (1). (Brusatte et al. 2010:163)

164) Laterosphenoid, antotic crest separating lateral wall of braincase from orbital and temporal spaces: absent or indistinct (0); present and robust and rugose (1). (Carr and Williamson 2010:152, 155).

165) Laterosphenoid, antotic crest, form: single structure (0); bifurcates ventrally (1). (Brusatte et al. 2010:165)

166) Laterosphenoid, fossa on lateral surface that houses head of epipterygoid: absent or shallow (0); present, deep and rugose (1). (Brusatte et al. 2010:166)

167) Ossified sphenethmoid and mesethmoid (when not fossilized, can be inferred by presence of scars on ventral surface of frontal): absent (0); present (1). (Brusatte et al. 2010:167)

Lower Jaw

168) Mandibular ramus, dorsoventral depth of dentary at level of dentary-surangular contact on the dorsal margin of the lower jaw: less (0) or greater than (1) 18% of the total anteroposterior length of the lower jaw (1). (Carr and Williamson 2010:200)

169) External mandibular fenestra, dorsoventral depth relative to depth of mandible at midpoint of fenestra: greater (0) or less than (1) 25%. (Carr and Williamson 2010:217; Sereno et al. 2009:51)

170) Lower jaw, articulation, glenoid position relative to level of alveolar margin of dentary: level or ventral (0); dorsal (1). (Sereno et al. 2009:54)

Dentary

171) Dentary, position of the transition point between the ventral and anterior margins of the bone in lateral view: below alveoli 1-3 (0); below alveolus 4 (1). (Carr and Williamson 2010:215). Note: We have modified the language of the original Brusatte et al. (2010:171) character, so that this character now refers only to the position of the transition point, whereas a new character (below) refers to the shape of the anteroventral margin of the dentary.

172) Dentary, ventrally projecting rugose process (“chin”) where the anterior and ventral margins of the dentary meet: absent (0); present, visible as a pointed projection in lateral view and convex in medial view, braces dentary symphysis (1). (Brusatte et al. 2010:172)

173) Dentary, symphysis, texture: generally smooth (0); strongly rugose and beveled, with interlocking ridges and convexities for articulation with the opposing symphysis (1). (Brusatte et al. 2010:173)

174) Dentary, articular surface for splenial along ventral region of dentary ramus below the Meckelian fossa, form: dorsoventrally shallow and smooth (0); dorsoventrally deep (nearly as deep as anterior depth of fossa) and rugose (1). (Brusatte et al. 2010:174)

175) Dentary, anterior alveoli, size in comparison to alveoli in middle of tooth row: approximately same size (0); first two alveoli substantially smaller (1); first alveolus substantially smaller (2). UNORDERED. (Brusatte et al. 2010:175)

176) Dentary, primary neurovascular foramina, arrangement: distinct foramina or set into a shallow groove posteriorly (0); set into a deep and sharp groove across the middle and posterior regions of the dentary (1). (Brusatte et al. 2010:176)

177) Dentary, dorsal margin of bone in lateral view, profile: straight (0); strongly concave (1). (Brusatte et al. 2010:177)

178) Dentary, Meckelian groove, form: dorsoventrally deep and shallowly inset into medial surface of bone (0); dorsoventrally shallow and deeply inset into bone, groove appears as a thin, sharp structure (1). (Brusatte et al. 2010:178)

Surangular

179) Surangular, posterior foramen, size: absent or small foramen (0); large fenestra, approximately 30% depth of posterior end of the surangular (1). (Carr and Williamson 2010:204; Sereno et al. 2009:52)

180) Surangular, surangular shelf on lateral surface, form: low ridge (0); prominent ridge that is offset laterally from the bone but dorsoventrally thin (1); prominent shelf that is dorsoventrally deep (2). ORDERED (Carr and Williamson 2010:209; Sereno et al. 2009:53)

181) Surangular, surangular shelf, position and form: placed far dorsal to posterior surangular foramen (0); foramen abuts shelf but shelf projects laterally and does not overhang foramen (1); shelf projects ventrolaterally to overhang foramen (2). ORDERED (Carr and Williamson 2010:208)

182) Surangular, surangular shelf, orientation relative to the long axis of the lower jaw: anterodorsal (0); anteroventral (1); straight anteroposteriorly (2). UNORDERED (Brusatte et al. 2010:182)

183) Surangular, pneumatic fossa posterodorsal to posterior surangular foramen: absent (0); present (1). (Brusatte et al. 2010:183). Note that the character is slightly modified here. Brusatte et al. (2010:183) recognized two conditions of 'present' (shallow vs. deep) which formed an ordered transformational sequence. As discussed by Gold et al. (2013), there is no clear variation in depth of the fossa across tyrannosauroids, so the character is here simplified to a simple presence/absence character.

184) Surangular, adductor muscle attachment site dorsal to surangular shelf, orientation: faces primarily dorsally (0); faces almost equally dorsally and laterally (1); faces primarily laterally (2). ORDERED (Brusatte et al. 2010:184)

185) Surangular, triangular fossa on the lateral surface of the surangular shelf immediately anteroventral to glenoid: absent (0); present (1). (Brusatte et al. 2010:185)

186) Surangular, fossa on the lateral surface of the bone immediately ventral to, and separated from, the glenoid: absent (0); present (1). (Brusatte et al. 2010:186)

187) Surangular, anteroposterior length of anterior flange (region anterior to anterior margin of external mandibular fenestra) compared to overall length of surangular: less than (0) or greater than (1) 30%. (Brusatte et al. 2010:187)

Angular

188) Angular, ventral margin, form: smoothly convex (0); anterior region "flexed" relative to posterior region, such that there is a discrete step between them (1). (Brusatte et al. 2010:188)

Articular

189) Articular, mediolateral width of jaw muscle attachment site: less than (0) or equal to or greater than (1) width of glenoid for articulation with quadrate. (Brusatte et al. 2010:189, inspired by Rauhut et al. 2010).

190) Articular, wide smooth non-articular region between glenoid and attachment site for depressor mandibular muscles: present (0); absent (1). (Brusatte et al. 2010:190, inspired by Rauhut et al. 2010). Note: We have slightly modified this character so that it refers to the presence/absence of a wide non-articular region between the glenoid and jaw muscle attachment surface, as in derived tyrannosauroids there is a very narrow, smooth groove between the two. These taxa were previously scored as lacking the non-articular region, but what they properly lack is a wide non-articular region.

Splenial

191) Splenial, anterior mylohyoid foramen, shape and size: small circular or ovoid opening (0); large, anteroposteriorly ovoid shape (1); extremely large, approximately as deep dorsoventrally

as the anterior process of the splenial (2). ORDERED (Carr and Williamson 2010:210).

192) Splenial, dorsal region overlapped medially by prearticular: absent (0); present (1). (Brusatte et al. 2010:192)

Prearticular

193) Prearticular, ventral bar, series of ridges on lateral surface to strengthen articulation with angular: absent (0); present (1). (Brusatte et al. 2010:193)

Supradentary and Coronoid

194) Supradentary ossification, shape: elongate, shallow strip (0); deep, crescentic shape (1). (Brusatte et al. 2010:194)

195) Supradentary and coronoid ossifications, form of contact at their zone of fusion: smoothly confluent (0); offset by a concave notch (1). (Brusatte et al. 2010:195)

Dentition

196) Premaxillary teeth, position of mesial carina: offset mesial to distal carina on all teeth (teeth ziphodont) (0); rotated distally on premaxillary teeth 1 and 2 (anterior teeth "D-shaped" and posterior teeth ziphodont) (1); rotated distally on all teeth (2). (Modified by Brusatte et al. 2010:196 from Carr and Williamson 2010:219; Sereno et al. 2009:56-58). ORDERED

197) Premaxillary tooth crown 4, apicobasal height relative to largest maxillary crown: subequal (0); approximately 50% (1). (Sereno et al. 2009:59)

198) Premaxillary teeth, median vertical ridge on lingual surface: absent (0); present as subtle structure in anterior (mesial) premaxillary teeth (1); present as pronounced structure in all premaxillary teeth (2). ORDERED (Sereno et al. 2009:60)

199) Premaxillary teeth, curvature of distal (posterior) teeth: recurved (0); straight (1). (Brusatte et al. 2010:199)

200) Maxillary teeth, number: 13 or more (0); less than 13 in the largest adult specimens (1). (Carr and Williamson 2010:220; Sereno et al. 2009:61)

201) Maxillary and dentary teeth, form: ziphodont, transverse width of base less than 60% of mesiodistal length (0); incrassate, width greater than 60% of length (1); incrassate, width nearly equal to length (2). ORDERED (Sereno et al. 2009:62)

Cervical Vertebrae

202) Axis and postaxial cervicals, anteroposterior length of centrum compared to dorsoventral height of posterior centrum face: greater (0); less than or equal to (1). (Brusatte et al. 2010:202, based on Holtz 2001)

203) Axis, pneumatic foramen (pleurocoel), position: near midheight of centrum (0); dorsally located, directly underneath neurocentral suture and directly posterior to diapophysis (1). (Brusatte et al. 2010:203)

204) Axis, pneumatic foramen, extent of surrounding fossa: limited to margins of foramen (0); extensive, occupies most of lateral surface of centrum (1). (Brusatte et al. 2010:204)

205) Axis, ridge on ventral surface of axis: absent (0); present (1). (Brusatte et al. 2010:205)

206) Axis, pneumatic foramina and fossae on each side of the anterior ridge on the neural spine: absent (0); present (1). (Brusatte et al. 2010:206)

207) Axis, neural spine, texture of dorsal region of anterior surface: generally smooth or with subtle texture (0); highly rugose, with series of grooves, ridges, and eminences (1). (Brusatte et al. 2010:207)

208) Axis, dorsal region of neural spine, number of projections on "crown" region: two lateral projections, dorsal surface of spine smoothly concave (0); two lateral projections and one dorsal projection on the midline (1). (Brusatte et al. 2010:208)

209) Axis, supradiapohyseal fossa (fossa posterodorsal to diapophysis): absent or shallow (0); deeply excavated and funnel-like (1). (Brusatte et al. 2010:209)

210) Axis and anterior-middle postaxial cervicals, epipophysis, form: small, pyramidal mound that extends only slightly posterior to postzygapophysis (0); large, rugose flange that extends far posterior to postzygapophysis (1). (Brusatte et al. 2010:210)

211) Cervical vertebrae, neural spines in middle-posterior cervicals, shape: elongate rectangle, much longer anteroposteriorly than wide transversely (0); stout rectangle, base is essentially square shaped due to nearly equal anteroposterior and transverse dimensions (1). (Brusatte et al. 2010:211)

212) Cervical vertebrae, neural spines in middle-posterior cervicals, dorsoventral height: substantially shorter (0); or approximately the same length as or longer (1) than posterior centrum face. (Brusatte et al. 2010:212, based on Holtz 2001).

213) Cervical vertebrae, morphology of posterior centrodiapophyseal laminae in anterior-middle cervicals: absent or present as a weak ridge (0); present as a thick, laterally offset lamina that demarcates a deep infradiapophyseal fossa anteriorly (1). (Brusatte et al. 2010:213)

214) Cervical vertebrae, hypapophysis on anterior region of ventral surface: absent (0); present (1). (Brusatte et al. 2010:214)

215) Cervical vertebrae, position of prezygapophysis in middle cervicals: slightly overhangs centrum laterally (0); strongly overhangs centrum laterally, entire prezygapophyseal facet placed lateral to centrum (1). (Brusatte et al. 2010:215)

216) Cervical vertebrae, orientation of posterior centrodiapophyseal lamina in anterior-middle cervicals: projects posteroventrally, infrapostzygapophyseal fossa located primarily posterior to lamina (0); nearly horizontal, fossa located primarily dorsal to lamina (1). (Brusatte et al. 2010:216)

Dorsal Vertebrae

217) Cervical and dorsal vertebrae, rugose ligament attachment scars in pre- and postspinal fossae: absent or weakly developed (0); present as prominent, rectangular flanges that extend outside of the fossae and are visible in posterior view, but on the dorsal vertebrae only (1); prominent in dorsals and cervicals (2). ORDERED (Brusatte et al. 2010:217)

218) Dorsal vertebrae, extent of pneumaticity, presence of pneumatic foramina on lateral centrum surface: absent or limited to anterior dorsals (0); present throughout dorsal column (1). (Carr and Williamson 2010:226)

219) Dorsal vertebrae, neural spine, level of posterior termination: at approximately the same level as the posterior centrum face (0); far posterior to the posterior centrum face (1). (Carr and Williamson 2010:227)

220) Dorsal vertebrae, anteroposterior length of middle-posterior dorsal centra compared to dorsoventral height of posterior centrum face: greater (0); less than or equal (1). New character, modified from Carr and Williamson 2010: 228)

221) Dorsal vertebrae, middle-posterior dorsals, position of postzygapophysis relative to prezygapophysis: at same level (0); elevated dorsally (1). (Brusatte et al. 2010:221)

222) Dorsal vertebrae, middle-posterior dorsals, form of anterior and posterior centrodiapophyseal laminae: make contact on ventral surface of transverse process, demarcating a triangular infradiapophyseal fossa (0); do not make contact but roughly parallel each other, infraprezygapophyseal and infradiapophyseal fossa merged into a single fossa (1). (Brusatte et al. 2010:222)

Sacral Vertebrae

223) Sacral vertebrae, pneumatic foramina on lateral surfaces of centra: absent (0); present (1). (Carr and Williamson 2010:229)

224) Sacral vertebrae, fenestrae between fused neural spines: neural spines unfused (0); spines fused but fenestrae absent (1); spines fused and fenestrae present (2). ORDERED (Brusatte et al. 2010:224)

225) Sacral ribs, position of central ribs on sacrum: span two sacrals (0); limited to a single sacral (1). (Brusatte et al. 2010:225)

226) Sacral ribs, position of rib attachment for central ribs on individual sacrals: span centrum and neural arch (0); limited to neural arch only (1). (Brusatte et al. 2010:226)

227) Sacral vertebra five, position of ventral margin of posterior articular face in lateral view: at same level as (0) or positioned ventral to (1) ventral margin of anterior articular face. (Brusatte et al. 2010:227)

228) Sacral vertebra five, form of hyposphene: absent or present as a single midline structure (0); present and comprised of two parallel-sided sheets (1). (Brusatte et al. 2010:228)

Caudal Vertebrae

229) Caudal vertebrae, anterior caudals, position of base of neural spine: anterior to (0) or level with or posterior to (1) posterior surface of centrum. (Brusatte et al. 2010:229)

230) Caudal vertebrae, anterior caudals, shape of transverse processes in dorsal view: rectangular, with parallel anterior and posterior sides, or slightly ovoid with a gradual expansion in width distally (0); distal end expanded into a spatulate bulb (1). (Brusatte et al. 2010:230)

231) Caudal vertebrae, anterior caudals, two laminae linking prezygapophysis and transverse process, between which is a deep, triangular fossa: absent (0); present (1). (Brusatte et al. 2010:231)

Scapula

232) Scapula, angle between posterior margin of glenoid and dorsal margin of blade: greater (0) or approximately (1) 90 degrees. (Carr and Williamson 2010:234; Sereno et al. 2009:68).

233) Scapula, acromion, dorsoventral depth: less (0) or greater (1) than 3.0 times minimum dorsoventral depth of blade. (Sereno et al. 2009:67).

234) Scapula, ratio of anteroposterior length of bone to minimum dorsoventral depth of blade: less (0) or greater (1) than 10.0. (Sereno et al. 2009:69)

235) Scapula, ratio of dorsoventral depth of distal expansion to minimum dorsoventral depth of blade: less (0) or greater (1) than 2.5. (Sereno et al. 2009:70)

236) Scapula and coracoid, glenoid, position relative to posteroventral margin of blade: offset posteroventrally (approximately by the width of the neck of the blade) (0); offset only slightly posteroventrally (less than 50% the width of the neck of the blade) (1). (Sereno et al. 2009:66)

Coracoid

237) Coracoid, anteroposterior length at midpoint: approximately 100-150% (0) or 200% or greater (1) than the length of the scapular acromion at midheight. (Sereno et al. 2009:71)

238) Coracoid, coracoid foramen: present (0); absent or extremely small (1). (Brusatte et al. 2010:238)

Humerus

239) Humerus, length relative to the femur: 50-70% (0); 40-30% (1); 25-30% (2). ORDERED (Modified by Brusatte et al. 2010:239 from Carr and Williamson 2010:235; Sereno et al. 2009:72). Note: In cases where the femur is unknown, tibial length or length of the scapular blade can be used as a rough proxy.

240) Humerus, head, form: low, poorly differentiated, crescentic shaped in proximal view, overhangs posterior surface and does not overhang anterior surface (0); enlarged, occupies the majority of the proximal end, bulbous in proximal view, overhangs both posterior and anterior surfaces (1). (Sereno et al. 2009:73).

241) Humerus, apex of deltopectoral crest, location from proximal end: 40-50% (0); 25-35% (1); less than 25% (2) of the length of the humerus. ORDERED (Modified by Brusatte et al. 2010:241 from Sereno et al. 2009:74)

242) Humerus, rotation along shaft, orientation of long axis of proximal end relative to that of distal end: approximately 30-45 degree angle (0); approximately parallel, shaft rotation absent (1). (Carr and Williamson 2010: 236)

243) Humerus, additional muscle attachment tubera at the corner of the anterior and lateral surfaces distal to the deltopectoral crest: absent (0); present (1). (Brusatte et al. 2010:243)

244) Humerus, concave notch between external tuberosity and deltopectoral crest: present, two structures clearly separated (0); absent, two structures smoothly confluenet (1). (Brusatte et al. 2010:244)

245) Humerus, form of distal condyles: lateral and medial condyles expanded equally (offset from shaft in anterior or posterior view is equal) (0); medial condyle expanded further medially than the lateral condyle is laterally (1). (Brusatte et al. 2010:245)

Ulna

246) Ulna, shaft axis, form: bowed (0); straight (1). (Sereno et al. 2009:75)

247) Ulna, distal articular surface, form: convex (0); flat (1). (Sereno et al. 2009:76)

Carpus and Manus

248) Principal distal carpal, shape: semilunate in lateral view with trochlear proximal surface (0); discoid with flat proximal surface (1). (Carr and Williamson 2010:238; Li et al. 2010:276).

249) Manus, number of functional digits: three or more (0); two, metacarpal III is absent or rudimentary, without phalanges (1). (Carr and Williamson 2010:239; Sereno et al. 2009:82)

250) Metacarpal I, medial distal condyle, form: well-formed and large (0); rudimentary (1). (Sereno et al. 2009:78)

251) Metacarpal I, medial margin, shape in proximal view: concave (0); smooth convexity (1). (Brusatte et al. 2010:251)

252) Metacarpals, ratio of the length of metacarpal II to length of metacarpal I: 2-1.8 (0); 1.8-1.6 (1). (Sereno et al. 2009:79)

253) Metacarpals, metacarpal II, mediolateral width at midpoint: equal to or narrower than (0); or more robust than (1) metacarpal I. (Carr and Williamson 2010:240)

254) Manual phalanx II-1, length: longer (0); or subequal to (1) the length of metacarpal 1. (Sereno et al. 2009:81)

255) Manual unguals, flexor tubercle, form: large, robust, rugose, conical structure (0); reduced to a small convexity (1). (Brusatte et al. 2010:255)

256) Manual unguals, degree of recurvature: extensive, flexor margin deeply concave (0); reduced, flexor margin shallowly concave (1). (Brusatte et al. 2010:256)

Ilium

257) Ilium, anteroposterior length compared to length of femur: 70-85% (0); 95-105% (1); 105-115% (2). ORDERED. (Carr and Williamson 2010:251; Sereno et al. 2009:83)

258) Ilium, linear ridge dorsal to the acetabulum on the lateral surface of the blade: absent or low convexity (0); present (1). (Carr and Williamson 2010:246-248; Sereno et al. 2009:84) Note this character is modified from Brusatte et al. (2010:258), which is now separated into two characters: one regarding the presence/absence (here) and the other the orientation of the ridge (character 324).

259) Ilium, preacetabular process, anteroventral corner, form: subtriangular (0); subquadrate with recurved anterior margin, projecting farther anteriorly than remainder of anterior end (“hook” present) (1). (Carr and Williamson 2010:249; Sereno et al. 2009:87)

260) Ilium, dorsal margin of blade, position relative to sacral neural spines: separated by a gap (0); lies against neural spines and opposing iliac blades may make contact above neural spines in some individuals (1). (Sereno et al. 2009:85)

261) Ilium, acetabular antitrochanter at posterior end of supraacetabular shelf: absent or limited in extent (0); present and visible in lateral view as an extensive flange, which is deeply inset from the remainder of the ischial peduncle (1). (Sereno et al. 2009:90)

262) Ilium, acetabular crest, maximum lateral projection relative to ischial peduncle: significantly greater (0); subequal (1). (Sereno et al. 2009:91)

263) Ilium, acetabular crest, extent on pubic peduncle: extensive, extends along most or all of the edge of the peduncle (0); limited, discretely offset from acetabular edge of pubic peduncle (1). (Li et al. 2010:278)

264) Ilium, pubic and ischial peduncles, anteroposterior lengths at dorsal base: pubic peduncle much larger than ischial peduncle (0); both peduncles approximately the same length (1). New character, inspired by Li et al. (2010: 292) but with different scores.

265) Ilium, ventral margin of postacetabular process, shape: straight to slightly convex (0); highly convex, forming a discrete "lobe"-like flange (1). (Brusatte et al. 2010:265)

266) Ilium, dorsal margin, shape: smoothly convex or flat across entire length (0); convex anteriorly and straightens out posteriorly (1). (Brusatte et al. 2010:266)

267) Ilium, postacetabular process, form of posterior margin: tapering, posterior margin convex (0); squared-off, posterior margin vertical (1). (Brusatte et al. 2010:267)

268) Ilium, outline of blade in lateral view: sub-rectangular shape, ratio of anteroposterior length to dorsoventral depth above acetabulum: greater than 3.00, ilium is long and low (0); semi-ovoid shape, less than 2.8 (1). (Brusatte et al. 2010:268) Note that Brusatte and Benson (2013:312) is very similar to this character, and scored essentially identically. We remove the Brusatte and Benson character from this dataset, and slightly modify the language in this character relative to Brusatte et al. (2010:268).

Pubis

269) Pubis, shaft, anterior margin: straight or convex (bowed anteriorly) (0); concave (bowed posteriorly (1). (Carr and Williamson 2010:252)

270) Pubis, pubic tubercle: absent (0); present as a convexity on the anterior margin of the pubis (1); present as a rugose flange that is discretely offset from the anterior margin of the pubis and is bordered posteriorly by heavy rugosities on the lateral surface on the obturator region of the pubis (2). ORDERED (Modified by Brusatte et al. 2010:270 from Carr and Williamson 2010:255)

271) Pubis, pubic tubercle, position: distally positioned, located ventral to the level of the obturator notch (0); proximally positioned, located level with or dorsal to the obturator notch (1). (Brusatte et al. 2010:271)

272) Pubis, pubic boot, anteroposterior length relative to total long axis length of pubis: less than (0) or greater than (1) 60%. (Brusatte et al. 2010:272)

273) Pubis, pubic boot, anterior ramus, length relative to posterior ramus: 10-40% (0); subequal (1). (Carr and Williamson 2010:256-258; Sereno et al. 2009:97).

274) Pubis, pubic boot, position of anterior process relative to posterior process: displaced dorsally, resulting in a highly convex ventral margin of the boot (0); placed at the same level, ventral margin of the boot essentially straight (1). (Carr and Williamson 2010:259)

275) Pubis, orientation of shaft relative to long axis of ilium when pelvis is in articulation: perpendicular, shaft approximately vertical (0); oblique, shaft is propubic (1). (Brusatte et al. 2010:275)

276) Pubis, anteroposterior expansion of proximal obturator plate region relative to the anterior edge of the pubis shaft at its midpoint along the long axis of the shaft: less than (0) or greater than (1) twice the anteroposterior thickness of the shaft at its midpoint. (Brusatte et al. 2010:276)

277) Pubis, obturator notch, form: discrete structure, demarcated ventrally by extensive obturator flange (0); essentially absent, no ventral flange (1). (Brusatte et al. 2010:277)

Ischium

278) Ischium, ischial tubercle ventral to iliac peduncle: absent or present as a groove (0); present as a convex bulge on the posterior surface of the ischium (1); present as a rugose, ovoid or triangular flange whose lateral surface is depressed relative to the remainder of the ischium (2). ORDERED (Carr and Williamson 2010:261; Sereno et al. 2009:93)

279) Ischium, distal end, expansion relative to midshaft: expanded, “foot” present (0); tapering, “foot” absent (1). (Carr and Williamson 2010:264; Sereno et al. 2009:95)

280) Ischium, midshaft diameter (anteroposterior) relative to pubic midshaft diameter: 60-100% or ischium is greater (0); 30-50% (1). (Carr and Williamson 2010:265; Sereno et al. 2009:94)

281) Ischium, position of apex of obturator flange, distance between apex and proximal end of ischium: approximately 40% of ischium length (“distal flange”) (0); less than 30% of ischum length (“proximal flange”) (1). (Brusatte et al. 2010:281)

282) Ischium, position of medial apron: along posterior margin of shaft (0); along anterior margin of shaft (1). (Brusatte et al. 2010:282)

Femur

283) Femur, oval scar on posterior surface of shaft distal to fourth trochanter, position: absent or very low (0); present, positioned medial to posterior midline of shaft (1); present, located on the posteromedial edge of the shaft (2). ORDERED (Carr and Williamson 2010:268) Note: We have

modified this character by adding a second derived state, which is a special condition of state 1. Therefore, this is an ordered character.

284) Femur, lesser trochanter, height relative to greater trochanter: shorter, terminates further distally (0); subequal or slightly taller, the two structures extend to approximately the same level proximally (1). (Carr and Williamson 2010:269; Sereno et al. 2009:98)

285) Femur, proximal margin in posterior view: approximately straight and perpendicular to the long axis of the shaft (0); approximately straight and oriented at an obtuse angle to the long axis of the shaft (=dorsally or proximally inclined head) (1); concave and oriented at an obtuse angle to the long axis of the shaft, due to a head that is proximally inclined and a greater trochanter that is elevated proximally substantially relative to the central portion of the head (2). ORDERED (Brusatte et al. 2010:285) Note: this character is modified from Brusatte et al. (2010:285), following the usage in Brusatte et al. (2014:711). The character states are better defined and a new state is added.

286) Femur, trochanteric fossa on the posterior surface of the head, lateral to the sulcus for the capital ligament, form: absent or shallow (0); deep fossa (1); deep, extensive triangular depression that covers most of the posterior surface of the femur proximally and is demarcated medially and ventrally by a pronounced, curving, swollen ridge (2). ORDERED (Brusatte et al. 2010:286) Note that the language of this character is modified to correct a mistake in Brusatte et al. (2010:286): the fossa being referred to is the trochanteric fossa, and it is on the posterior surface of the femoral head.

287) Femur, lesser trochanter, accessory trochanter along the anterior margin, form: present as a pronounced flange ventrally, which is distinguishable from the remainder of the lesser trochanter in lateral view (0); reduced to a subtle convexity, barely distinguishable from the remainder of the trochanter (1). (Brusatte et al. 2010:287)

288) Femur, fourth trochanter, position, measurement from proximal margin of head to distal termination of trochanter relative to total length of the femur: 35% or less (0); 40% or greater (1). (Brusatte et al. 2010:288)

289) Femur, lateral condyle, shape in distal view: circular or ovoid (0); ovoid, but with an anterior bulge that is slightly separated from the remainder of the condyle (1). (Brusatte et al. 2010:289)

290) Femur, extensor groove on anterior surface of distal end, form: absent or extremely shallow, anterior surface flat between the condyles in distal view (0); groove present but shallow, expressed as a broad concave anterior margin in distal view but present as an extensive depression on the anterior surface of the femur (1); groove present and deep, expressed as a deep, U-shaped cleft in distal view and present as an extensive depression on the anterior surface of the femur (2). ORDERED (Brusatte et al. 2010:290)

291) Femur, mesiodistal crest, form: single structure (0); bifurcates distally to enclose fossa on the medial surface of the medial condyle (1). (Brusatte et al. 2010:291)

Tibia

292) Tibia, length relative to the femur: 1.05 or greater (0); less than 1.00 (1). (Sereno et al. 2009:99)

293) Tibia, lateral condyle of proximal end, anterior process: absent (0); present (1). (Carr and Williamson 2010:270)

294) Tibia, lateral malleolus, lateral extent: limited, mediolateral measure is less than 40% of mediolateral width of adjacent shaft (0); extensive, mediolateral measure greater than 40% of mediolateral width of adjacent shaft (1). (Carr and Williamson 2010:271)

295) Tibia, lateral malleolus, position relative to medial malleolus: extend to approximately the same level distally (0); lateral malleolus extends significantly further distally than medial malleolus (1). (Brusatte et al. 2010:295)

Fibula

296) Fibula, iliofibularis tubercle, form: single crest (0); large, rugose, and formed by two crests separated by a depressed fossa ("bipartite" condition) (1). (Carr and Williamson 2010:272)

Astragalus

297) Astragalus, ascending process, mediolateral width of base compared to width of entire bone: half or less (0); greater than half (1). (Carr and Williamson 2010:273)

298) Astragalus, fossa on anterior surface of ascending process, form: shallow concavity that covers most of the ventral region of the ascending process (0); deep, triangular or ovoid fossa immediately above midpoint of condyles, set within a broad fossa that covers most of the ventral region of the ascending process (1). (Brusatte et al. 2010:298)

Pes

299) Pes, arctometatarsalian condition, form of shaft of metatarsal III: pes not arctometatarsalian, mt III subcylindrical and its proximal end is exposed anteriorly (0); pes arctometatarsalian, mt III wedge-shaped and its proximal end is covered anteriorly by contact between metatarsals II and IV (1). (Carr and Williamson 2010:276; Sereno et al. 2009:100)

300) Pes, metatarsal III, form of medial surface in anterior or posterior view: straight or subtly convex (0); with medial convex expansion forming a bulge along the distal part of the shaft (1). (Li et al. 2010:289)

301) Pes, metatarsal III, ventral non-articular surface immediately proximal to the distal condyles, form: concave (0); raised subtriangular platform (1). (Sereno et al. 2009:101)

302) Pes, metatarsals II-IV, distal separation when in articulation: metatarsals closely appressed and distance between II-III and III-IV is approximately equal (0); distal ends of II and IV diverge from III, and distance between III-IV greater than that between II-III (1). (Brusatte et al. 2010:302)

303) Pes, metatarsal II, articular scar for metatarsal III on distal portion of lateral surface of shaft, form: subtle (0); enlarged as a rugose fossa that occupies more than half of the proximodistal length of the shaft and expands in anterposterior width distally (1). (Brusatte et al. 2010:303) Note that Loewen et al. (2013:483/484 and 493/494) includes separate characters for this feature on both metatarsal II and IV. However, all taxa that have the rugose fossa on metatarsal II also have it on metatarsal IV. For both metatarsals, this structure is related in part to the overall arctometatarsalian morphology of the metatarsus. Therefore, we consider them redundant and include only a single character.

304) Pes, metatarsal II, lateral surface in proximal view, shape: flat or weakly concave (0); strongly concave (1). (Brusatte et al. 2010:304)

305) Pes, metatarsal IV, distal end, ratio between anteroposterior long axis (measured from midpoint of condyles posteriorly to anterior surface of bone) and mediolateral width (measured at midpoint): greater than 1.40, distal surface is elongate anteroposteriorly (0); between 1.40 and 1.20 (1); less than 1.20, distal surface nearly square-shaped with nearly flat anterior surface (2). ORDERED (Brusatte et al. 2010:305)

306) Pes, proximal pedal phalanges, ratio of length to midshaft width: greater than (0) or less than (1) 3.0. (Brusatte et al. 2010:306)

307) Pes, pedal unguals, lip overhanging proximal articular surface dorsally (on extensor surface): present (0); absent or reduced to a subtle tuber (1). (Brusatte et al. 2010:307)

Characters from Brusatte & Benson (2013)

308) Ilium, linear ridge dorsal to acetabulum on the lateral surface of the blade, dorsal extent: elongate, extends to dorsal margin of iliac blade (0); short, terminates ventral to dorsal margin of iliac blade (1). (Brusatte and Benson 2013:308, based on Benson 2008)

309) Ilium, linear ridge dorsal to acetabulum on the lateral surface of the blade, robustness: thick, anteroposterior width of ridge greater than 20% of dorsoventral length of ridge (0); thin, width less than or equal to 20% of length (1). (Brusatte and Benson 2013:309) Dorsoventral length is measured up until the dorsoventral midpoint of the iliac blade, in order to standardize measurements between taxa that have a dorsoventrally extensive ridge and those that have a shorter ridge that terminates ventral to the dorsal margin of the iliac blade (see character 308).

310) Ilium, brevis fossa, orientation: faces primarily ventrally, but it is widely visible in lateral view (0); faces primarily ventrally and medially, and it is obscured in lateral view (1). (Brusatte and Benson 2013:210)

311) Ilium, mound-like eminence on lateral surface of pubic peduncle: absent (0); present (1). (Brusatte and Benson 2013:311, based on Benson 2008).

Note that Brusatte & Benson (2013:312) is deleted here, as it is considered redundant with Brusatte et al. (2010:268), which is used in this character list above.

312) Ilium, width of notch between preacetabular process and pubic peduncle: wide, widens anteriorly when seen in lateral view (0); narrow, remains narrow across its entire length when seen in lateral view (1). (Brusatte and Benson 2013:313, based on Benson (2008).

Note that Brusatte & Benson (2013:314) is deleted here, as it is considered redundant with Brusatte et al. (2010:221), which is used in this character list above.

Characters from Lu et al. (2014)

313) Nasal, series of pronounced, discrete rugosities on dorsal surface of middle portion of bone, posterior to the external naris: absent (0); present (1). State 1 is present in *Alioramus remotus*, *Alioramus altai*, and *Qianzhousaurus*.

314) Maxillary fenestra, proportions: ratio of maximum anteroposterior length to maximum dorsoventral depth: less than (0) or greater than (1) 1.9. State 1 is present in *Alioramus altai* and *Qianzhousaurus*. It cannot be assessed in *Alioramus remotus*.

315) Dentary teeth, number: 17 or less (0); 18 or more (1). State 1 is present in *Alioramus remotus*, *Alioramus altai*, and *Qianzhousaurus*, as well as *Proceratosaurus* and some outgroup taxa (e.g., compsognathids: *Compsognathus*; ornithomimosaurs: *Pelecanimimus*).

Characters newly added here

316) Lacrimal, cornual process on dorsal surface: absent (0); present (1). (Modified from Brusatte et al. 2010:49, which has been split into two characters. The character referring to differences in the size/shape of the cornual process is retained as character 49 in this dataset, whereas a simple presence/absence character is introduced here.)

317) Quadrate, form of pneumatization, deep recess on the anterior surface where the pterygoid wing and condyles meet: absent (0); present (1). (Modified from Brusatte et al. 2010:106, which has been split into two characters. The character referring to the mere presence/absence of quadrate pneumaticity is retained as character 106 in this dataset, whereas that referring to the presence/absence of a deep funnel-like recess leading into the internal pneumatic cavity is used here).

318) Quadrate, form of pneumatization, large single pneumatic foramen on posterior or posteromedial surface of the shaft: present (0); absent (1). This character can also be considered an extension of Brusatte et al. (2010:106). It refers to a distinct condition in theropods in which there is a pneumatic foramen on the posterior/posteromedial surface of the quadrate leading into the internal recess. This is described in Gold et al. (2013).

319) Supraoccipital, morphology of dorsal end of bone: flat plate that abuts (sits directly behind) the posterior surface of the parietal (0); folded and curves posteriorly to overlie the remainder of the supraoccipital, with a depression at the center of the fold defining left and right ‘tab-like’ processes (1). (Carr and Williamson 2010:172) For a description of this character, see Bever et al. (2013).

320) Nasal, premaxillary processes (=supranarial processes), extent of their apposition to each other on the midline in dorsal view: apposed for nearly their entire length (may abruptly separate from each other at their tips) (0); not apposed for most of their length, and therefore do not abruptly separate from each other at their tips (1).

321) Ilium, linear ridge dorsal to the acetabulum on the lateral surface of the blade, orientation: vertical or anterodorsally oriented (0); posterodorsally oriented (1). (Carr and Williamson 2010:246-248; Sereno et al. 2009:84) Modified from Brusatte et al. 2010:258, which has been split into two characters in this dataset (character 258 and the current character).

322) Tibia, proximal surface, proximal extent of cnemial crest relative to the proximal extent of the posterior condyles: cnemial crest extends further proximally than condyles (0); cnemial crest and condyles extend to the same approximate level proximally (1). (Brusatte et al. 2014:851)

<u>Characters from Loewen et al. (2013)</u>

323) External naris, position of posterior margin: anterior to antorbital fossa (0); posterior to or even with the antorbital fossa, but anterior to the maxillary fenestra (1); posterior to antorbital fossa, and posterior to the anterior border of the maxillary fenestra (2). ORDERED. (Loewen et al. 2013:2). Note: Loewen et al. (2013:2) scored *Dilong* for state 1, but the newly prepared right side of the skull (which is much better preserved than the somewhat crushed left side) shows that this taxon possesses state 2.

324) Orbit, shape: round (0); more than 10% taller dorsoventrally than long anteroposteriorly, but less than twice as tall as long (1); dorsoventrally elongate, more than twice as tall as long (2). ORDERED. (Loewen et al. 2013:4, modified from Li et al. 2010:2)

325) Orbit, size: large, anteroposterior length at least three times the anteroposterior length of the laterotemporal fenestra (0); small, anteroposterior length less than three times the anteroposterior length of the laterotemporal fenestra (1). (Loewen et al. 2013:5) Note: the length of the laterotemporal fenestra and the orbit are taken to be the minimum length measurement of each opening.

326) Orbit, orientation: laterally directed, orbit is directed parallel to the sagittal plane (0); anterolaterally directed, orbit is directly anteriorly at greater than 20 degrees from the sagittal plane (1). (Loewen et al. 2013:6) Note: Loewen et al. (2013:6) included an intermediate state of “sublaterally, orbit is directed from 10 to 15 degrees from the sagittal plane”. We do not include this intermediate state because we do not find that tyrannosauroids can be measured to the precision needed to distinguish a ~10-15 degree orientation from a ~20 degree orientation,

especially given the crushing that many specimens have undergone. We also find that the major distinction between tyrannosauroids is between taxa with an essentially laterally facing orbit and one that has a substantial anterior orientation, and hence decided that a simple binary character is most appropriate.

327) Maxilla, maxillary fenestra, shape of posterior border: rounded (0); angular, coming to a point (1). (Loewen et al 2013:34) Note: Loewen et al. (2013:34) scored *Dilong* for state 1. The fenestra looks to have an angular posterior margin on the right side but this is at least partially caused by damage. The better preserved left maxillary fenestra exhibits a rounded posterior border of the fenestra, and therefore we score *Dilong* for state 0. Loewen et al. (2013:34) also scored *Alioramus altai*, *Albertosaurus*, *Gorgosaurus*, and *Daspletosaurus* for state 0, but these are correctly scored as state 1.

328) Maxilla, small secondary posterior maxillary fenestra posterior to the maxillary fenestra, within the interfenestral strut: absent (0); present (1). (Loewen et al. 2013:37)

329) Maxilla, elongate secondary fossa dorsal to maxillary fenestra inside antorbital fossa on the lateral surface of the maxilla: absent (0); present (1). (Loewen et al. 2013:38)

330) Maxilla, palatal shelf on the medial surface of the bone, shape: straight across its anteroposterior length (0); sigmoid, such that the ventral margin is concave and convex in parts (1). (Modified from Loewen et al. 2013:44) Note: We score several taxa that Loewen et al. (2013:44) scored affirmatively as uncertain, because we cannot observe the palatal shelf in medial view, which is necessary to score this character.

331) Maxilla, depressor pits for dentary teeth in the palatal shelf on the medial surface of the maxilla dorsal to the interdental plates: absent (0); present (1). (Loewen et al. 2013:46) Note: Loewen et al. (2013:46) divided the present condition into two ordered states: shallow and deep pits. We cannot easily recognize a clear distinction between these conditions, and therefore use a simple absence vs. presence character.

332) Lacrimal, cornual process, morphology in anterior view: not inflated (0); inflated, such that it appears swollen (1). (Modified from Loewen et al. 2013:65)

333) Lacrimal, supraorbital process (=posterior process), size and shape: absent or very short (0); present, long and gracile (dorsoventrally shallow) (1); present, long and robust (dorsoventrally tall) (2). ORDERED (Loewen et al. 2013:68)

334) Lacrimal, subocular process restricting anterior orbit margin on posterolateral margin of ventral process, and defining ocular region of orbit: absent (0); present and subtle (1); present and rugose (2). ORDERED (Loewen et al. 2013:81) Note: We have added a second derived state to refer to the especially large and rugose processes of some derived tyrannosaurines.

335) Parietal, frontoparietal peak, presence, lateral view: absent (0), present (1). (Modified from Loewen et al. 2013:100). Note: We have modified this character by simplifying it into a binary character. The original Loewen et al. (2013:100) character is a multistate with several different

conditions referring to progressively larger frontoparietal peaks, defined relative to the height of the nuchal crest. However, we find that the nuchal crest is a poor point of reference, as it varies widely in shape and depth. This is especially problematic for taxa in which the nuchal crest is dorsoventrally short in some, but not all, specimens (such as *Gorgosaurus*). Therefore, we find it simpler and clearer to use a character that simply refers to the presence/absence of the peak, not its difficult-to-quantify height.

336) Frontal, mediolateral cross section ahead of the dorsotemporal fossae in dorsal view: flat (0); dorsally convex, in which the contralateral bones extend dorsomedially to the interfrontal suture (1); concave, in which the bones extend ventromedially to the interfrontal suture (2). UNORDERED (Modified from Loewen et al. 2013:97)

337) Parietal, sagittal crest, ventral extent, lateral view: extends above the interorbital region (0), extends below the interorbital region (1). (Modified from Loewen et al. 2013:102). Note: We have modified the Loewen et al. (2013:102) character into a simpler binary character. The original Loewen et al. character distinguished between a flat parietal and two conditions of a sagittal crest with a concave surface in lateral view: shallow and deep. We find that the major difference among tyrannosauroids is between the generally flat or subtly concave sagittal crest of most species and the very deeply concave crests of some derived tyrannosaurines, which are so concave that the crest extends below the interorbital region in lateral view. Therefore, we use a binary character.

338) Postorbital, anterior process, shape in lateral view: dorsoventrally shallow, anterior tip is less than the 30% of the anteroposterior thickness of the ventral process at midlength (0); dorsoventrally tall, anterior tip is greater than half the thickness of the ventral process (1). (Loewen et al. 2013:105) Note: We have slightly modified this character so that the measurements of each state are specified. This means we have introduced a few scoring changes relative to the Loewen et al. study: *Proceratosaurus* (0 to ?), *Albertosaurus* (1 to ?), and *Teratophoneus* (1 to ?).

339) Separate palpebral ossification between the postorbital and lacrimal above the orbit: absent (0); present (1). (Loewen et al. 2013:116)

340) Squamosal, anterior extent of anterior process: located at or ahead of the anterior margin of the laterotemporal fenestra (0); posterior to the anterior margin of the fenestra (1). (Loewen et al. 2013:117, modified from an earlier character of Carr & Williamson 2010). Note: Loewen et al. (2013:117) scored *Dilong* for state 1, but the newly prepared right side of the skull shows that state 0 is the correct score.

341) Squamosal, pneumaticity, morphology in ventral view: pneumatic recess absent, but instead a fossa is seen in the ceiling of the bone (0); recess is present, in which the edges of the recess are undercut and lead into a pneumatic space that hollows out the bone (1). (Loewen et al. 2013:126, modified from an earlier character of Carr & Williamson 2010) Note that Loewen et al. (2013:126) divide the present state into two conditions: ‘foramen present’ and ‘foramen and fossa present’. We do not know of any tyrannosauroids which have a pneumatic foramen but not a large pneumatic fossa. We find that the major distinction in tyrannosauroids is between an

apneumatic squamosal and one with a large, deep recess on the ventral surface, which penetrates deep into the bone such that the squamosal is cup-like.

342) Jugal, postorbital process, basal anteroposterior length relative to suborbital height: anteroposteriorly short, less than 100% suborbital height (0); anteroposteriorly long, greater than 100% suborbital height (1). (Modified from Loewen et al. 2013:138) Note: We have slightly revised this character so that the reference point is the suborbital height of the jugal, not the total length of the jugal (as used by Loewen et al.), because tyrannosauroid jugals are often broken anteriorly and/or posteriorly. This leads to some scoring differences with Loewen et al.

343) Jugal, ornamentation on the lateral surface: absent (0); rugose thickening present (1); pronounced laterally directed horn or boss (2). ORDERED (Loewen et al. 2013:143) Note: This is a modified version of Loewen et al. (2013:143), in which a second derived state is added to refer to the pronounced lateral horns of alioramins. Our conception of this character differs somewhat from Loewen et al. (2013:143), who scores state 1 as present in all tyrannosauroids except for *Raptorex* and *Xiongguanlong*. We sense that Loewen et al. (2013:143) is conflating two characters: a raised rim on the lateral surface that parallels the ventral margin of the bone (used as character 80 here, which is present in basal tyrannosauroids such as *Guanlong* and *Dilong*) and a rugose thickening on the central part of the lateral surface of the bone (which is seen in derived tyrannosauroids such as *Tyrannosaurus* and *Albertosaurus*). We retain these as separate characters here. We also score *Xiongguanlong* for state 1, contra Loewen et al. (2013:143).

344) Quadratojugal, distinct posterior process at the posteroventral corner of the bone: absent, quadratojugal inverse L-shaped (0); present, quadratojugal the shape of an upside down T (1). (Loewen et al. 2013:153)

345) Quadrate foramen, position of ventral edge of the foramen from the ventral edge of the quadrate: more than twice the dorsoventral depth of the quadrate condyles in posterior view (0); twice the depth of the condyles or less (1). (Modified from Loewen et al. 2013:158 by changing to a slightly different quantitative system) Note: Loewen et al. (2013:158) scores *Eotyrannus* for state 1, but we have rescored as state 0. They consider *Dilong* as possessing state 0 but we cannot confirm this, so score it as ‘?’.

346) Quadrate, fossa on medial surface of pterygoid process (=orbital process): absent (0); shallowly inset (1); deeply excavated (2). ORDERED (Loewen et al. 2013:160) Note: We have some scoring differences relative to Loewen et al. (2013:160): *Alioramus remotus* (1 in Loewen et al., ? here), *Albertosaurus* (1 to 2), *Teratophoneus* (2 to 1), *Lythronax* (2 to ?), *Tyrannosaurus* (0 to 1), *Tarbosaurus* (0 to 1).

347) Supraoccipital, pronounced and strongly demarcated median ridge on posterior occipital surface: absent (0); present (1). (Modified from Loewen et al. 2013:166, using language in Brusatte et al. 2014:253) Note: We have extensively modified this character relative to both Loewen et al. (2013:166) and Brusatte et al. (2014:253). Loewen et al. (2013:166) utilized a multistate character differentiating between a supraoccipital with a flat posterior (occipital) surface and those with either a ridge or groove on this surface. The groove is an epiphenomenon

of the tab-like processes of the supraoccipital, which are encapsulated by another character, character 320 here, so this state is not used for the present character. Therefore, this character distinguishes between the absence or presence of the midline ridge. Brusatte et al. (2014:253) scored all tyrannosauroids as possessing this ridge, but this is incorrect: derived taxa such as *Tyrannosaurus* and *Albertosaurus* do not have a distinct ridge, as they possess the groove. Therefore, scores for these taxa are corrected here. Note that Loewen et al. (2013:166) scored *Guanlong* for a flat supraoccipital (state 0), but it has the marked midline ridge (state 1).

348) Parietal, posterior surface of nuchal crest in those taxa in which the crest extends higher than the dorsal surface of the interorbital region: thin but sharp midline ridge (0); mediolaterally wide triangle (apex point ventrally), which sometimes (but not always) has grooves on each side (1). (Modified from Loewen et al. 2013:167) Note: The original Loewen et al. (2013:167) character refers to the 'median ridge (of the supraoccipital) above (the) dorsal process", but this is unclear because no portion of the supraoccipital extends above the dorsal process of the bone. What we believe this character to represent is differences in the morphology of the parietal nuchal crest, which is the structure that is above the supraoccipital dorsal process. Therefore, we have substantially revised this character to reflect our understanding of anatomical variation in this region.

349) Laterosphenoid, ventrolateral shelf: absent (0); present (1). (Loewen et al. 2013:185, modified from an earlier character of Carr & Williamson 2010) Note: Loewen et al. (2013:185) score the shelf as absent in *Guanlong* and *Dilong* and present in *Teratophoneus*, but we are unable to confirm these scores based on personal observation so we score these taxa as uncertain. Loewen et al. (2013:185) also scores *Bistahieversor* for state 1, but we rescore as state 0 following personal observation of the specimen (TDC).

350) Prearticular, cross section of midshaft, ratio of mediolateral width to dorsoventral height: less than 0.9 (0), 0.9 or greater (1). (Modified from Loewen et al. 2013:281). Note: We have modified the original Loewen et al. (2013:281) multistate character by quantifying it and changing it into a simpler binary character. It is difficult for us to quantitatively distinguish between two of their states 'sub-round' and 'round'. Therefore, we have excluded their 'sub-round' intermediate condition and now use a binary character that is quantitatively defined. This leads to some scoring differences with Loewen et al. (2013:281).

351) Maxillary teeth, carinae, position: symmetrical, with mesial and distal carinae on opposite sides of a lens-shaped crown (0); asymmetrical, with one or both carinae not centered on its side (1). (Modified from Loewen et al. 2013:296). Note: We have extensively modified the character scores of Loewen et al. (2013:259). Loewen et al. score several derived tyrannosauroids for state 0, including *Daspletosaurus*, *Tyrannosaurus*, and *Tarbosaurus*. Based on our observations, state 1 is present in all-scoreable tyrannosaurids, so these taxa are re-scored. Other character re-scorings have to do with changing affirmative scores in Loewen et al. to "?" to reflect uncertainty in specimens we have examined (e.g., *Bistahieversor*).

352) Maxillary and dentary teeth, size of mesial (=anterior) and distal (=posterior) denticles: not substantially different in size (0); mesial denticles, when present, substantially smaller than distal denticles (1). (Modified from Loewen et al. 2013:302, using the language of Brusatte et al.

2014:244) Note: We use the language from Brusatte et al. (2014:244), which means that some taxa are scored differently from Loewen et al. (2013:302). These include: *Kileskus* (0 in Loewen et al., ? here), *Guanlong* (0 vs. 1), *Sinotyrannus* (0 vs. 1), *Dilong* (0 vs. 1), *Eotyrannus* (0 vs. 1), *Xiongguanlong* (1 vs. ?), *Alioramus remotus* (0 vs. ?), *Appalachiosaurus* (0 vs. ?), *Bistahieversor* (0 vs. ?)

353) Axis, neural spine, form in lateral view: sheet-like (0); anteroposteriorly reduced and rod-like (1). (Loewen et al. 2013:310, included in Brusatte et al. 2014:259) Note: We score some taxa differently from Loewen et al. (2013:310): *Guanlong* (0 in Loewen et a. 2013, ? here), *Eotyrannus* (? vs. 0), *Raptorex* (1 vs. ?). We also score *Xiongguanlong* for state 1, contra the state 0 score in Brusatte et al. (2014:259).

354) Ilium, pubic peduncle, orientation of articular facet relative to the dorsal margin of the iliac blade: essentially parallel, such that the articular margin is nearly horizontal when the dorsal margin of the blade is held horizontal (0); obliquely, such that the articular margin faces anteroventrally when the dorsal margin of the blade is held horizontal (1). (Modified from Loewen et al. 2013:416, by including a reference point from which to measure the orientation of the articular surface) Note: Because we have modified the reference point for measuring the orientation of the articular surface, we have several scoring differences with Loewen et al. (2013:416): *Guanlong*, *Juratyrant*, *Stokesosaurus*, and *Sinotyrannus* are all scored 0 by Loewen et al. (2013:416) but 1 here; *Eotyrannus* is scored 0 by Loewen et al. but ? here. We note that some taxa have a subovoid iliac blade, so it is difficult to hold the dorsal margin of the blade horizontal as a reference for scoring this character. In these taxa, the ilium is held in a standard position by placing the apex of the subovoid shape so that it is centered dorsally.

355) Ischium, shaft curvature: roughly straight or slightly curved so that posterior surface is concave (0); curved so that anterior surface is concave (1). (Loewen et al. 2013:436, also used by Li et al. 2010:167)

356) Femur, crista tibiofibularis, size in distal view: large, width of crista larger than half the mediolateral width of the lateral condyle (0); small, width of crista smaller than half the mediolateral width of the lateral condyle, with crista restricted to the medial half of the lateral condyle (1). (Loewen et al. 2013:458)

357) Metatarsal IV, lateral muscle attachment scar, form: absent or dorsoventrally long and narrow (0); wide oval rugosity that covers more than half the shaft (1). (Modified from Loewen et al. 2013:495). Note: We pool ‘absent’ and ‘narrow’ into the same character state, because preservation often makes it difficult to distinguish a narrow scar from one that is truly absent. Our understanding of this character, and our scores, differ somewhat from Loewen et al. (2013:495).

358) Dentary, orientation of anterior margin in lateral view, relative to the dorsal margin of the bone: steep, approaches 90 degrees (0); anterior margin approximately 45 degrees or less (1). (Carr and Williamson 2010:215; Loewen et al. 2013:253).

Characters 359-366 from Carr and Varricchio (in press)

359) Lacrimal, cornual process, height in lateral view: tall, greater than 60% height of antorbital fossa below it (0); short, less than 60% of the height of the fossa below it (1).

360) Lacrimal, accessory posteriorly-extending cornual process on the lateral surface between the cornual process and the supraorbital ramus: absent (0); present (1).

361) Jugal, orbit margin in juveniles, form in lateral view: straight (0); concave (1).

362) Frontal, prefrontolacrimal process, anteroposterior length in dorsal view: absent or short (0); long (1). Note: we pool ‘absent’ and ‘short’ into the same state, because in poorly preserved specimens a short process can easily be mistaken for absent.

363) Frontal, nasal process, ridge that extends anteroposteriorly along the process in dorsal view: absent (0); present (1).

364) Vomer, keel below stem, depth: shallow, present as a low ridge (0); present as a deep keel-like flange (1).

365) Laterosphenoid, mediolaterally oriented ridge on dorsolateral surface that extends laterally to the edge of the dorsotemporal fenestra, visible in dorsal view: absent (0); present (1).

366) Quadratojugal, pneumatic foramen on the lateral surface of the shaft: absent (0); present (1).

3. Phylogenetic Taxon-Character Matrix

The following is the matrix in TNT format. An Excel spreadsheet is also included as supplementary information, in which all scoring changes from previous analyses are denoted in red.

xread
366 32
Allosaurus
0100000000000101000000010000001000110000000000210110101001000010001????000
0000001?0??100?00000?000000000000011000001000000000?0?10000000000????000?0001001
0000000010000000000000000000002000000000000000000000001000000100000020010000[01]00
00100000000001000000000000100101000000000000100?0001010001000000102011000000000000
110??00100010?00?0021000000000100010000000001000001100000?00??0
Maniraptora
00000?000000[01]000000?00100?00?0001110[01]00000100000???000000000?01?00??
?0000000000?0??010?01000?000000001001[01]00????0000000000?0?0?0000??001000?0?0?0
0000[01]000000[01]01000000000000000000000001[01]0000000?000000[01]0000000[01]0[0
1]00001?111100[01]1000?00000000000010000000000000000000000000001100??011000000
0010000000000100000000000??10000000000?1000000000?10000101000010?0?0001000100?????
??0
Ornithomimos
0000001000000?00000?00000?00?0001000000001?0000???00000000??00?0000?0000
000000?0??010?00?00??00000?01000[01]00?000000000?000?0?0000??00001000?????0?0000
0100000000[01]00???000000???0000000000000000???000000000?00?000000000000100[12]
00000000000000000001000000[01]000[01]0101000100100[01]0000?0[01]00011001100000000000
0000100100?0000??00001100000?10000000???2100?10001000?1??0??0100?1???????0
Compsogs
?000000000000000000?00100?00?0?0000?100000??0200???00000?00??00?0???00000
0000010??010?10??0??0000?000???1???100000000?000?0??0??????00?????????0????????????
??????????0?000000000000000???0???????0000[01]00???????00000??00000?000?0?0??00000
10000?0??00100?[01]0000000[01]0?00000000?00?0010000??00?00???0??00?000?0???00??0?
000[01]0?0?0?11000000?0?010??10??000?????00?010??[01]???????0
Kileskus
???1??110111200100???0?00?0000?0000?0??
???
??????????????0100010??????????????00??
???000?????0??????0????????2???00000?????
??????????????????????????????
Guanlong
0001000101112001000?10100?00?10000010?1000000?002??10000??0??00?100010000
01100010??010?10000?000010000000000?100000011?00100000?00000?????????????020?010
0000100000000000000????10?0000000010???????1010100???????0000001000001010000?0?00
000010000000100000000000001000000000000100000000000?0110000000010000000?00000000
000?10?00012?00000?0001000000000??1??001?110?110??0??0

Proceratosaurus
00?1001101112?01000?10100?00?1?000010?1???0?0?????????????????0?10001000001
10001?????0??0??0?0???100000000??0??0???????????????0000??000?????0??0?0???????00?1
00???????0001???110?000000001000?????111000??
??01?0??0??200000
0????????????0000????001?????1???????0
Yutyrannus
0101101101111?11002?00100??1?1?000010?11??000?01110?0000?00???0?1000??0?01
12?011210002?00??1????020?00????????1?????11?2?10?11???????0010000?????????????????
??????????01010?0?11?01020200100???00?11?000000?00?010100???00100?1????1000000000
000?0011?000?0000001??10001000011?0?10100100?01??1???[01]0010??00?00?00000000000
1????01221?000??021??0100?110??????0??110?110?????0
Dilong
0000001101002?01001?10000100?1?0000001000000000102??10100?00??000100011000
01100010??000?11000??0001000000010?????000000?00101000??????????????????020?0100
?0010?0??????01001??100?010002?00?11?????10101000000010000000100000????????0000
00110000001???0??010000100?0?000010??00010??000??011?00000000010000??0100110??00
0110000?2000000??0010000000000??1??0010??0?110??0??0
Sinotyrannus
???1???10?1?1?1110???0????0??1?00?????1??
???
?????????1?????????????????????????0???
?00?10?0??00001???????????????????????????????????????000???0??????0?2???00?????????
????????????01?1????????????
Eotyrannus
???0??11?100101110????????00?0?01????20110000?1000?1?1??0?00?00?????????????
???0?????????????????????????01?1???0??????????????????????001??????0?00????????????????
???????000?100002110100?????????2?2??00???00??0??00???1?0???????????00???011011011
??0000???00?1???????????????????????????????????01???000?0010011???0??111??11???????
??0?0???????????0?????010????010??????
Juratyrant
??
???
???????????????????????????????????0???????00?00?10000100?00100?0??????????????????????
???110000000001?1000110010010??????1100001?????????????11111????????11?????????????
???????????????????110??????????
Aviatyrannis
??
???
???
??10?00000000???????????????????????????????????????01110????????0???????????????????
??????????????????????????
Stokesosaurus
??
???
???

??10?00000001??????????????????????????????????????00111????????1??????????????????
?????????????1????????????

Xiongguanlong

00100?1102001????0??1??00?01?0?01?0011001010??0000?10100??0??0??0?????0???0
1000?0???00?01000???0021?111110010?1????100?1?111??100?100????????????00100100000
?10?1???????????????????????????????????2?2?000??000000000110000010?????????????????
????????????????11?101??0011??????????????01110012?????????????????11?0?00?10?0?0100
00?0???000100000?1110?1???1?1??0??10?????0

Raptorex

?1100?111200?0000010110001010010111022011010020000?201011001000000011000
000100000??000?0100???001??011???1?1??100111001?11111?????????0?????????0?????????
???100?0????001000001011121211??0011??0??212100000?0000000??100110??1?????0??011
0000212101111?11???111101111100010011???1112101??1??1?12?0?1??111?1????????0?000
011??????1?0101?1011?2??011111??????10?011?0100?????

Dryptosaurus

???????????????????????????????0??
??
????????0?01212?1?1??????????????0????????????????????????????????????10100?????1???
??11?????????????1???????12????????1012?1?011111?????1????????????????0???????????????
?????????????10??????????????

Appalachiosaurus

??1???????????000000?0100?01?010111??2011?1?1?0010?201010??0?0??????????????
???000000010100010?00???????????????
??????11?110??01??????????0??1????2?2?0?????????????????????????????0????????????????
?????????????????????1???????12111?111110???11111111111011?0?????0001???0??0???10??
?0?1?????????????????????1?0110??????

Albertosaurus

01101011120011000000[01]12001[01]1001011102201101111001112[01]111101000100
0000101010201002001110010010??113101111111110110121001111111110001000111[01][01
]1110?00110110111101111110111111110010111212111100?12?10?2121011????????11??????
??1?????????01111110212101111111?01011211111111110121111012111?21???1???1111111
1111112011000000001111000011010111121120?00?11112000??010010100?01100

Gorgosaurus

0110101112001?000000[01]120010100101110220110111200111201110010001000[01]
00101010201002001110010010?0113101111111110110121001111111110001000111111110?0
01101101111011111101111111110010111212111100112?10?2121011????????11????11101????
?1?01?111111021210101111110101121111111101101211111012111?111?11?2?111111111111
1201100000000011?10000110100111211101000011??000010100?0100100000

A.remotus

00?01????????????0????1?0??1?0?01???22011?1???0??????????????????????????????????
??1000?100???11??1?3??10??1?1?????01?1001?1111111???????????????????110?1011111101
1?11???01??????0??121??????????????????0???
???1??????????????1?1???1??????????????
?120001???1??000?????????1???????0

A.altai

001011????????0000011110111110?011102201101012011102011111101011100010010

1020000100001001011120113201111111100110121001111111?101110011011101000110110111111011011111011110010111212111110??10000????000001000001111110?11011?210111
11???????????????????????????1?111110110?????????21?11?11111121??00?11111?11?01100??
111111110?011?1011101112000111211110000?01?11?100?00?00

Qianzhousaurus

00?0111122001100002?11101111101011102201101012011102001111???1?10001002
?1020000200001001011120113201111111100110121 00?111111110???????1????????0011011
0111111?110?11?1011100010111 21?11??101110000????010101 1???0111?102?1011??????111
111?110???????????????????0??11110?10???????????????1??1012?11?0?11111????????00011
111?11?00?101011111112010111 21??00????10?10100?00?00

Daspletosaurus

011010112200111000201120021110221110220121111201110210 1[12]1111111111[01]
110120102000021100110 11[01]112111321211111110 01?0121111211111101011100 1100111
110011?1101111[01]1011111111111011110111222111101112?100212101100011101111 1102
11110??????1?1111111?212111?11111?111112111111111101?1111111 21111?12210?2?11?11
11111?11???1?00000011 1110?011010111211201011111110011101011100 1101110

D.n.sp.

01?0101?2?001?1000201[01]200211102211102201 21111?011102100111111111111111
12??0200002110011011111211132121111111 00110?211012111111010111001100111110011
01101101?101??111111?1011110111222121 1?1112110?2?21011111????0?11?1021 1110??????
?11???1???11???1??????????????11?11??1?1?121???11121?112122111211111111111111111?0
000000111110 00?10101111211201011111010011 10???11011101111

Tarbosaurus

1110111222001110112101211301102111102201 21101201???2100111111110110110 12
11021010211002101111121113212111111100101131212201111111111111011011111111111
10111002011111111111111120111222 12?1011121111212112111111?11111?10211111 1??1??
1?1111111021211101 1111?11111211111111111012111111112111121221112111111111111111 21
110000000001111000211111111221211101111?1011110101110100 00?00

Tyrannosaurus

11101112220011101121012[01]0[13]01102111102201 21111201???210011011111011[0
1]11012110210102110021011111211 13[12]121111111001111312122011111111111110110111
11111111110111002011111111111111112011122212110111211112121121111111111111110211
1101211111111111111021211101111111111121111111111101211111112111121221112111111 1
111111121110000000011110002111111112212111011111101111010111010100 00[01]

Bistahieversor

?0?010111200110000 0?10210101?01011102201101101001012000100 10??100000?00?
00020000211001001000 01??1311111 1?1?1001101210012111111 1?00000?010001110?????11
?1111[01]101?110111111111 00?01112121011001 12?10?2?2?00????111?????????????????????0
111111???111?1????0110111111?11001?????00
011?10??0110100??121120100?111?2?00?1????10000100?00

Teratophoneus

??????????????0000?????0?11?0101??02?????????2?0110201010?1010?0??????0???02?
1??20100200111???0?1311211111?10011012111111111111???????????????????1?011?110102
011?1?1?11??0100?011122211???111??1??????10?????????1?11?102????????????111????1?021
1111011??????????1??1?111?1?121111111 2?111111211121?1?111???????????0000??0111??0
0?1????1?112010??001111010?0?10?001??00?10?00

Lythronax

????1?????????000000?0?10?0110111?102201001?1?0?????????????1???00100?020102
0000?????????????????????1?11??1001?01211?1?1111?????????????????????????????????????
?????1??1?????01?12221211??????1??????1???
????????????????????121111?11???????????????1?111?1??????????????1?0??1???00???10011
????1?????11111???????0??00???00?0?

Zhuchengtyrannus

??????????????10?0??????0??110?11?102???
??
????1111?011?????????????????????11???
??0????????????1?11????????
???????????10?????0????????

Nanuqsaurus

???
????????????????????????????01?0??11?1?11110?1??
???110?10?1???
??020????
????????????????0???1????

ccode

/1-[0 /1-[1 /1-[2 /1-[3 /1-[4 /1-[5
/1-[6 /1+[7 /1+[8 /1+[9 /1-[10 /1-[11
/1+[12 /1-[13 /1-[14 /1-[15 /1-[16 /1-[17
/1+[18 /1-[19 /1-[20 /1-[21 /1+[22 /1-[23
/1-[24 /1+[25 /1-[26 /1-[27 /1-[28 /1-[29
/1+[30 /1-[31 /1-[32 /1-[33 /1-[34 /1-[35
/1+[36 /1+[37 /1-[38 /1-[39 /1-[40 /1-[41
/1-[42 /1-[43 /1-[44 /1-[45 /1-[46 /1-[47
/1-[48 /1-[49 /1-[50 /1+[51 /1-[52 /1-[53
/1-[54 /1-[55 /1-[56 /1-[57 /1-[58 /1-[59
/1-[60 /1-[61 /1-[62 /1-[63 /1-[64 /1-[65
/1-[66 /1-[67 /1-[68 /1-[69 /1-[70 /1+[71
/1-[72 /1-[73 /1-[74 /1+[75 /1-[76 /1-[77
/1-[78 /1-[79 /1+[80 /1-[81 /1-[82 /1-[83
/1-[84 /1+[85 /1-[86 /1-[87 /1-[88 /1-[89
/1-[90 /1-[91 /1-[92 /1+[93 /1-[94 /1-[95
/1-[96 /1+[97 /1+[98 /1-[99 /1-[100 /1-[101
/1-[102 /1-[103 /1-[104 /1-[105 /1-[106 /1-[107
/1-[108 /1-[109 /1-[110 /1-[111 /1-[112 /1-[113
/1+[114 /1-[115 /1-[116 /1-[117 /1+[118 /1+[119
/1-[120 /1-[121 /1-[122 /1-[123 /1-[124 /1-[125
/1-[126 /1-[127 /1-[128 /1-[129 /1-[130 /1-[131
/1-[132 /1-[133 /1-[134 /1-[135 /1-[136 /1-[137
/1-[138 /1-[139 /1-[140 /1-[141 /1-[142 /1-[143
/1-[144 /1-[145 /1-[146 /1-[147 /1-[148 /1-[149
/1-[150 /1-[151 /1-[152 /1-[153 /1-[154 /1-[155

```
/1-[156  /1+[157  /1-[158  /1-[159  /1-[160  /1-[161
/1-[162  /1-[163  /1-[164  /1-[165  /1-[166  /1-[167
/1-[168  /1-[169  /1-[170  /1-[171  /1-[172  /1-[173
/1-[174  /1-[175  /1-[176  /1-[177  /1-[178  /1+[179
/1+[180  /1-[181  /1-[182  /1+[183  /1-[184  /1-[185
/1-[186  /1-[187  /1-[188  /1-[189  /1+[190  /1-[191
/1-[192  /1-[193  /1-[194  /1+[195  /1-[196  /1+[197
/1-[198  /1-[199  /1+[200  /1-[201  /1-[202  /1-[203
/1-[204  /1-[205  /1-[206  /1-[207  /1-[208  /1-[209
/1-[210  /1-[211  /1-[212  /1-[213  /1-[214  /1-[215
/1+[216  /1-[217  /1-[218  /1-[219  /1-[220  /1-[221
/1-[222  /1+[223  /1-[224  /1-[225  /1-[226  /1-[227
/1-[228  /1-[229  /1-[230  /1-[231  /1-[232  /1-[233
/1-[234  /1-[235  /1-[236  /1-[237  /1+[238  /1-[239
/1+[240  /1-[241  /1-[242  /1-[243  /1-[244  /1-[245
/1-[246  /1-[247  /1-[248  /1-[249  /1-[250 /1-[251
/1-[252    /1-[253  /1-[254  /1-[255  /1+[256  /1-[257
/1-[258    /1-[259  /1-[260    /1-[261  /1-[262    /1-[263
/1-[264    /1-[265  /1-[266  /1-[267  /1-[268  /1+[269
/1-[270  /1-[271  /1-[272  /1-[273  /1-[274       /1-[275
/1-[276  /1+[277  /1-[278  /1-[279  /1-[280  /1-[281
/1+[282  /1-[283  /1+[284  /1+[285  /1-[286  /1-[287
/1-[288  /1+[289  /1-[290  /1-[291  /1-[292  /1-[293
/1-[294  /1-[295  /1-[296  /1-[297  /1-[298  /1-[299
/1-[300  /1-[301  /1-[302  /1-[303  /1+[304  /1-[305
/1-[306  /1-[307  /1-[308  /1-[309  /1-[310  /1-[311
/1-[312  /1-[313  /1-[314  /1-[315  /1-[316  /1-[317
/1-[318  /1-[319  /1-[320  /1-[321  /1+[322  /1+[323
/1-[324  /1-[325  /1-[326  /1-[327  /1-[328  /1-[329
/1-[330  /1-[331  /1+[332  /1+[333  /1-[334  /1-[335
/1-[336  /1-[337  /1-[338  /1-[339  /1-[340  /1-[341
/1+[342  /1-[343  /1-[344  /1+[345  /1-[346  /1-[347
/1-[348  /1-[349  /1-[350  /1-[351  /1-[352  /1-[353
/1-[354  /1-[355  /1-[356  /1-[357  /1-[358  /1-[359
/1-[360  /1-[361  /1-[362  /1-[363  /1-[364  /1-[365;

;

proc/;
```

4. Comments on Loewen *et al*. (2013)

One of the main goals of the present study was to critically review the phylogenetic characters of Loewen et al. (2013), a comprehensive analysis of tyrannosauroid genealogy that appeared after the first version of our dataset (Brusatte et al. 2010) and incorporated new taxa and characters. Our aim was to merge the datasets of Brusatte et al. (2010) and Loewen et al. (2013) to produce the largest and most up-to-date phylogenetic analysis for tyrannosauroids.

We here provide commentary on the Loewen et al. (2013) dataset. Many of the characters in Loewen et al. (2013) were used in the Brusatte et al. (2010) analysis, so they do not deserve further comment. Additionally, several of the Loewen et al. (2013) characters were incorporated into our present study, and appear in our new character list (Section 2 above), and because we accept these characters they do not warrant additional comment. The characters we discuss below are those that we did not elect to include in our new merged dataset. The list may appear long, but many of these characters are dismissed because they are relevant to higher-level theropod phylogeny (the relationships of the outgroups in the Loewen et al. analysis) and are invariant among tyrannosauroids, and therefore would not be relevant to our tyrannosauroid-specific dataset. We dismiss other characters for the reasons we outline below.

Note that any character that Loewen et al. (2013) cited as appearing in Brusatte et al. (2010) is not discussed below, unless Loewen et al. (2013) heavily modified the character. Additionally, in those cases that characters overlap between the two datasets, but Loewen et al. (2013) did not cite their character as appearing in Brusatte et al. (2010), we note this below for clarity's sake.

<u>Character 3</u>: Rostrum length compared to posterior skull length (measured from the anterior premaxilla to midpoint of the lacrimal bar divided by basal skull length to end of quadrate):
(0) – longirostran, rostrum more than 0.65
(1) – rostrum 0.55 - 0.65 of posterior skull
(2) – brevirostrine, rostrum 0.4 - 0.55

We judge this character to be redundant with character 2 in our dataset, which is also used by Loewen et al. (2013:11), which quantifies the overall length:height ratio of the skull.

<u>Character 7</u>: Laterotemporal fenestra, posterior bar:
(0) – ascending rami of quadrate and quadratojugal meet squamosal to form a straight posterior border
(1) – angled (kinked) posterior border of the laterotemporal fenestra in lateral view

This character is redundant with character 98 in our dataset.

<u>Character 9</u>: Skull width vs skull length:
(0) – narrow, 37% or less than premaxilla-quadrate length
(1) – 40% - 58% of premaxilla-quadrate length
(2) – wide, more than 63% of premaxilla-quadrate length

We think that this character is partially redundant with two other characters in our dataset: character 2 (which encapsulates skull height:length) and character 326 (which encapsulates the

direction the orbit faces, with forward-facing orbits seen in taxa that have very wide skulls). We consider characters 3, 9, and 11 in the Loewen et al. (2013) dataset to all generally be referring to the same feature: the general proportions of the skull, particularly the depth of the skull relative to its height.

Character 10: Snout width, mediolateral width of the snout at the level of the posterior-most maxillary tooth:
(0) – twice or less the width of nasals
(1) –approximately three times the width of the nasals

This character was originally used in the datasets of Brusatte et al (2009) and Carr and Williamson (2010), but we excluded it in the Brusatte et al. (2010) analysis when critically reviewing previous characters used in the literature. We continue to exclude it here. We also note that it is is difficult to obtain a snout width measurements from skulls that cannot be accessed from below (palatal view).

Character 13: Premaxilla, premaxillary symphasis, overall shape in dorsal view:
(0) – acute, V-shaped
(1) – rounded, U-shaped
(2) – transverse, rostrally facing

This is redundant with our character 10, which denotes the orientation of the premaxillary tooth row, another way of specifying whether a taxon has a V-shaped or U-shaped anterior margin of the premaxilla.

Character 15: Anterior margin of the premaxillary body in lateral view:
(0) - inclined and extends caudodorsally
(1) - or extends nearly vertically

State 1 is present in all tyrannosauroids (Brusatte et al. 2014), so it is not relevant for an in-group phylogenetic analysis.

Character 18: Premaxilla, subnarial process length:
(0) – subnarial process absent
(1) – present but much shorter than narial process
(2) – subequal to length of narial process
(3) – much longer than narial process

Only two states are relevant to tyrannosauroids: subnarial process shorter than the narial process (1) or much longer than the narial process (3). However, based on our reexamination of specimens, we find that all tyrannosauroids have a subnarial process that is equal to or longer than the narial process, and we do not find evidence for any clear discrete cut-off in subnarial process proportions among tyrannosauroids.

Character 23: Premaxillary interdental plates:
(0) – fused

(1) – unfused

State 1 is present in all tyrannosauroids, so it is not relevant for an in-group phylogenetic analysis.

Character 25: Maxilla, constriction between articulated premaxillae and maxillae:
(0) – absent
(1) – present

State 0 is present in all tyrannosauroids, so it is not relevant for an in-group phylogenetic analysis.

Character 26: Maxilla and premaxilla contact along alveolar margins:
(0) – in contact at alveolar magins
(1) – alveolar margins do not contact

State 0 is present in all tyrannosauroids, so it is not relevant for an in-group phylogenetic analysis.

Character 27: Maxilla, orientation of the maxillae towards each other in dorsal view:
(0) – acutely angled
(1) – subparallel

All tyrannosauroids except for a few highly derived large-bodied taxa (e.g., *Tyrannosaurus*) are scored for state 1. The condition of acutely angled maxillae (state 0) in derived taxa is due to their wide skulls, which is encapsulated in our character 326, which quantifies the orientation of the orbit (taxa with forward-facing orbits have wide skulls).

Character 28: Maxilla, anterior margin, premaxillary suture on body of maxilla in lateral view:
(0) – sloping
(1) – vertical

State 0 is present in all tyrannosauroids, so it is not relevant for an in-group phylogenetic analysis.

Character 30: Maxilla, pneumatic excavation/antrum in maxillary anterior process:
(0) – absent
(1) – present

State 1 is present in all tyrannosauroids, so it is not relevant for an in-group phylogenetic analysis.

Character 31: Maxilla, vertical height at inflection point of anterior end of antorbital fenestra versus total height:
(0) – short, less than 40% total height
(1) – more than 50% total height

(2) – tall, more than 60% total height

We judge this character to be redundant with other characters having to do with the general height:length proportions of the skull, particularly character 2 in our dataset. Taxa with long and low skulls are scored for state 0, those with intermediate skull proportions are scored as state 1, and those with proportionally deep skulls are scored for state 2. We also note (see above in discussing of Character 9) that the Loewen et al. (2013) dataset includes many characters that we think are generally relating to the same feature: the height:length proportions of the skull.

Character 32: Maxilla, promaxillary fenestra:
(0) – absent
(1) – present

State 1 is present in all tyrannosauroids, so it is not relevant for an in-group phylogenetic analysis.

Character 44: Maxilla, palatal shelf of maxilla dorsal to interdental plates:
(0) – unexpanded mediolaterally
(1) – broad mediolateral expansion forming shelf subequal to or wider than alveolar body

This character concerns the “secondary palate” of tyrannosauroids and some other coelurosaurs, which is the condition scored by state 1. This condition is present in all tyrannosauroids and some close outgroups (Brusatte et al. 2014), so it is invariant among tyrannosauroids and not relevant for an in-group phylogenetic analysis.

Character 48: Nasals, fusion along the midline:
(0) – unfused along entire length
(1) – at least partially fused

State 1 is present in all tyrannosauroids that can be scored, so it is not relevant for an in-group phylogenetic analysis.

Character 50: Nasal, crest fenestration:
(0) – not fenestrate
(1) – fenestrate

State 1 is present in all tyrannosauroids with a crest, so it is not relevant for an in-group phylogenetic analysis.

Character 51: Nasal, crest, dorsal wing of lacrimal wraps onto side of nasal crest:
(0) – absent
(1) – present

State 0 is present in all tyrannosauroids with a crest, so it is not relevant for an in-group phylogenetic analysis.

Character 55: Nasal, dorsal surface of the posterior end of the nasal (frontal process only):
(0) – flat
(1) – convex
(2) – concave

We think that this character is redundant with character 42 in our dataset, which concerns the constriction of the nasal in dorsal view. The constriction of the nasal is what causes a deep fossa to occur. This character was in the dataset of Brusatte et al. (2009) but was excluded when we critically reviewed characters for the Brusatte et al. (2010) analysis.

Character 56: Nasal, in dorsal view, mediolateral expansion of anterior end, anterior to the maxillary fenestra:
(0) – absent
(1) – present

State 1 is present in all tyrannosauroids, so it is not relevant for an in-group phylogenetic analysis.

Character 57: Nasal, in dorsal view, posterior end:
(0) – the same width as the rest of the element
(1) – restricted, posterior 1/3 of element narrower than the anterior 2/3
(2) – expanded, posterior 1/3 of element wider than the anterior 2/3

This character is redundant with character 42 in our dataset.

Character 58: Nasal, dorsal surface in lateral view (excluding crests):
(0) – straight or convex, relatively flat
(1) – concave with ventral deflection

State 0 is present in all tyrannosauroids, so it is not relevant for an in-group phylogenetic analysis.

Character 60: Nasal, frontal suture shape:
(0) – simple wedge (V-shaped)
(1) – W-shaped
(2) – transverse or gentle U-shaped

Character 46 in our dataset concerns the shape of this suture, so although this character from Loewen et al. (2013) is worded somewhat differently, we considered it redundant with our character and retain our original usage.

Character 62: Lacrimal, anterior process length:
(0) – longer than ventral process
(1) – from 90% to 70% the length of the ventral process
(2) – short less than 65% ventral process length

Based on our own comparative study of tyrannosauroids, we find that there is incredible variation in the ratio between the anterior and ventral processes of the lacrimal. This variation does not have a clear phylogenetic signal, as taxa that are closely related (based on other characters) often have widely different values. This character was originally used in higher-level analyses of theropod phylogeny (see character citations in Loewen et al. 2013), where it has a clearer phylogenetic signal. We suspect that it is recording random variation in tyrannosauroids, as well as possible variation among individuals and ontogenetic stages. Therefore, we conservatively exclude this character.

Character 64: Lacrimal, dorsal crest, height in lateral view:
(0) – low, less than the width of the anterior/posterior length of the descending process
(1) – relatively tall, at least as tall as the anterior/posterior length of the descending process

There are several characters in our dataset relating to the cornual process (=dorsal crest) of the lacrimal, and we think that this character is at least partially redundant with those in our dataset. It is also unclear from this character statement where the height and width measurements are taken, so we could not reproduce the character scores in Loewen et al. (2013). For these two reasons we conservatively exclude this character.

Character 65: Lacrimal, dorsal crest, cross-section in anterior view:
(0) – pinched or squared
(1) – rounded

This is redundant with character 53 in our dataset, which concerns the inflation of the cornual process of the lacrimal.

Character 66: Lacrimal, dorsal crest, shape in lateral view:
(0) – sharp, with crest forming distinct peak
(1) – rounded
(2) – rounded peak

We judge this character to be redundant with characters 49 and 50 in our dataset, which concern the size and shape of the cornual process of the lacrimal. We are of the opinion that the Loewen et al. (2013) dataset is over-atomizing characters of the cornual process, and our preference is for a smaller number of more straightforward characters having to do with the presence, shape, and position of the process. Our strategy is similar to that which Nesbitt (2011) outlined for encapsulating femoral head morphology in early dinosaurs and close relatives and Brusatte et al. (2010b) outlined for scoring theropod cranial crest morphology.

Character 67: Lacrimal, dorsal crest, position of the apex of the ornamentation in lateral view:
(0) – apex anterior to posterior edge of antorbital fenestra
(1) – apex posterior to posterior edge of antorbital fenestra

This is redundant with character 51 in our dataset.

Character 69: Lacrimal, cornual boss on posterodorsal process:

(0) – absent
(1) – present, boss forms ridge or shelf so that ridge and rounded crest are sigmoidal in dorsal view
(2) – present, forms massive rounded boss

We think that this character is an amalgamation of features relating to the cornual process (which is already represented by several characters in the Loewen et al. dataset: see comments on character 66 above) and the inflation of the posterior process of the lacrimal (which is represented in character 62 in our dataset).

Character 71: Lacrimal, accessory pneumatic recess on anterior process of lacrimal:
(0) – absent
(1) – present

Character 72: Lacrimal, position of accessory pneumatic recess:
(0) – posterior, much closer to ventral process than anterior end of anterior process
(1) – anterior, much closer to anterior end of anterior process than ventral process

Both of these characters, 71 and 72, are redundant with character 56 in our dataset, which we have modified here relative to the original Brusatte et al. (2010) study.

Character 75: Lacrimal, lacrimal vacuity opening laterally at the angle of the lacrimal above antorbital fenestra:
(0) – absent
(1) – present, large, where the vacuity extends posteriorly to the ventral process at least ½ width of ventral process
(2) – tiny, much less than ½ width ventral process where the vacuity ends cranial to the ventral process

Character 76: Lacrimal, lacrimal vacuity, height to length ratio:
(0) – tall, greater than 1.1
(1) – short, less than 0.9
(2) – subequal

This character was originally used by Brusatte et al. (2009) but was excluded when we critically revised characters for the Brusatte et al. (2010) phylogenetic study, as we felt that there was no clear discrete distinction between taxa. Given the very close numerical cut-offs between states, this character is also difficult to score accurately, and deformation can easily lead to incorrect scores.

Character 77: Lacrimal, lacrimal vacuity, rim of lacrimal vacuity flush with rim of the antorbital fossa:
(0) – flush with ventral surface
(1) – rimmed

This is redundant with character 55 in our dataset.

Character 78: Lacrimal, lacrimal vacuity, posterodorsal margin of lacrimal vacuity:
(0) – rimmed
(1) – rounded

We do not understand this character. The posterodorsal margin of the lacrimal vacuity is always rimmed and rounded in tyrannosauroids. We do not see any differences between taxa that Loewen et al. (2013) score as 0 (e.g., *Albertosaurus, Alioramus*) and 1 (e.g., *Bistahieversor, Tyrannosaurus*). In all of these taxa the posterodorsal corner of the lacrimal vacuity is separated from the remainder of the lacrimal by a rounded rim. We also do not understand how "rimmed" and "rounded" are different states for the same character, as they seem to be referring to different features (presence or absence of rim along the margin and the shape of the margin).

Character 82: Prefrontal:
(0) – present
(1) – absent

State 0 is present in all tyrannosauroids, so it is not relevant for an in-group phylogenetic analysis.

Character 86: Anterior extent of the prefrontal position relative to lateral part of frontal process of nasal:
(0) – anterior to frontal process of nasal
(1) – even with or lateral to frontal process of nasal
(2) – posterior to frontal process of nasal

Based on our observations, all tyrannosaurids (except for possibly *Daspletosaurus torosus*) have state 1. The basal tyrannosauroid *Guanlong* also has state 1 (contra Loewen et al.'s score of 0 for this taxon). Therefore, this character is invariant (or nearly so) among tyrannosauroids, and if *Daspletosaurus torosus* does possess state 0 then it is an autapomorphy.

Character 87: Prefrontal, contact with lacrimal and frontal:
(0) – prefrontal separates lacrimal and frontal
(1) – lacrimal and frontal contact each other behind the prefrontal
(2) – lacrimal and frontal contact each other in front of the prefrontal

We judge this character to be redundant with character 110 in our dataset, which concerns the presence or absence of contact between the prefrontal and nasal. Only *Guanlong* and *Dilong* are scored for state 0 by Loewen et al. (2013), and this is in part caused by the prefrontal contacting the nasal. All other tyrannosauroids are scored for state 1, and this is in part because the prefrontal and nasal do not make broad contact in dorsal view.

Character 88: Frontal, anterior margin:
(0) – narrow anteriorly as an elongate wedge between nasals
(1) – anterior end terminates abruptly even if w shaped, suture with nasal transversely oriented

This is a modification of character 113 in our dataset, which had been used by previous authors. We do not think that this modification is necessary. The modified Loewen et al. (2013) character links together *Bistahieversor* and tyrannosaurines based on their shared possession of state 1, but we do not understand what morphology these tyrannosauroids share relative to other taxa.

Character 91: Frontal, shape of the prefrontal articular surface on dorsal surface of skull:
(0) – straight or rostromedial arc
(1) – notch

This character was originally used by Brusatte et al. (2009) but was excluded when we critically revised characters for the Brusatte et al. (2010) phylogenetic study, as we felt that there was no clear, quantitative, discrete distinction between “arc” and “notch”.

Character 92: Frontal, expansion of supratemporal fossa onto dorsal surface:
(0) – supratemporal fossa extends anteriorly onto dorsal surface of frontal
(1) – supratemporal fenestra excluded from frontal

State 0 is present in all tyrannosauroids, so it is not relevant for an in-group phylogenetic analysis. Note that Loewen et al. (2013) score *Daspletosaurus* n. sp. for state 1, but this is a mis-scoring, as this taxon possesses a supratemporal fossa that extends onto the frontal as in other tyrannosauroids.

Character 93: Frontal, supratemporal fossa, anterior emargination:
(0) – straight or curved
(1) – sinusoidal

We cannot recognize a clear, quantitative, discrete difference between “straight or curved” and “sinusoidal”, so we find this character too difficult to score accurately and consistently.

Character 101: Parietals, dorsal surface in lateral view:
(0) – generally flat or concave
(1) – arched, dorsally convex

State 0 is present in all tyrannosauroids, so it is not relevant for an in-group phylogenetic analysis.

Character 102: Parietals, shape of flat or concave dorsal surface in lateral view:
(0) – generally flat
(1) – shallowly concave
(2) – deeply concave with depth of concavity approaching ½ of length

We think that this character is at least partially redundant with characters 97 and 100 in the Loewen et al. (2013) analysis (having to do with the frontoparietal peak), which are both included in our dataset, as the shape of the dorsal surface of the parietal when seen in lateral view is largely dependent on the presence/absence and height (strength) of the frontoparietal peak.

Character 103: Parietals, nuchal crest height:
(0) –subequal to entire roof of the skull
(1) – equal to or shorter than tallest part of the skull
(2) – taller than any other part of the skull

This character is included in our dataset as character 126.

Character 106: Postorbital, anterior process curvature in lateral view:
(0) – straight anterior (frontal) process
(1) – anterior process strongly arched anterodorsally

State 0 is present in all tyrannosauroids, so it is not relevant for an in-group phylogenetic analysis.

Character 107: Postorbital, descending process:
(0) – relatively narrow and comparable to preorbital bar (lacrimal) in anterior/posterior width
(1) – at least the midpoint of the postorbital bar (formed by descending process of postorbital and sometimes ascending process of jugal) wide and plate-like, about twice as wide as preorbital bar

This is included in our dataset as character 89.

Character 110: Postorbital, ornamentation:
(0) – absent
(1) – present low sculpturing
(2) – present cornual boss of rugose grooves
(3) – present as round knobby cornual boss

Although identified as a new character by Loewen et al. (2013), this is included in our dataset as character 81.

Character 111: Postorbital, cornual process, position relative to caudal margin of orbit:
(0) – cornural process positioned posterior to orbit margin
(1) – cornual process confluent from posterior margin of orbit

Although identified as a new character by Loewen et al. (2013), this is included in our dataset as character 83.

Character 112: Cornual process undercut at base, so that process is differentiated from the rest of the lateral surface:
(0) – absent
(1) – present

This character was originally used by Brusatte et al. (2009) but was excluded when we critically revised characters for the Brusatte et al. (2010) phylogenetic study, as we felt that it was difficult to quantitatively, or even qualitatively, define what an “undercut” cornual process is.

Character 115: Postorbital, expansion of supratemporal fossa onto dorsal surface of postorbital:
(0) – no supratemporal fenestra on postorbital
(1) – covers most of frontal process of the postorbital and extends anteriorly onto dorsal surface of frontal

State 1 is present in all tyrannosauroids, so it is not relevant for an in-group phylogenetic analysis.

Character 118: Squamosal, extends onto supratemporal fossa:
(0) – absent, fenestra bounded laterally and posteriorly by the squamosal
(1) – present, fossa extends onto dorsal surface of the squamosal

State 0 is present in all tyrannosauroids, so it is not relevant for an in-group phylogenetic analysis.

Character 119: Squamosal, web of bone overhangs the caudal border of the supratemporal fossa:
(0) – absent
(1) – present

This character was originally used by Brusatte et al. (2009) but was excluded when we critically revised characters for the Brusatte et al. (2010) phylogenetic study, as our comparative examinations show that this web (state 1) is present in all tyrannosauroids. Loewen et al. (2013) scores *Raptorex*, *Xiongguanlong*, *Dilong*, and *Guanlong* for state 0. However, *Raptorex* and *Xiongguanlong* possess the web of bone, whereas *Dilong* and *Guanlong* are too poorly preserved for us to be certain. Therefore, all tyrannosauroids that can be scored are either scored as 1 or ?, meaning this character is not relevant for an in-group phylogenetic analysis.

Character 120: Squamosal, orientation of web of bone that overhangs supratemporal fossa:
(0) – dorsal
(1) – lateral

State 0 is present in all tyrannosauroids, so it is not relevant for an in-group phylogenetic analysis.

Character 123: Squamosal, non-vertical quadratojugal process, extent of constriction of the laterotemporal fenestra:
(0) – process constricts less than 80%
(1) – process constricts more than 90%

We consider this character as equivalent to character 98 in our dataset, which is also used as character 147 in the Loewen et al. (2013) analysis). The Loewen et al. analysis is essentially including this character twice.

Character 125: Squamosal, horizontal quadratojugal process, relationship of the articulation with quadratojugal:

(0) – horizontal process with rod-like ridge above quadratojugal and forming lap joint medial to the quadratojugal
(1) – process forms a broad lap joint with quadratojugal, but visible portion of horizontal process above quadratojugal is tab-like

This is a modified version of character 94 in our dataset. We prefer to retain our original character usage, as we find that the descriptions of Loewen et al.'s (2013) character states are difficult to follow.

Character 127: Squamosal, fossa in ceiling of bone, shape:
(0) – broad with rounded margin
(1) – rimmed with sharp margins

We consider this character to be generally equivalent to Loewen et al.'s (2013) character 126, which concerns squamosal pneumaticity, which we incorporate into our dataset here. Both characters have to do with the presence or absence of a large pneumatic recess in the ceiling of the squamosal. We do not understand, however, the difference between "rounded" and "sharp" margins of this recess, so we discard this specific character.

Character 128: Squamosal, postquadratic process thickness in lateral view measured perpendicular to long axis of the process in the dorsoventral or cranioposterior plane in lateral view:
(0) – absent or thin
(1) – subequal but not thin
(2) – thicker than long

We judge this character to be equivalent to character 95 in our dataset, which concerns the inflation (or lack thereof) of the posterior process of the squamosal by an internal pneumatic recess. This inflation governs the thickness of the process, the feature that is encapsulated in Loewen et al.'s (2013) character 128.

Character 129: Squamosal, postquadratic process:
(0) – short
(1) – at least as long as quadrate cotylus
(2) – twice as long as quadrate cotylus
(3) – three or more times as long as the quadrate cotylus

This character is generally equivalent to character 96 in our dataset.

Character 130: Jugal, contribution to antorbital fenestra:
(0) – absent
(1) – participates along a small surface in the antorbital fenestra
(2) – extensive, forms the caudoventral corner of antorbital fenestra

State 2 is present in all tyrannosauroids, so it is not relevant for an in-group phylogenetic analysis.

Character 131: Jugal, pneumatic recess:
(0) – absent
(1) – small and round
(2) – large and elongate

State 2 is present in all tyrannosauroids, so it is not relevant for an in-group phylogenetic analysis.

Character 132: Jugal, pneumatic recess orientation of long axis:
(0) – axis of recess is horizontal
(1) – axis of foramen inclined at an angle of 45 degrees to the ventral skull margin

This is included in our dataset as character 68.

Character 133: Jugal, position of pneumatic recess in antorbital fossa:
(0) – antorbital fossa edge does not extend past the pneumatic recess
(1) – antorbital fossa edge is continues caudoventral to the pneumatic recess

This character is credited to Brusatte et al. (2009: character 68), but the 2009 character relates to the lacrimal and not the jugal. Based on our comparative study of tyrannosauroids, we do not recognize any clear, discrete variation among taxa in the extent of the antorbital fossa edge relative to the pneumatic recess, as in all taxa we have studied the fossa edge does not extend past the pneumatic recess.

Character 134: Jugal, maxillary process, antorbital fossa position relative to ventral process of lacrimal:
(0) – posterior edge of fossa posterior to or directly ventral to midpoint of ventral process
(1) – posterior edge of fossa anterior to midpoint of ventral process

We consider this character to be equivalent to character 67 in our dataset.

Character 137: Jugal, suboccular process on ventral part of orbit to accept the suboccular flange of the postorbital:
(0) – absent, postorbital process confluent with suborbital margin
(1) – present, slight notch between postorbital articulation and suborbital margin
(2) – present, pronounced flange corresponding to suboccular flange of postorbital

This is equivalent to character 72 in our dataset, with an added state (state 2). We add this new state to our dataset here.

Character 139: Jugal, postorbital facet of ascending process:
(0) – absent or shallow
(1) – mediolaterally oriented scarf joint

This character is credited to Brusatte et al. (2009: character 75), but it does not correspond to the 2009 character, which relates to the antorbital fossa on the jugal. We do not understand this character and are concerned that it may be redundant with other features of the jugal-postorbital articulation that we do score in our dataset (characters 71-74).

Character 140: Jugal, postorbital process, orientation relative to ventral margin of jugal in life position:
(0) – approximately perpendicular
(1) – posteriorly inclined 15-25 degrees from perpendicular
(2) – strongly posteriorly inclined to 30 or more degrees

This is a modified version of character 75 in our dataset, with an intermediate state added. Our original character differentiates between only an approximately perpendicular postorbital process and one that is inclined posterodorsally. We do not include the intermediate state in our dataset here for two reasons. First, we do not wish to over atomize this character and hypothesize that the primary distinction is between two quite distinct states: perpendicular or inclined. Second, there is some evidence that the orientation of the process changes during ontogeny, as the process is somewhat posterodorsally oriented in juvenile *Gorgosaurus* but essentially perpendicular in adults (TDC, pers. obs.). Therefore, we suspect that taxa with something of an intermediate condition are in the midst of this ontogenetic transformation. We do our best to score only adult specimens for this character, except in those cases where no adults are known for a taxon (e.g., *Raptorex*).

Character 142: Jugal, ventral margin:
(0) – ventral margin relatively straight or gently convex
(1) – ventral margin convex rugose peak

Although identified as a new character by Loewen et al. (2013), this is included in our dataset as character 76. The ‘convex rugose peak’ is equivalent to the cornual process.

Character 148: Quadratojugal, anterior process, dorsoventrally expanded so majority of process overlaps laterally onto jugal:
(0) – absent
(1) – present

We consider this to be redundant with character 101 in our dataset, which is also included in the Loewen et al. (2013) dataset as character 149. Those taxa with rounded or squared-off tab-like anterior processes (scored in our character 101) are those that have the ‘dorsoventrally expanded’ process (as scored in Loewen et al. character 148).

Character 150: Quadratojugal, squamosal-quadratojugal contact:
(0) – at tips or absent
(1) – broad

State 1 is present in all tyrannosauroids, so it is not relevant for an in-group phylogenetic analysis.

Character 151: Quadratojugal, anteroposterior breadth of quadratojugal dorsal process:
(0) – narrow
(1) – broad

State 1 is present in all tyrannosauroids, so it is not relevant for an in-group phylogenetic analysis.

Character 155: Quadrate foramen size:
(0) – absent
(1) – small less than 3% element dorsoventral height
(2) – more than 5% element height but less than 40% element height
(3) – more than 45% element height

This character is essentially equivalent to character 105 in our dataset, although with different numerical cut-offs for the scores. We prefer our original character language.

Character 156: Quadrate foramen conformation:
(0) – almost completely formed within the quadrate
(1) – 75% of foramen formed by the quadrate
(2) – formed equally by the quadrate and quadratojugal

State 0 is present in all tyrannosauroids, so it is not relevant for an in-group phylogenetic analysis.

Character 157: Quadrate foramen orientation:
(0) – caudally, not visible in lateral view
(1) – caudolaterally, visible in lateral view

We do not understand this character. In no tyrannosauroids is the quadrate foramen visible in lateral view. For example, Loewen et al. (2013) score *Alioramus altai* for state 1, but the foramen is not visible in lateral view, only posterior view (Brusatte et al. 2012:figs. 21, 24).

Character 163: Supraoccipital, distinct dorsal process of supraoccipital:
(0) – absent
(1) – present

State 1 is present in all tyrannosauroids, so it is not relevant for an in-group phylogenetic analysis.

Character 164: Supraoccipital, width of dorsal process of supraoccipital:
(0) – narrow
(1) – wide

This character was originally used by Brusatte et al. (2009) but was excluded when we critically revised characters for the Brusatte et al. (2010) phylogenetic study, as we felt that there was no

clear quantitative distinction between 'narrow' and 'wide'. As Loewen et al. (2013) have not provided such a distinction, we continue to exclude this character.

Character 165: Supraoccipital, dorsal process of supraoccipital form of dorsal surface:
(0) – flat or peaked
(1) – forked

This is equivalent to character 149 in our dataset, which is also used as character 162 in the Loewen et al. (2013) dataset. Therefore, Loewen et al. (2013) included the same character twice.

Character 170: Opisthotic, posterior (caudal) tympanic recess:
(0) – absent
(1) – present

State 1 is present in all tyrannosauroids, so it is not relevant for an in-group phylogenetic analysis.

Character 171: Opisthotic, posterior (caudal) tympanic recess position:
(0) – posterior
(1) – anterior

This character was originally used by Brusatte et al. (2009) but was excluded when we critically revised characters for the Brusatte et al. (2010) phylogenetic study, as we felt that there was no clear quantitative distinction between a 'posterior' and an 'anterior' position. As Loewen et al. (2013) have not provided such a distinction, we continue to exclude this character.

Character 172: Opisthotic, ventral extension:
(0) – notch separates basal tuber from more anteroventral extension of exoccipital-basisphenoid suture
(1) – notch absent

State 1 is present in all tyrannosauroids, so it is not relevant for an in-group phylogenetic analysis.

Character 173: Opisthotic, orientation of the paroccipital processes:
(0) – directed laterally, or slightly ventrolaterally
(1) – directed strongly ventrolaterally

State 0 is present in all tyrannosauroids, so it is not relevant for an in-group phylogenetic analysis.

Character 174: Opisthotic, ventral extent of the paroccipital processes:
(0) – above or level with the dorsal border of the occipital condyle
(1) – situated at mid-height of occipital condyle or lower

State 1 is present in all tyrannosauroids, so it is not relevant for an in-group phylogenetic analysis.

Character 175: Opisthotic, curvature of the ventral surface paroccipital process:
(0) – straight
(1) – curving ventrally, pendant

This is equivalent to character 150 in our dataset, which concerns the absence/presence of a flange on the paroccipital process. It is the flange that causes the process to appear to curve ventrally as a pendant structure.

Character 176: Opisthotic, paroccipital process length:
(0) – elongate and slender
(1) – process short, thick, 60% or more of dorsoventral height compared to mediolateral length

Loewen et al. (2013) score all score-able tyrannosauroids for state 1, except for *Guanlong* and the two species of *Daspletosaurus*, which are scored for state 0. *Guanlong* has broken paroccipital processes and we have not been able to obtain an accurate measurement, and we have also been unable to confirm these measurements in *Daspletosaurus*. Additionally, the measurements of the paroccipital process are easily distorted during preservation.

Character 177: Opisthotic, exoccipital-opisthotic, paroccipital processes, deep fossa on posterior surface dorsolateral to the foramen magnum:
(0) – absent
(1) – present and shallow
(2) – present and deep

This is character 151 in our dataset, with an added intermediate state. Our character originally referred to the absence/presence of a fossa on the exoccipital-opisthotic. Loewen et al. (2013) have in effect divided the 'presence' condition into 'shallow' and 'deep' states. However, we are uncomfortable making such a distinction, because the difference between 'shallow' and 'deep' is not easily quantifiable, and the depth of the fossa can be easily affected by crushing. Therefore, we prefer a much simpler absence/presence character.

Character 179: Opisthotic, exoccipital-opisthotic, crista tuberalis (= metotic strut), extent in posterior view:
(0) – crista tuberalis absent or extent very limited
(1) – extensive, extends subequal to the length of the paroccipital process

This is character 152 in our dataset, redefined so that the crista tuberalis is measured relative to the length of the paroccipital process, instead of the depth of the braincase as in our original character. We prefer our original character wording, as in many specimens the paroccipital process is not completely preserved and therefore an accurate length measurement is not possible to obtain. In practice we find that braincase depth is more commonly measureable than paroccipital process length.

Character 180: Laterosphenoid, transverse scar on lateral surface:
(0) – absent
(1) – present

This character was originally used by Brusatte et al. (2009) but was excluded in our 2010 dataset. We reintroduce it here as character 365.

Character 181: Laterosphenoid, dorsal suture with the parietal:
(0) – flat
(1) – raised into a sharp ridge laterally

This character was originally used by Brusatte et al. (2009) but was excluded when we critically revised characters for the Brusatte et al. (2010) phylogenetic study, as we felt that the character was poorly defined and difficult to score in practice. We continue to exclude it here.

Character 187: Basioccipital, occipital condyle constriction of neck:
(0) – lateral constriction of neck
(1) – subspherical without constricted neck

State 1 is present in all tyrannosauroids, so it is not relevant for an in-group phylogenetic analysis.

Character 188: Basioccipital, subcondylar recesses:
(0) – absent
(1) – shallow
(2) – deep

This is character 156 in our dataset, with the ‘absent or shallow’ state in our character separated into ‘absent’ and ‘shallow’ states. Although in most cases we agree that mixing presence/absence information and variation in morphology within a single state is problematic, as outlined in Bever et al. (2013) the combination of ‘absence’ and ‘shallow’ into one state is a pragmatic decision, because oftentimes a very shallow recess may appear absent because of poor preservation.

Character 190: Basioccipital, basal tubera:
(0) – equally formed by basioccipital and basisphenoid and not subdivided
(1) – subdivided by a lateral longitudinal groove into a medial part entirely formed by the basioccipital, and a lateral part, entirely formed by the basisphenoid

State 0 is present in all tyrannosauroids, so it is not relevant for an in-group phylogenetic analysis.

Character 191: Basioccipital, basal tubera mediolateral width:
(0) – greater than occipital condyle width
(1) – equal to or less than occipital condyle width

State 0 is present in all tyrannosauroids, so it is not relevant for an in-group phylogenetic analysis.

Character 193: Basioccipital, basicranium, mediolateral width of basal tubera versus the width of the basispterygoid processes:
(0) – subequal or basal tuber wider
(1) – basipterygoids wider

We consider this character to be equivalent, or essentially so, with character 160 in our dataset, which refers to the width of the basipterygoid recess.

Character 195: Basioccipital, ventral surface across basal tubera and basisphenoids orientation:
(0) – oriented nearly horizontally
(1) – oriented caudoventrally

This character was originally used by Brusatte et al. (2009) but was excluded when we critically revised characters for the Brusatte et al. (2010) phylogenetic study, as we felt that the character was difficult to score consistently among specimens, particularly when there is distortion.We continue to exclude it here.

Character 196: Basioccipital, pneumatic openings associated with internal carotid artery:
(0) – absent
(1) – present

State 1 is present in all tyrannosauroids, so it is not relevant for an in-group phylogenetic analysis.

Character 197: Parabasisphenoid, with large surfaces of pronounced muscle scars flanking basisphenoid recess:
(0) – no
(1) – yes

This character was originally used by Brusatte et al. (2009) but was excluded when we critically revised characters for the Brusatte et al. (2010) phylogenetic study, as our comparative study of specimens revealed that the muscle scars (state 1) were present in all tyrannosauroids. Therefore, this character is not relevant for an in-group phylogenetic analysis. Loewen et al. (2013) score the scars as present in all tyrannosauroids except for *Xiongguanlong*, *Albertosaurus*, and *Gorgosaurus*, but pronounced scars are indeed visible in these taxa.

Character 199: Parabasisphenoid, basisphenoid recess:
(0) – undivided
(1) – divided

We do not understand this character, and are not sure what the ‘divided’ state refers to. Because this character is not clearly defined we exclude it.

Character 200: Parabasisphenoid, orientation of division of basisphenoid recess:
(0) – transversely divided so that there is an anterior and posterior recess
(1) – divided by a single midline strut into two laterally separate chambers
(2) – divided by a "Y" shaped strut of bone forming three recesses

We have not been able to confirm the scores provide by Loewen et al. (2013). We also note that these scores are distributed in something of a hodge-podge pattern across the tyrannosauroid phylogeny and do not have a clear phylogenetic signal (based on the topology generated from other characters), so we are concerned that these states are referring to random variation.

Character 202: Parabasisphenoid, basisphenoid recess, orientation of central axis:
(0) – horizontal, recess obscured in posterior view
(1) – posteroventral, recess partially visible in posterior view
(2) – extremely posteroventral, recess compressed anteroposteriorly and widely visible in posterior view, and basipterygoid processes located beneath the basal tubera

As cited by Loewen et al. (2013), we use this character as number 158 in our dataset. However we note here that we score *Bistahieversor* for state 1 whereas Loewen et al. (2013) score it for state 2.

Character 204: Palate, shelf-like primary palate, presence:
(0) – absent
(1) – present

We consider this to be equivalent to Loewen et al.'s (2013) character 44. See our comments regarding this character above.

Character 208: Palatine anterior expansion of dorsal process into a vomerine process:
(0) – absent
(1) – present

State 1 is present in all tyrannosauroids, so it is not relevant for an in-group phylogenetic analysis.

Character 210: Palatine posterior expansion of dorsal process:
(0) – absent
(1) – present

All score-able tyrannosauroids are scored for state 1, except for three taxa scored state 0, which we disagree with: *Appalachiosaurus* (the palatine is broken so the presence/absence of a posterior process cannot be determined with certainty, although based on the morphology of the broken surface we suspect that a posterior process was indeed present but is broken away), *Guanlong* (the dorsal portion of the palatine is broken in the specimen we examined, so we cannot determine a score with certainty), and *Proceratosaurus* (the vomeropterygoid process of the palatine is broken posteriorly so this character cannot be scored with certainty). Therefore, as

all score-able tyrannosauroids have state 1, this character is phylogenetically uninformative for an in-group analysis.

Character 212: Palatine, dorsoventral height of the dorsal process:
(0) – tall, 60% or more than narrowest rostroposterior width of the neck of the dorsal process
(1) – short, less than 50%

This character was originally used by Brusatte et al. (2009) but was excluded when we critically revised characters for the Brusatte et al. (2010) phylogenetic study, as we were concerned that it may be (at least partially) redundant with other characters in our dataset concerning the dorsal region of the palatine. We continue to exclude it here.

Character 213: Palatine, overall orientation of neck of dorsal process:
(0) – rostrodorsally directed at nearly 45 degrees
(1) – vertically directed

Although identified as a new character by Loewen et al. (2013), this is included in our dataset as character 135.

Character 214: Palatine, posterior expansion of base of the neck of the dorsal process:
(0) – absent
(1) – present

We do not understand this character. Loewen et al. (2013) score the posterior expansion (state 1) as present in *Alioramus altai*, *Tyrannosaurus*, and *Tarbosaurus* and absent in all other score-able tyrannosauroids. Based on our comparative study of tyrannosauroids we are not able to recognize a distinct, clearly defined posterior expansion in this region of the palatine in any tyrannosauroids.

Character 217: Palatine, position of the posterior edge of the posterior pneumatic recess compared to the posterior edge of the dorsal process:
(0) – anterior to the posterior margin of dorsal process
(1) – extends posterior to the posterior margin of the process

This character was originally used by Brusatte et al. (2009) but was excluded when we critically revised characters for the Brusatte et al. (2010) phylogenetic study, as we were concerned that it may be redundant with other characters in our dataset relating to the position of the palatine recess, particularly another character concerning the position of the posterior edge of the palatine pneumatic recess (character 138 in the current dataset). We continue to exclude it here.

Character 218: Palatine, presence of slot on the articular surface for the maxilla:
(0) – absent
(1) – present

This character appears in our dataset as character 141.

Character 219: Palatine, maxillary process, form of maxillary articulation:
(0) – flat
(1) – deeply excavated as a slot, demarcated dorsally by a pronounced lip of bone

This is identical to the preceding character in the Loewen et al. (2013) dataset, their character 218. We use this character as character 141 in our dataset (see preceding entry above).

Character 224: Epipterygoid ventral margin:
(0) – undivided
(1) – forked

This character was originally used by Brusatte et al. (2009) but was excluded when we critically revised characters for the Brusatte et al. (2010) phylogenetic study. As outlined in Brusatte et al. (2012), we have two issues with this character. First, *Tyrannosaurus* has both conditions so this character may be incredibly variable even within individual taxa, perhaps relating to ontogeny. Second, and more problematically, the forked condition (state 1) is often an artefact of breakage, as the ventral margin of the bone would have been straight in life but damage causes it to appear forked. This means it is very difficult to score this character confidently. We continue to exclude it here.

Character 230: Ectopterygoid, mediolateral length of jugal process:
(0) – short
(1) – long

This character was originally used by Brusatte et al. (2009) but was excluded when we critically revised characters for the Brusatte et al. (2010) phylogenetic study, as we could not identify a clear, quantitative difference between 'short' and 'long'. As Loewen et al. (2013) do not present such a quantitative distinction, we continue to exclude this character here.

Character 231: Ectopterygoid, dorsoventral thickening of jugal process:
(0) – thin jugal process
(1) – thickened

This character was originally used by Brusatte et al. (2009) but was excluded when we critically revised characters for the Brusatte et al. (2010) phylogenetic study, as we determined that the thickening of the jugal process (state 1) was largely related to whether or not the process is inflated by a pneumatic sinus, which is already denoted by a separate character (character 131 in our dataset, character 229 in Loewen et al. [2013]). We continue to exclude this character here.

Character 232: Ectopterygoid, dorsal recess form:
(0) – absent
(1) – present forms dorsal lip

State 0 is present in all tyrannosauroids, so it is not relevant for an in-group phylogenetic analysis. Additionally, as discussed by Gold et al. (2013), the 'dorsal recess' on the

ectopterygoid is a different type of ectopterygoid pneumaticity than the large recess that invades the bone in tyrannosauroids. The 'dorsal recess' is seen in other, non-tyrannosauroid theropods.

Character 233: Pterygoid, contact between pterygoid and palatine:
(0) – continuous
(1) – discontinuous in the mid-region, resulting in a subsidiary palatal fenestra

State 1 is present in all tyrannosauroids, so it is not relevant for an in-group phylogenetic analysis.

Character 236: Mandible, internal mandibular fenestra size:
(0) – small and slit-like
(1) – large and rounded

State 1 is present in all tyrannosauroids, so it is not relevant for an in-group phylogenetic analysis.

Character 237: Mandible, mandibular joint, position in lateral view:
(0) – anterior to or even with the paroccipital process
(1) – posterior to the paroccipital process

This character was originally used by Brusatte et al. (2009) but was excluded when we critically revised characters for the Brusatte et al. (2010) phylogenetic study, as we felt that it was at least partially redundant with other characters in our dataset relating to the orientation of the occipital surface of the skull (e.g., character 1), and also because it is difficult to score conclusively in some taxa because of deformation or incomplete preservation of the skull (to accurately score this character much of the skull and jaw in articulation need to be present). We still exclude here.

Character 239: Mandible, occlusion in lateral view:
(0) – jaws occlude for their full length
(1) – jaws diverge rostrally due to kink and downward deflection in dentary buccal margin

State 0 is present in all tyrannosauroids, so it is not relevant for an in-group phylogenetic analysis.

Character 240: Dentary, dorsal upturn of element at anterior end:
(0) – absent
(1) – present

State 0 is present in all tyrannosauroids, so it is not relevant for an in-group phylogenetic analysis.

Character 241: Dentary shape in lateral view:
(0) – with subparallel dorsal and ventral edges
(1) – subtriangular in lateral view, posteriorly expanded

State 1 is present in all tyrannosauroids, so it is not relevant for an in-group phylogenetic analysis.

Character 242: Dentary, expansion of posterior dentary:
(0) – posterior height of dentary two times or less than minimum dentary height
(1) – posterior end of dentary more than two times the minimum dentary height

We think that this character is partially redundant with other characters in our dataset, and Loewen et al.'s (2013) dataset, that relate to the proportions of the skull. Tyrannosauroids with a proportionally deep dentary are also those with a proportionally deep skull, a feature encapsulated in character 2 in our dataset and several characters (e.g., characters 3, 9, 11, 31) in the Loewen et al. (2013) dataset. See our commentary about these characters above.

Character 246: Dentary, ventral margin of bone in lateral view, profile:
(0) – straight
(1) – sigmoidal

Almost all score-able tyrannosauroids are scored for state 1 by Loewen et al. (2013), as the ventral margin of their dentary is concave or sigmoidal. Only *Dilong*, *Guanlong*, and *Eotyrannus* are scored for state 0 by Loewen et al. (2013), but these scores are problematic. *Dilong* has a ventral margin that is concave, not straight, so it should be scored for state 1. *Guanlong* is figured by Xu et al. (2006) as having a fairly straight ventral margin, but the specimen itself exhibits a concave margin. *Eotyrannus* appears to have a straight margin, but only the anterior region of the dentary is preserved, so it is unclear what shape the entire margin would have taken (indeed, often the anterior portion of the dentaries of tyrannosauroids have a straight ventral margin but a concave/sigmoidal ventral margin).

Character 247: Dentary, posterior end, presence of posterodorsal process above external mandibular fenestra:
(0) – present
(1) – absent

This character is in reference to the forking of the dentary posteriorly, as the dorsal tine of the fork is the posterodorsal process that extends above the external mandibular fenestra. Loewen et al. (2013) score all score-able tyrannosauroids for state 0, except for a variety of derived tyrannosaurids (albertosaurines, tyrannosaurines, and *Bistahieversor*), which are scored as lacking the process (state 1). We disagree with these scores, as these derived tyrannosauroids possess a posterodorsal process of the dentary that helps define the external mandibular fenestra (e.g., Brochu 2003; Currie 2003), although this process is small and sometimes can be broken so that it appears absent. We are unaware of any tyrannosauroid specimens that definitively lack the posterodorsal process, so therefore all score-able taxa are scored for state 0, making this character uninformative in a tyrannosauroid ingroup analysis.

Character 248: Dentary, posterior end, presence of a surangular process on the dorsal surface of the mandible:
(0) – absent

(1) – present

State 1 is present in all tyrannosauroids, so it is not relevant for an in-group phylogenetic analysis.

Character 250: Dentary, position of Meckelian groove:
(0) – closer to ventral margin of bone
(1) – roughly at midheight

This character was originally used by Brusatte et al. (2009) but was excluded when we critically revised characters for the Brusatte et al. (2010) phylogenetic study, as we found it to be redundant with another character in our dataset (character 178, used as character 249 by Loewen et al. [2013]), which concerns the dorsoventral depth and inset of the Meckelian groove. All tyrannosauroids with a narrow and deeply inset groove have the groove positioned at roughly midheight of the dentary. We argue that the midheight position of the groove is a consequence of its reduction from a very dorsoventrally deep (and shallowly inset) structure in the ancestors of tyrannosauroids into the very dorsoventrally shallow (and deeply inset) groove of tyrannosauroids. As the groove becomes very dorsoventrally shallow, it can only occupy a limited region of the bone, which in tyrannosauroids is around midheight of the dentary. If a tyrannosauroid was discovered with a dorsoventrally shallow/deeply inset groove that is not positioned at midheight of the bone, then we would reintroduce this character.

Character 251: Dentary, anterior extent of lingual bar:
(0) – medial to first alveolus
(1) – medial to second (or further posterior) alveolus

This character was originally used by Brusatte et al. (2009) but was excluded when we critically revised characters for the Brusatte et al. (2010) phylogenetic study, as we were unable to consistently score it among taxa. We continue to exclude it here.

Character 255: Dentary, distinct interdental plates:
(0) – present
(1) – absent

State 0 is present in all tyrannosauroids, so it is not relevant for an in-group phylogenetic analysis.

Character 258: Splenial, anterior margin:
(0) – tapered point
(1) – squared or jagged anterior end

We do not understand this character. Loewen et al. (2013) scores all score-able tyrannosauroids for state 1, except for *Tyrannosaurus*, *Tarbosaurus*, and *Bistahieversor*, which are scored for state 0. We do not recognize a clear, discrete, defined difference between the condition in, for example, *Alioramus altai* (state 1) and *Tyrannosaurus* (state 0), as in both taxa the process

tapers. We find that there is no clearly defined distinction among tyrannosauroids and exclude the character here.

Character 259: Splenial, form of posterior end:
(0) – straight or slightly curved
(1) – distinctly forked

State 1 is present in all tyrannosauroids, so it is not relevant for an in-group phylogenetic analysis.

Character 260: Splenial, mylohyoid foramen:
(0) – small, smaller than dentary alveoli
(1) – large, subequal to or larger than dentary alveoli

This is a modified version of our character 191, with comparisons to the dentary alveoli used to quantify the size of the foramen rather than comparisons to the remainder of the splenial as we originally used. We prefer to retain our character language, and note that the redefinitions of Loewen et al. (2013) render this character uninformative, as now all score-able tyrannosauroids are scored for state 1.

Character 261: Splenial, myohyoid foramen orientation of long axis:
(0) – horizontal
(1) – dorsoposteriorly inclined in line with intercoronoid process

This is cited by Loewen et al. (2013) as a modified version of our character 191. However, our character does not concern the orientation of the foramen, only its size and shape. Loewen et al. (2013) score basal tyrannosaurois, alioramins, and albertosaurines for state 0 (a horizontal foramen) and tyrannosaurines and *Bistahieversor* for state 1 (an inclined foramen). We do not recognize a clear distinction between these taxa. For example, as figured and described by Brusatte et al. (2012), *Alioramus altai* has a formaen with a long axis that is posterodorsally inclined, not horizontal, but this is contra its score of state 0 by Loewen et al. (2013). Similarly, Loewen et al. (2013) score *Appalachiosaurus* for state 0, but Carr et al. (2005) figures this taxon as possessing an inclined foramen (state 1). As we see no clear, discrete, easy-to-define difference among tyrannosauroids in the orientation of the foramen, we exclude this character here.

Character 262: Splenial, mediolateral thickness of intercoronoid process:
(0) – no thickening
(1) – thickened mediolaterally

We have not been able to find a clear way to quantify the difference between ‘no thickening’ and ‘thickened’. As Loewen et al. (2013) did not provide a quantitative distinction, we exclude this character here.

Character 263: Angular, exposure in lateral view:
(0) – exposed almost to end of mandible in lateral view, reaches or almost reaches articular

(1) – excluded from posterior end

State 1 is present in all tyrannosauroids, so it is not relevant for an in-group phylogenetic analysis.

Character 264: Angular, exclusion from posterior end:
(0) – end of angular stops well short of glenoid
(1) – stops near the anterior end of glenoid

State 1 is present in all tyrannosauroids, so it is not relevant for an in-group phylogenetic analysis.

Character 265: Angular posterior process:
(0) – posterior process point projects dorsally above ventral margin of mandible
(1) – posterior process tip flush with ventral margin of mandible

State 1 is present in all tyrannosauroids, so it is not relevant for an in-group phylogenetic analysis.

Character 267: Surangular, dorsoventral depth:
(0) – shallow, element less than 35% craniocaudal length
(1) – deep, depth 40% or more element craniocaudal length

We think that this character is partially redundant with other characters in our dataset, and Loewen et al.'s (2013) dataset, that relate to the proportions of the skull. Tyrannosauroids with a proportionally deep surangular are also those with a proportionally deep skull, a feature encapsulated in character 2 in our dataset and several characters (e.g., characters 3, 9, 11, 31, 242) in the Loewen et al. (2013) dataset. See our commentary about these characters above.

Character 268: Surangular, lateral surface, wide depression present for dorsal surangular process of the dentary:
(0) – absent or narrow groove
(1) – wide depression

State 1 is present in all tyrannosauroids, so it is not relevant for an in-group phylogenetic analysis.

Character 269: Surangular, groove on dorsolateral surface of anterior end:
(0) – absent
(1) – present, shallow groove faintly visible
(2) – present, deep well-demarked groove

We do not understand this character. Loewen et al. (2013) score most tyrannosauroids for state 1, but consider state 2 to be present in *Bistahieversor*, *Lythronax*, *Tyrannosaurus*, and *Tarbosaurus*. We do not see a clear, discrete, well-defined difference between the groove on the lateral surface of the surangular in, for example, *Alioramus altai* (scored 1) and *Tyrannosaurus* (scored 2). The

only clear distinction we see is between the absence and presence of the groove. All score-able tyrannosauroids have the groove, so therefore we judge this character to be uninformative for an in-group analysis. In the absence of a clear way to quantify ‘shallow, faintly visible’ and ‘well-demarcated’, we excluded this character here.

Character 270: Surangular, foramen in dorsolateral groove:
(0) – absent
(1) – present, small foramen much smaller than groove
(2) – present, large foramen subequal to groove in diameter

We feel the same way about this character as we do the preceding character in the Loewen et al. (2013) dataset. We do not see any clear, discrete distinction between the size of the foramen in taxa scored for state 1 (e.g., *Alioramus altai*) and those scored for state 2 (e.g., *Tyrannosaurus*). All tyrannosauroids have a fairly large and distinctive foramen within the groove, and we find that the proper distinction is between the absence/presence of the foramen, with no consistent way to score differences in the size of the foramen.

Character 271: Surangular, anteroventral extension:
(0) – extension is absent
(1) – encloses external mandibular fenestra by contacting the angular anteriorly

State 0 is present in all tyrannosauroids, so it is not relevant for an in-group phylogenetic analysis.

Character 273: Surangular, surangular shelf, orientation relative to the long axis of the lower jaw:
(0) – ventrally inclined anteriorly or sigmoidal
(1) – dorsally inclined anteriorly
(2) – horizontal

This is a modified version of character 182 in our dataset. Loewen et al. (2013) rescore *Alioramus remotus* and *Alioramus altai* for state 1, whereas we score them for state 2. These taxa should be scored for state 2 (Brusatte et al. 2012).

Character 278: Surangular, laterally inclined flange on caudodorsal surface for articulation with lateral process of lateral quadrate condyle:
(0) – absent
(1) – present

State 0 is present in all tyrannosauroids, so it is not relevant for an in-group phylogenetic analysis.

Character 279: Prearticular shape:
(0) – dorsally concave sweeping low curve in lateral view
(1) – subcircular in lateral view, “U” shaped

We judge this character to be partially redundant with other characters in our dataset, and Loewen et al.'s (2013) dataset, that relate to the proportions of the skull. Tyrannosauroids that are scored for state 0 ('sweeping low curve'), such as alioramins and basal tyrannosauroids, have proportionally long and low skulls. Taxa scored for state 1 ('U-shaped'), such as albertosaurines and tyrannosaurines, have deeper skulls. Taxa with a deeper skull are going to have a deeper and more U-shaped prearticular, because this bone stretches from the posteroventral corner of the jaw to the dorsal margin of the jaw, so it therefore occupies the entire depth of the jaw. We thus think that this is another character that is essentially capturing skull proportions, which are already encapsulated in character 2 in our dataset and several characters (e.g., characters 3, 9, 11, 31, 242, 267) in the Loewen et al. (2013) dataset. See our commentary about these characters above.

Character 280: Prearticular, anterior process expansion:
(0) – unexpanded
(1) – expanded into a paddle like shape, more than 220% the dorsoventral depth of the midshaft

State 0 is present in all tyrannosauroids, so it is not relevant for an in-group phylogenetic analysis.

Character 282: Prearticular, ventral process or flange:
(0) – absent
(1) – present

Loewen et al. (2013) score the flange as present in *Bistahieversor*, *Lythronax*, and *Tyrannosaurus*, and absent in all other score-able taxa as lacking the flange. Based on our comparative study of tyrannosauroids, we are not confident that the flange is a genuine feature that is consistently present or absent in taxa. For example, there is a small ventral flange on the right prearticular of the *Tyrannosaurus* specimen FMNH PR 2081, but it does not appear to be present on the left side of the same individual (Brochu 2003). Additionally, Loewen et al. (2013) consider the flange as absent in *Tarbosaurus*, but there is a subtle projection on at least one specimen (ZPAL MgD-I/34). We are still not clear if this flange is a distinct, discrete structure or a subtle feature that is variably expressed (perhaps because it is a muscle or ligament attachment site, and does not always ossify). In practice we find that this feature is difficult to consistently score and is often difficult to see because of damage or poor preservation, or in the case of photographs, the orientation the photograph was taken at. For these reasons we do not feel like we can confidently score this character, so we exclude it.

Character 284: Prearticular, shape of caudoventral margin:
(0) – sweeping ventrally convex curve
(1) – sigmoidal with downturned articular process

Loewen et al. (2013) score all score-able tyrannosauroids for state 1, with the exception of *Dilong*, *Guanlong*, and *Raptorex*, which are scored for state 0. *Raptorex* is broken in this region so it cannot be confidently scored. The specimens of *Guanlong* and *Dilong* that we have studied cannot be observed in medial view, so we have been unable to confirm these character scores. Therefore, all tyrannosauroids that we can score possess state 1, making this character uninformative in an in-group analysis.

Character 285: Articular, medial process:
(0) – absent
(1) – present

Loewen et al. (2013) score all score-able tyrannosauroids for state 1, except for *Proceratosaurus* and *Dilong*, which are scored for state 0. *Proceratosaurus* should be scored 1, as Rauhut et al. (2010) describe a large medial process on the articular, which is confirmed by our study of the specimen. We are unable to score *Dilong* because this portion of the articular is obscured. Therefore, all tyrannosauroids that we can score possess state 1, making this character uninformative in an in-group analysis.

Character 286: Articular, retroarticular process shape:
(0) –rod-like, sort
(1) – rod-like, elongate and slender
(2) – short, wide, and with distinct posteriorly facing concavity

State 2 is present in all tyrannosauroids, so it is not relevant for an in-group phylogenetic analysis.

Character 288: Articular, pneumatic foramen posterior to glenoid:
(0) – absent
(1) – present

State 1 is present in all tyrannosauroids, so it is not relevant for an in-group phylogenetic analysis.

Character 289: Premaxilla, number of teeth:
(0) – 5 or more
(1) – 4
(2) – 3
(3) – edentulous

State 1 is present in all tyrannosauroids, so it is not relevant for an in-group phylogenetic analysis.

Character 294: Premaxillary teeth, presence of serrations:
(0) – present
(1) – absent on at least the mesial carina or completely absent

State 0 is present in all tyrannosauroids, so it is not relevant for an in-group phylogenetic analysis.

Character 295: Maxillary teeth, presence:
(0) – present
(1) – absent edentulous

State 0 is present in all tyrannosauroids, so it is not relevant for an in-group phylogenetic analysis.

Character 297: Maxilla, position of the posteriormost tooth:
(0) – nearer to the posterior end of the antorbital fenestra
(1) – posterior to but closer to the midpoint of the antorbital fenestra
(2) – anterior to the midpoint of the antorbital fenestra

Based on our comparative study of tyrannosauroids, we find that all score-able taxa have state 1, with the exception of *Proceratosaurus*, which has state 0. State 0 is currently an autapomorphy of a single tyrannosauroid taxon. Currently, this character is uninformative in an in-group analysis (state 0 is seen in a few outgroups, but various tyrannosauroid outgroups have states 0, 1, and 2, so it is not clear that state 0 is the ancestral condition for tyrannosauroids being retained by *Proceratosaurus* and state 1 is a synapomorphy linking all more derived tyrannosauroids, in which case this character would be informative). In the event that additional tyrannosauroids are found with state 0, we will reintroduce this character into the dataset.

Character 299: Dentary, portion of dentary with teeth:
(0) – teeth throughout to 65% of the dorsal surface of the dentary
(1) – teeth restricted to the rostral 33% or edentulous

State 0 is present in all tyrannosauroids, so it is not relevant for an in-group phylogenetic analysis.

Character 300: Dentary, number of teeth:
(0) – 22 or more alveoli
(1) – 18 to 21 alveoli
(2) – 15-17 alveoli
(3) – 14 or less alveoli

We added a similar character to our dataset (character 315) when it was modified for the Lü et al. (2014) description of *Qianzhousaurus*.

Character 301: Tooth spacing on maxilla and dentary:
(0) – closely appressed
(1) – widely spaced with considerable separation between alveoli

State 0 is present in all tyrannosauroids, so it is not relevant for an in-group phylogenetic analysis.

Character 304: Axis, pleurocoel:
(0) – absent
(1) – present

State 1 is present in all tyrannosauroids, so it is not relevant for an in-group phylogenetic analysis.

<u>Character 309</u>: Axis, axial parapophyses and diapophyses:
(0) – moderate/prominent
(1) – reduced/absent

State 0 is present in all tyrannosauroids, so it is not relevant for an in-group phylogenetic analysis.

<u>Character 311</u>: Axis, neural spine in cranial view:
(0) – flared transversely
(1) – compressed mediolaterally

Loewen et al. (2013) scores most tyrannosauroids for state 0, except for *Raptorex*, *Dilong*, and *Guanlong*. This character has a long legacy in phylogenetic analyses of coelurosaurs as a whole, and has been included in several iterations of the TWiG dataset. In the most recent version, Brusatte et al. (2014) scores all tyrannosauroids for state 0, including *Dilong* (*Guanlong* is scored as '?' and *Raptorex* is not included in their dataset). We agree that all tyrannosauroids exhibit the same state, state 0, so this character is uninformative for an in-group analysis.

<u>Character 314</u>: Axis, epineurapohyseal processes on axis neural spine:
(0) – absent
(1) – present

Loewen et al. (2013) scores all score-able tyrannosauroids for state 1, except for *Guanlong*, which is scored for state 0. We have not been able to confirm this score for *Guanlong* (as we have not been able to observe an axis for this taxon), so we must consider it '?'. Therefore, in our analysis all score-able tyrannosauroids have state 1, so this character is uninformative.

<u>Character 315</u>: Axis, epipophyses, posterior extent:
(0) – absent or terminates well anterior to postzygapophysis
(1) – extends to, or only slightly posterior to, the posterior margin of the postzygapophysis
(2) – large, rugose flange that extends posterior to postzygapophysis
(3) – elongate flange that extends posteriorly 75% or more than the anteriorposterior length of the postzygapophysis

This is a modified version of character 210 in our dataset, with two extra states added (state 0 and state 3 here). We prefer our simpler binary character, which differentiates between a small epipophysis and one that is large and flange-like. We are uncomfortable dividing this character into several states, atomizing it to an extent that the boundaries between adjacent states are very subtle.

<u>Character 316</u>: Cervical vertebrae, number of pneumatic openings in anterior centra:
(0) – one pair of pneumatic openings
(1) – two pairs of pneumatic openings

Loewen et al. (2013) score all score-able tyrannosauroids for state 0, with the exception of *Xiongguanlong* and *Raptorex*, which are scored for state 1. State 1 (two pneumatic openings) is genuinely present in *Xiongguanlong*, but *Raptorex* is correctly scored for state 0, as it has a single foramen on the side of each cervical (Sereno et al. 2009). Therefore, state 1 is an autapomorphy for *Xiongguanlong* among tyrannosauroids, making this character uninformative for an in-group analysis. In the event that another tyrannosauroid with two pneumatic openings on the side of each cervical is discovered, we will reintroduce this character into the dataset.

Character 317: Cervical vertebrae, form of centra:
(0) – amphiceolous to platycoelous
(1) – strongly opisthocoelous

State 0 is present in all tyrannosauroids, so it is not relevant for an in-group phylogenetic analysis.

Character 318: Cervical vertebrae, epipophyses of C3-5, form:
(0) – absent or terminates well anterior to postzygapophysis
(1) – extends to, or only slightly posterior to, the posterior margin of the postzygapophysis
(2) – large, rugose flange that extends posterior to postzygapophysis

This is a modified version of character 210 in our dataset, which concerns the epipophyses on both the axis and the anterior-middle postaxial cervical vertebrae. Loewen et al. (2013) divides this character into two: one for the axis (character 315) and the other for the postaxial cervicals (character 318, the current character). All tyrannosauroids that can be scored affirmatively are scored identically for these two characters. Therefore, we exclude this second character in our dataset, out of concern that it is encapsulating a serially homologous structure, and in effect weighing this character double by including it twice. If a tyrannosauroid is found with different epipophysis morphology in the axis and postaxial cervicals, then we will reintroduce this second character into the dataset.

Character 319: Cervical vertebrae, position of posterior edge of anterior cervical centra:
(0) – level with or anterior to the posterior extent of neural arch
(1) – extending beyond posterior limit of neural arch

Loewen et al. (2013) scores all score-able tyrannosauroids for state 0, except for *Guanlong*, which is scored as state 1. This character is used often in phylogenetic analyses of theropods, including the TWiG analysis of coelurosaurs. In the most recent version of the TWiG dataset (Brusatte et al. 2014), *Guanlong* is scored for state 0, meaning that all tyrannosauroids are scored the same, making this character uninformative for an in-group analysis. We agree that *Guanlong* should be scored for state 0.

Character 320: Cervical vertebrae, anterior cervical centra, shape in anterior view:
(0) – subcircular or square
(1) – distinctly wider than high; kidney shaped

State 0 is present in all tyrannosauroids, so it is not relevant for an in-group phylogenetic analysis.

Character 321: Cervical vertebrae, anteroposterior length of centrum compared to dorsoventral height of posterior centrum face:
(0) – longer than tall
(1) – shorter than tall, or subequal

This is a modified version of character 202 in our dataset, which concerns the proportions of both the axis and the anterior-middle postaxial cervical vertebrae. Loewen et al. (2013) divides this character into two: one for the axis (character 307) and the other for the postaxial cervicals (character 321, the current character). All tyrannosauroids that can be scored affirmatively are scored identically for these two characters. Therefore, we exclude this second character in our dataset, out of concern that it is encapsulating a serially homologous structure, and in effect weighing this character double by including it twice. If a tyrannosauroid is found with different centrum proportion morphologies in the axis and postaxial cervicals, then we will reintroduce this second character into the dataset. Our rationale here is the same as our rationale for excluding character 318 of Loewen et al. (2013).

Character 322: Cervical vertebrae, neural spine positon:
(0) – centroid of neural spine positioned on anterior half of vertebrae
(1) – centroid of neural spine positioned posterior to the midpoint of vertebrae

Loewen et al. (2013) scores all score-able tyrannosauroids for state 1, except for *Guanlong*, which is scored for state 0. We do not recognize any distinct difference between the positions of the cervical neural spines in *Guanlong* and other tyrannosauroids. The neural spines of large derived tyrannosauroids, such as *Tyrannosaurus*, do appear to be positioned very far posteriorly relative to the centrum, but this is due to the fact that the centra become proportionally short, which is already encapsulated by character 202 in our dataset. If there are any differences among tyrannosauroids in this character, they are most likely related to differences in overall cervical vertebrae proportions.

Character 323: Cervical vertebrae, neural spine anterioposterior minimum width:
(0) – wide, ½ or more than the length of the centrum
(1) – narrow, less than ½ the length of the centrum

This is cited as a new character by Loewen et al. (2013), but it is essentially equivalent to character 211 in our dataset. Taxa are scored nearly identically for our character and Loewen et al.'s character, supporting their synonymy.

Character 324: Cervical vertebrae, neural spine dorsoventral height:
(0) – short, less than the height of the centrum
(1) – low and centered on neural arch, giving arch an "X" shape in dorsal view
(2) – approximately the same height as the posterior centrum face
(3) – tall, 1.5 or more than the height of the centrum face

This is a modified version of character 212 in our dataset, in which our state 1 is divided into two states (states 2 and 3 here). Loewen et al. (2013) score *Teratophoneus, Bistahieversor, Tyrannosaurus*, and *Tarbosaurus* for their state 3, but we have an issue with some of these scores. For example, *Tyrannosaurus* does not possess cervical neural spines that are taller than 1.5 times the height of the centrum face (Brochu 2003). Therefore, we believe that this character is being over-divided into states that cannot be clearly and consistently scored. We hold that the major difference between taxa is whether they possess spines that are much shorter than the centrum or spines that are about as tall as the centrum or a little taller. We thus retain our original character usage.

<u>Character 325</u>: Cervical vertebrae, neural spine mediolateral thickness:
(0) – thin
(1) – anteroposterally tapering thin to thick
(2) – thick, stout rectangle, base is nearly square with nearly equal anteroposterior and transverse dimensions

This character is cited as being modified from character 211 in our dataset. Loewen et al. (2013) have divided our character 211 into two characters: one relating to the anteroposterior length of the neural spine in dorsal view (their character 323) and one relating to the mediolateral width of the spine in dorsal view (their character 325, the current character). The two characters are not scored identically, but nearly so. We think that these two characters are basically referring to the same general trend in tyrannosauroids: the progressive phylogenetic change from an anteroposteirorly elongate, mediolaterally thin, sheet-like neural spine to a stouter spine with a nearly square cross section. Therefore, we retain only a single character to encapsulate this: our character 211.

<u>Character 326</u>: Cervical vertebrae, rugose ligament attachment scars on anterior and posterior surfaces of neural spine:
(0) – absent or weakly developed
(1) – present as prominent, flanges visible in lateral view

This is a modified version of character 217 in our dataset, which concerns the ligament attachment scares of both the cervical and dorsal vertebrae. Loewen et al. (2013) divides this character into two: one for the cervicals (character 326, the current character) and the other for the dorsals (character 335). We prefer to use a single character, out of concern that it is encapsulating a serially homologous structure, and in effect weighing this character double by including it twice. Our rationale here is the same as our rationale for excluding characters 318 and 321 of Loewen et al. (2013).

<u>Character 327</u>: Cervical vertebrae, orientation of posterior centrodiapophyseal lamina in C3-5:
(0) – nearly horizontal, fossa located primarily dorsal to lamina
(1) – projects posteroventrally, infrapostzygapophyseal fossa located primarily posterior to lamina

This is a modified version of character 216 in our dataset, but *Alioramus altai*, *Xiongguanlong*, and *Raptorex* have been rescored so that they are now scored for a ‘horizontal’ fossa and not the

‘posteroventrally projecting’ fossa like in our original dataset. We retain our original scorings. *Alioramus altai*, for example, has the posteroventrally oriented posterior centrodiapophyseal laimna that is typical of theropods (Brusatte et al. 2012), not the ‘horizontally’ oriented lamina that is seen in some derived tyrannosaurids (e.g., *Tyrannosaurus*: Brochu 2003).

Character 329: Dorsal centra pneumaticity:
(0) – apneumatic
(1) – pneumaticity limited to anterior dorsals
(2) – present throughout dorsal column

This is a modified version of character 218 in our dataset, with our ‘absent or limited to posterior dorsals’ character divided into two states. Loewen et al. (2013) score *Guanlong* as the only tyrannosauroid for their new state ‘apneumatic’, but this taxon does have small pneumatic foramina on dorsals 2 and 3. Therefore, there are no tyrannosauroids currently known that completely lack dorsal vertebrae pneumaticity, so we retain our original character wording. If such an apneumatic tyrannosauroid is found, we will introduce an ‘apneumatic’ character state into our dataset.

Character 331: Dorsal vertebrae, anterior dorsal neural spines, level of posterior termination:
(0) – at approximately the same level as the posterior centrum face
(1) – far posterior to the posterior centrum face

Character 332: Dorsal vertebrae, mid- posterior dorsal neural spines, level of posterior termination:
(0) – at approximately the same level as the posterior centrum face
(1) – far posterior to the posterior centrum face

Loewen et al. (2013) divided our character 219 into separate characters relating to the anterior dorsal vertebrae (character 331) and the mid-posterior dorsal vertebrae (character 332). Based on our comparative studies of tyrannosauroids, all taxa should have the same scores for each character (i.e., if they have state 0 for character 331 they should have state 0 for character 332). This is contra Loewen et al. (2013), who score some taxa differently for the two characters (for example, score *Xiongguanlong* and *Tyrannosaurus* 1 for character 331 and 0 for 332, whereas they both should be scored 0 for both characters). Therefore, we consider these two characters to be redundant with each other, and do not elect to split them into two, because of concerns about serial homology. We retain our original use of one character referring to all dorsal vertebrae.

Character 333: Dorsal vertebrae, anterioposterior minimum width of mid-dorsal neural spine in lateral view:
(0) – wide, subequal to centrum length
(1) – narrow, ½ centrum length or less

We have been unable to confirm the character scores in Loewen et al. (2013) based on our own measurements of specimens. Therefore, we conservatively exclude this character, but note that we may reintroduce it at a later time if we can recognize clear quantitative differences among taxa.

Character 334: Dorsal vertebrae, dorsoventral height of mid-dorsal neural spine:
(0) – short, subequal to or less than centrum height
(1) – tall, 1.5 x centrum height or more

We exclude this character for two reasons. First, we already use a character relating to the cervical vertebrae (character 212 in our dataset) that is very similar to this character, and we are concerned about serial homology. Second, we do not understand state 1, which is scored as present in *Xiongguanlong* and more derived tyrannosauroids (e.g., *Tyrannosaurus*). In taxa like *Tyrannosaurus* the spine is not 1.5 times the height of the centrum or greater (e.g., Brochu 2003: figure 54). The spine and the centrum are approximately equal in height, with the spine at most slightly deeper. This is also the case, for example, in *Guanlong*, which is scored for state 0 by Loewen et al. (2013). Other taxa scored as 0 by Loewen et al. (2013) are also problematic: *Dilong* (most of the dorsals are only visible in dorsal view so the centrum cannot be measured, but the one centrum that can be measured is approximately the same height as the adjacent neural spine) and *Juratyrant* (no dorsal neural spine is complete, but the preserved portions of the spines indicate that they are most likely about the same height as the centrum). In other words, *Guanlong*, *Dilong*, and *Juratyrant* appear to have the same condition as taxa like *Tyrannosaurus*. Therefore, we do not find any major distinction in spine-to-centrum ratios among tyrannosauroids.

Character 335: Dorsal vertebrae, rugose ligament attachment scars on anterior and posterior surfaces of neural spine:
(0) – absent or weakly developed
(1) – present as prominent, flanges visible in lateral view

See our comments about Loewen et al.'s (2013) character 326 above.

Character 336: Neural spines on posterior dorsal vertebrae, shape in lateral view:
(0) – rectangular or square
(1) – anteroposteriorly expanded distally, fan-shaped

State 0 is present in all tyrannosauroids, so it is not relevant for an in-group phylogenetic analysis.

Character 337: Ventral keel in anterior dorsals:
(0) – absent or very poorly developed
(1) – pronounced

All score-able tyrannosauroids are scored for state 1, except for *Juratyrant*, which is scored for state 0. Only a single anterior dorsal vertebra is known for *Juratyrant*, and it is poorly preserved (Benson 2008). We are not confident that we can score this specimen accurately. Even if *Juratyrant* genuinely possesses state 0, this would be an autapomorphy among tyrannosauroids, and this character would be phylogenetically uninformative for an in-group analysis.

Character 338: Posteriormost dorsals, parapophyses position:

(0) – on the same level as transverse process
(1) – distinctly below transverse process

State 1 is present in all tyrannosauroids, so it is not relevant for an in-group phylogenetic analysis.

<u>Character 339</u>: Sacral vertebrae, number of sacral vertebrae:
(0) – 5 or less
(1) – 6

State 0 is present in all tyrannosauroids, so it is not relevant for an in-group phylogenetic analysis.

<u>Character 340</u>: Sacral vertebrae, length of anterior sacrals and posterior dorsal centrum length:
(0) – subequal in length
(1) – sacrals substantively longer than posterior dorsal centra.

There is variation among tyrannosauroids in this character, as taxa like *Guanlong* and *Juratyrant* have state 0 and taxa like *Alioramus altai* and *Tyrannosaurus* have state 1. However, we are concerned that this variation is related to the anteroposterior shortening of the dorsals in derived tyrannosauroids, which is encapsulated in character 220 in our dataset, and character 330 in the Loewen et al. (2013) dataset). We therefore conservatively exclude this character.

<u>Character 341</u>: Sacral vertebrae, pleurocoels or foramina on lateral surfaces of centra:
(0) – absent on sacral vertebrae
(1) – present on anterior sacrals only

We note that Loewen et al. (2013) is more liberal in giving affirmative (0 or 1) scores to taxa than we are. We retain our original scores because: 1) we wish to be careful when scoring based on specimens that are poorly preserved (e.g., *Alioramus*, which we score as '?' but Loewen et al. [2013] score as 1), and 2) we are conservative in scoring for pneumaticity only when there are unequivocal indicators present (e.g., smooth-walled external foramina leading into an internal cavity: Britt 1993; Benson et al. 2012).

<u>Character 346</u>: Caudal vertebrae: presence of transverse processes:
(0) – on more than 16 posteriors
(1) – on 12 to 16
(2) – on fewer than 12 posteriors

This character was originally used by Brusatte et al. (2009) but was excluded when we critically revised characters for the Brusatte et al. (2010) phylogenetic study, as we found it difficult to confidently recognize any clear differences among tyrannosauroids, as a complete tail is needed to be certain of the score. We note that two characters of a similar nature are present in the TWiG coelurosaur dataset (Brusatte et al. 2014: characters 113, 114) and all score-able tyrannosauroids are scored identically for both of these characters, indicating that there is no large-scale variation among tyrannosauroids.

Character 347: Caudal vertebrae, transition point in caudal series at which transverse processes end:
(0) – begins distal to the 10th posterior
(1) – between the 5th and 10th posterior vertebra

State 0 is present in all tyrannosauroids, so it is not relevant for an in-group phylogenetic analysis.

Character 348: Caudal vertebrae, anterior caudal centra shape:
(0) – tall, oval or round in cross section
(1) – with box-like centra in posteriors I-V

State 0 is present in all tyrannosauroids, so it is not relevant for an in-group phylogenetic analysis.

Character 351: Caudal vertebrae, anterior caudals, two laminae linking prezygapophysis and transverse process, between which is a triangular fossa:
(0) – absent
(1) – present, shallow fossa
(2) – present, deep fossa

This is a modified version of character 231 in our dataset, in which our character state scoring for the presence of the fossa is divided into separate states for 'shallow' and 'deep' fossae. We cannot recognize any clear, distinct, easy-to-define difference between taxa scored for a 'shallow' fossa (e.g., *Alioramus altai*) and those scored for a 'deep' fossa (e.g., *Tyrannosaurus*). We are hesitant to over-atomize this character and, because of the lack of any quantitative distinction between 'shallow' and 'deep', we prefer to retain our original character that differentiates simply between the presence and absence of the fossa.

Character 352: Caudal vertebrae, neural spines:
(0) – simple, undivided
(1) – separated into anterior and posterior alae throughout much of caudal sequence

State 0 is present in all tyrannosauroids, so it is not relevant for an in-group phylogenetic analysis.

Character 353: Caudal vertebrae, anterior spur in front of neural spine in mid-caudals:
(0) – absent
(1) – present

State 0 is present in all tyrannosauroids, so it is not relevant for an in-group phylogenetic analysis.

Character 354: Caudal vertebrae, distal caudal neural spine shape:
(0) – form a low ridge

(1) – spine absent

State 0 is present in all tyrannosauroids, so it is not relevant for an in-group phylogenetic analysis.

Character 355: Caudal vertebrae, mid to distal caudal postzygapophyses length:
(0) – short between 20% to 100% centrum length
(1) – extremely long, between 150% to 1000% centrum length

State 0 is present in all tyrannosauroids, so it is not relevant for an in-group phylogenetic analysis.

Character 356: Chevrons, anterior process of chevron base:
(0) – absent/weak
(1) – large

State 1 is present in all tyrannosauroids, so it is not relevant for an in-group phylogenetic analysis.

Character 357: Chevrons of anterior caudals, form of distal end:
(0) – short anteroposteriorly, shaft cylindrical
(1) – distal end elongate anteroposteriorly, flattened and plate-like

State 0 is present in all tyrannosauroids, so it is not relevant for an in-group phylogenetic analysis.

Character 358: Chevrons, form of mid-caudal chevrons:
(0) – rod-like or only slightly expanded ventrally
(1) – L-shaped

All tyrannosauroids except for *Bagaraatan* are scored for state 1. We do not include *Bagaraatan* in our dataset because of concerns that it is a chimaera (see Brusatte 2013). Therefore, all tyrannosauroids in our dataset are scored for state 1, making this character is uninformative.

Character 359: Chevrons, form of distal caudal chevrons:
(0) – rod-like or L-shaped
(1) – skid-like or T-shaped

All tyrannosauroids except for *Bagaraatan* and *Dilong* are scored for state 1. See comments on *Bagaraatan* above. *Dilong* is scored incorrectly: it possesses skid-like/T-shaped chevrons (Xu et al. 2004). Therefore, all tyrannosauroids in our dataset are scored for state 1, making this character uninformative.

Character 360: Pneumatic excavations in cervical or dorsal rib heads:
(0) – absent
(1) – present

State 1 is present in all tyrannosauroids, so it is not relevant for an in-group phylogenetic analysis.

Character 361: Scapula, length relative to humerus:
(0) – longer than humerus
(1) – shorter than humerus

State 0 is present in all tyrannosauroids, so it is not relevant for an in-group phylogenetic analysis.

Character 362: Radius and ulna separation:
(0) – radius and ulna clearly separate elements
(1) – distinct adherence or syndesmosis distally

State 0 is present in all tyrannosauroids, so it is not relevant for an in-group phylogenetic analysis.

Character 363: Scapula general form:
(0) – short and broad (ratio length/minimal height of shaft <9)
(1) – slender and elongate (ratio >10)

This character is extremely similar to character 234 in our dataset, and we prefer to retain our original character.

Character 364: Scapula, dorsal expansion of proximal blade measured from the dorsal surface at the scapulocoracoid suture to the most ventrally displaced dorsal surface of the scapular blade:
(0) – not more than twice the minimum scapular blade width
(1) – twice to 2.5 times the minimum scapular blade width
(2) – expanded more than three times the minimum scapular width
(3) – expanded more than four times the minimum scapular width

We are not able to verify the scores of Loewen et al. (2013) based on the comparative sample of tyrannosauroids available to us. We find that all tyrannosauroids we have been able to measure have a ratio of ~1.7-2.1, and therefore have very little variability among them. For the time being we exclude this character but may reintroduce it at a later stage as more data become available.

Character 365: Scapula, angle between dorsal arcromion process and dorsal surface of scapular blade:
(0) – sweeping curve
(1) – distinct angle greater than 100 degrees
(2) – distinct angle less than 95 degrees

This is a modified version of character 232 in our dataset, which simply differentiates between angles greater than or approximately equal to 90 degrees. Loewen et al. (2013) have changed the numerical cutoffs to 95 and 100 degrees, and added a new state that is not numerically quantified

(‘sweeping curve’). We cannot clearly distinguish the ‘sweeping curve’ condition from the ‘greater than 100 degrees’ condition: Loewen et al. (2013) score *Dilong* and *Guanlong* for the ‘sweeping curve’ and *Eotyrannus* for ‘greater than 100 degrees’, but we do not see any discrete, easy-to-define difference between them. Therefore, we prefer to retain a two-state character, and retain our original numerical cutoff of 90 degrees. Note that a retention of a 90 degree cutoff unites *Bistahieversor* and tyrannosaurids together as sharing a derived condition (state 1 in our dataset), whereas the numerical cutoffs of Loewen et al. (2013) instead unite *Bistahieversor* and tyrannosaurines (state 2) relative to albertosaurines and more basal tyrannosauroids (states 0 and 1). We retain our original 90 degree cutoff because our measurements indicate that the major difference among tyrannosauroids is between taxa that have a large obtuse angle (basal tyrannosauroids) and ones that have a nearly 90 degree angle (*Bistahieversor* and tyrannosaurids), whereas the differences encoded in the Loewen et al. (2013) character are much more subtle, so subtle that we have not been able to verify them with our own measurements.

Character 366: Scapula, distal end expansion relative to proximal portion of scapula:
(0) – markedly expanded
(1) – not expanded

State 0 is present in all tyrannosauroids, so it is not relevant for an in-group phylogenetic analysis.

Character 368: Scapula, acromion margin in rostrodorsal view:
(0) – continuous with blade in anterior aspect
(1) – anterior edge laterally everted relative to plane of scapular blade

State 0 is present in all tyrannosauroids, so it is not relevant for an in-group phylogenetic analysis.

Character 369: Scapula, acromion margin length:
(0) – long, either tapering or horizontal triangle
(1) – truncated with deep base but short reach beyond scapular blade and squared-off profile

State 0 is present in all tyrannosauroids, so it is not relevant for an in-group phylogenetic analysis.

Character 370: Scapula and coracoid form in posterior and anterior views:
(0) – continuous arc
(1) – coracoid inflected medially, scapulocoracoid ‘L’ shaped in lateral view

State 0 is present in all tyrannosauroids, so it is not relevant for an in-group phylogenetic analysis.

Character 371: Coracoid in lateral view:
(0) – subcircular, with shallow ventral process
(1) – subquadrangular with extensive ventral process
(2) – shallow ventral blade with elongate ventral process

State 0 is present in all tyrannosauroids, so it is not relevant for an in-group phylogenetic analysis.

<u>Character 373</u>: Posterolateral surface of coracoid ventral to glenoid fossa:
(0) – unexpanded
(1) – posterolateral edge of coracoids expanded to form triangular subglenoid fossa bounded laterally by enlarged coracoid tuber

State 0 is present in all tyrannosauroids, so it is not relevant for an in-group phylogenetic analysis.

<u>Character 374</u>: Ventral part of coracoid anterior to the glenoid facet:
(0) – approximately level with the rim of the facet
(1) – with tapering posteroventral process

State 1 is present in all tyrannosauroids, so it is not relevant for an in-group phylogenetic analysis.

<u>Character 375</u>: Glenoid fossa orientation:
(0) – faces posteriorly or posterolaterally
(1) – faces laterally

State 0 is present in all tyrannosauroids, so it is not relevant for an in-group phylogenetic analysis.

<u>Character 376</u>: Ossified sternal plates in adults:
(0) – ossified sternal plates absent
(1) – plates ossified, but separate in adults

State 0 is present in all tyrannosauroids, so it is not relevant for an in-group phylogenetic analysis.

<u>Character 377</u>: Humerus, form of shaft:
(0) – straight
(1) – sigmoid

This character was originally used by Brusatte et al. (2009) but was excluded when we critically revised characters for the Brusatte et al. (2010) phylogenetic study, as we found it difficult in practice to accurately define ‘straight’ and ‘sigmoid’ in a clear, discrete, repeatable manner. We also think that the ‘straight’ vs. ‘sigmoid’ distinction is essentially what is being encoded in our character 242, which is also used by Loewen et al. (2013) as their character 380.

<u>Character 387</u>: Manus, number of metacarpals present:
(0) – 5
(1) – 4

(2) – 3

State 3 is present in all tyrannosauroids, so it is not relevant for an in-group phylogenetic analysis.

Character 391: Metacarpal II, mediolateral width at midpoint:
(0) – equal to or narrower than metacarpal I
(1) – more robust than metacarpal I

This is included in our dataset as character 253.

Character 392: Metacarpal III, length:
(0) – subequal in length to MC II
(1) – much shorter than MC II

State 1 is present in all tyrannosauroids, so it is not relevant for an in-group phylogenetic analysis.

Character 393: Manual phalanx II-1, shaft diameter:
(0) – less than shaft diameter of radius
(1) – greater than shaft diameter of radius

State 0 is present in all tyrannosauroids, so it is not relevant for an in-group phylogenetic analysis.

Character 396: Manual unguals, degree of recurvature:
(0) – extensive, flexor margin deeply concave
(1) – thin and reduced, flexor margin shallowly concave
(2) – robust and reduced, flexor margin shallowly concave

This is a modified version of character 256 in our dataset, to which Loewen et al. (2013) added an additional state (state 1) that is relevant to other theropods, but not tyrannosauroids.

Character 397: Manual unguals, proximodorsal lip on some manual unguals, a transverse ridge immediately dorsal to the articulating surface:
(0) – absent
(1) – present

State 0 is present in all tyrannosauroids, so it is not relevant for an in-group phylogenetic analysis.

Character 398: Ilium, overall shape of blade:
(0) – sub-quadrangular or trapezoidal in lateral view
(1) – semi-oval outline in lateral view

This is equivalent to character 268 in our dataset (see our discussion of that character and how it relates to a similar character in Brusatte and Benson [2013] above).

Character 401: Ilium, blade height vs. length:
(0) – low and long
(1) – tall and short

This character is very similar to character 398 in the Loewen et al. (2013) dataset, which is included in our dataset as character 268. Characters 398 and 401 are not scored identically by Loewen et al. (2013), but they are clearly referring to the overall shape of the ilium. Furthermore, for the present character, 'long and low' and 'tall and short' are not quantified, so they are difficult to score consistently.

Character 402: Ilium, iliac blade orientation:
(0) – roughly vertical
(1) – dorsomedially inclined, dorsal edges of ilia approach or meet each other above sacrum

This character is equivalent to, and scored identically, to character 403 in Loewen et al. (2013), which is character 260 in our dataset. It is the dorsomedial inclination of the ilia (character 402) that cause them to lie against the neural spines (character 403).

Character 404: Ilium, postacetabular blade orientation in dorsal view:
(0) – subparallel
(1) – diverge posteriorly

Loewen et al. (2013) score all score-able tyrannosauroids for state 0, except for *Alioramus altai*, which is scored for state 1. This character may be an autapomorphy for *Alioramus altai*, and if so, is phylogenetically uninformative in an in-group phylogeny. If additional tyrannosauroids are discovered with state 1, and our comparative studies indicate that this is a clearly defined phylogenetic character, then we will reintroduce it into future versions of our dataset.

Character 405: Ilium, anterodorsal edge of ilium notch:
(0) – absent
(1) – notch at rostrodorsal corner

State 1 is present in all tyrannosauroids, so it is not relevant for an in-group phylogenetic analysis.

Character 407: Ilium, preacetabular part of ilium length relative to postacetabular ilium:
(0) – preacetabular ilium shorter
(1) – roughly as long as postacetabular part of ilium
(2) – longer than postacetabular ilium

We find that this character is encapsulating subtle differences that are difficult to score consistently in tyrannosauroids. A similar character is used in higher-level theropod phylogenetic analyses, including the TWiG coelurosaur dataset (e.g., Brusatte et al. 2014: character 152).

Among coelurosaurs as a whole, the major distinction is between: 1) a postacetabular process and preacetabular process that are roughly equivalent in length (which is seen in all tyrannosauroids); 2) a preacetabular process that is markedly longer than the postacetabular process, such that it is roughly 2/3 of total ilium length; and 3) a postacetabular process that is much longer than the preacetabular process. All tyrannosauroids fall into the first category and we do not wish to subdivide this category finely into states that refer to pre- and postacetabular processes that are only marginally longer than each other. Therefore, we exclude this character.

Character 408: Ilium, notch between pubic peduncle and pre-acetabular process:
(0) – wide, with semicircular or tear-drop outline
(1) – narrow and slit-like

This character was added to our dataset by Brusatte and Benson (2013) and is included as character 312 here.

Character 410: Ilium, dorsal extent of supraacetabular ridge:
(0) – short, does not extend into the m. iliofibularis scar
(1) – tall, extend onto the m. iliofibularis scar

We think that this character is extremely similar to character 308 in our analysis, and exclude so as to not retain redundant characters. Additionally, as scored by Loewen et al. (2013), state 0 is seen in all score-able tyrannosauroids except for *Stokesosaurus*, which has state 1. State 1 is an autapomorphy of *Stokesosaurus* among tyrannosauaroids, rendering this character uninformative for an in-group phylogenetic analysis.

Character 412: Ilium, supraacetabular crest on ilium as a separate process from antitrochanter:
(0) – present, forms "hood" over femoral head
(1) – reduced, not forming hood
(2) – absent

This character is essentially identical to character 414 in the Loewen et al. (2013) dataset, which is included in our dataset as character 262. Both characters 412 and 414 are scored identically by Loewen et al. (2013), supporting our interpretation of them as equivalent.

Character 415: Ilium, pubic peduncle articular facet:
(0) – flat or concave
(1) – convex
(2) – flexed with two facets set at obtuse angle to each other

Loewen et al. (2013) score all score-able tyrannosauroids for state 1, except for *Juratyrant*, which is scored for state 0. Therefore, as scored, this character is an autapomorphy for *Juratyrant* and is uninformative for an in-group phylogenetic analysis. Furthermore, we do not agree that there is a clear, distinct difference between the morphology of *Juratyrant* and other tyrannosauroids.

Character 417: Ilium, pubic peduncle width in lateral view:

(0) – uniform in width (i.e. unexpanded)
(1) – broadly flared toward pubic articular surface

State 1 is present in all tyrannosauroids, so it is not relevant for an in-group phylogenetic analysis.

Character 418: Ilium, pubic and ischial peduncles relative dorsoventral length:
(0) – comparable dorsoventral length
(1) – pubic peduncle expanded and twice or more as long as ischidiac peduncle

State 1 is present in all tyrannosauroids, so it is not relevant for an in-group phylogenetic analysis.

Character 420: Ilium, cuppedicus fossa (‘preacetabular fossa’ of Hutchinson, 2001a):
(0) – absent
(1) – present, formed as antiliac shelf anterior to acetabulum, extends posteriorly to above anterior end of acetabulum
(2) – present, posterior end of fossa on anterior end of pubic peduncle, anterior to acetabulum

State 1 is present in all tyrannosauroids, so it is not relevant for an in-group phylogenetic analysis.

Character 421: Ilium, brevis fossa lateral exposure:
(0) – entire length of fossa visible in lateral view, including caudal portion
(1) – only the anterior portion of the fossa is visible, the caudal portion of the fossa is hidden by posterior blade of the ilium
(2) – nearly completely hidden by posterior blade of the ilium

A very similar character was added to our dataset by Brusatte and Benson (2013) and is included here as character 310.

Character 424: Pubis, pubic boot, posterior expansion:
(0) – present
(1) – little or no posterior process

State 0 is present in all tyrannosauroids, so it is not relevant for an in-group phylogenetic analysis.

Character 425: Pubis, pubic boot, anterior expansion:
(0) – absent
(1) – present, short
(2) – present, long

This character is similar to character 426 in Loewen et al. (2013), which is included in our dataset as character 273. We prefer the use of this character as it is quantitative, whereas the

current Loewen et al. (2013) character is qualitative and terms such as ‘short’ and ‘long’ are not defined.

Character 427: Pubis, entire pubic boot, anteroposterior length relative to total long axis length of pubis:
(0) – less than 25%
(1) – 30-35%
(2) – 40-55%
(3) – 60-70%
(4) – greater than 75%

Loewen et al. (2013) modified character 272 in our dataset, transforming it from a binary character (pubic boot less/greater than 60% length of pubis) into a multistate with numerous states distinguishing an ordered sequence of subtle differences in the proportional size of the boot. We prefer our simple binary character which encapsulates the major disparity among tyrannosauroids—a small vs. huge pubic boot—rather than a complex ordered multistate with different degrees of ‘smallness’ and ‘largeness’.

Character 429: Pubis, obturator foramen:
(0) – present
(1) – open notch present between pubic shaft and ishciadic peduncle
(2) – neither notch or foramen present

State 1 is present in all tyrannosauroids, so it is not relevant for an in-group phylogenetic analysis.

Character 430: Pubis, pubic shaft shape:
(0) – rounded
(1) –mediolaterally flattened

State 0 is present in all tyrannosauroids, so it is not relevant for an in-group phylogenetic analysis.

Character 431: Pubis, contact between pubes:
(0) – both pubes meet extensively
(1) – contact disrupted by a slit-like opening

State 1 is present in all tyrannosauroids, so it is not relevant for an in-group phylogenetic analysis.

Character 434: Ischium, length compared to the length of the pubis:
(0) – more than two-thirds
(1) – two-thirds or less of pubis length

State 0 is present in all tyrannosauroids, so it is not relevant for an in-group phylogenetic analysis.

Character 437: Ischium, ilio-ischiadic contact:
(0) – ischium with slightly concave or flat proximal articular surface that contacts ilium
(1) – with deep socket for reception of peg-like ischial peduncle of ilium

State 1 is present in all tyrannosauroids, so it is not relevant for an in-group phylogenetic analysis.

Character 438: Ischium, transverse cross-section of acetabular rim:
(0) – convex or beveled
(1) – broad shelf with longitudinal sulcus or depression

We do not understand this character and therefore exclude it. Loewen et al. (2013) score all score-able tyrannosauroids for state 1, except for *Guanlong*, which is state 0. However, we do not see a distinct, clear, easy-to-define difference between *Guanlong* and, for example, *Juratyrant*.

Character 440: Ischium, obturator foramen and flange:
(0) – present
(1) – reduced to notch, flange confluent with pubic peduncle
(2) – notch or foramen absent, U shaped gap between pubic peduncle and obturator process

Loewen et al. (2013) score *Guanlong* for state 0, *Juratyrant* for state 1, and all other score-able tyrannosauroids for state 2. *Guanlong* is genuinely unique among tyrannosauroids in possessing an enclosed obturator foramen within a large obturator flange. This is shared with many distant outgroups and is likely a reversal to a primitive theropod feature (as it is not seen in close outgroups), making it an autapomorphy of *Guanlong* among tyrannosauroids based on current knowledge. However, we do not recognize a distinct, clear, easy-to-define difference between *Juratyrant* and other non-*Guanlong* tyrannosauroids. Instead, we see the major distinction among tyrannosauroids as between the *Guanlong* condition and that of everything else. As the *Guanlong* condition is currently optimized as an autapomorphy we exclude this character here, but will reintroduce it in future versions of our dataset if additional tyrannosauroids are discovered with an enclosed obturator foramen.

Character 441: Ischium, position of apex of obturator flange, distance between apex and proximal end of ischium:
(0) – proximal flange, position at less than 20% of ischum length
(1) – flange positioned at approximately 25-30% of ischium length
(2) – flange position at approximately 35% of ischium length
(3) – distal flange, position further distal than 50% of ischium length

Loewen et al. (2013) modified our binary character into a multistate with several states distinguishing an ordered sequence of differences in the position of the apex of the obturator flange. We prefer our simple binary character rather than a complex ordered multistate that is seemingly distinguishing very subtle differences in the position of the flange, which can be difficult to score accurately if preservation is poor.

Character 442: Ischium, broad medially deflected apron from obturator flange to distal end of ischium:
(0) – absent
(1) – present, covers 1/3 to 1/2 the ventral surface between obturator flange to distal end of ischium
(2) – present, covers more than 1/2 the ventral surface between obturator flange to distal end of ischium

We do not understand this character. For example, Loewen et al. (2013) score *Guanlong* and *Juratyrant* for state 0, the lack of a 'broad medially deflected apron' on the ischium. However, both taxa clearly have medial aprons which span most of the proximodistal length of the ischia. Therefore, we exclude this character because we are not certain what the different states refer to.

Character 443: Ischium, scar on posterior edge of the proximal end of the ischium:
(0) – absent
(1) – present

This is character 278 in our dataset.

Character 444: Femur, femoral head orientation:
(0) – directed ventrally
(1) – directed horizontally
(2) – directed dorsally

This character has been used often in higher-level theropod phylogenetic analyses, including the TWiG coelurosaur dataset. Brusatte et al. (2014, character 711) revised this character, scoring all tyrannosauroids (including *Guanlong* and other basal taxa) as having a proximally/dorsally inclined femoral head, which is equivalent to state 2 in the Loewen et al. (2013) character. Two tyrannosaurids, Tyrannosaurus and Tarbosaurus, have a derived condition in which the proximal margin of the head is concave and oriented at an obtuse angle to the long axis of the shaft, due to a head that is proximally inclined and a greater trochanter that is elevated substantially relative to the central portion of the proximal surface of the femur. This latter feature is encapsulated in a separate character, character 285, in our dataset. We have rewritten character 285 so that all of these features are clearly explained.

Character 445: Femur, femoral head orientation relative to the distal femoral condyles:
(0) – directed anteromedially
(1) – directed strictly medially

State 1 is present in all tyrannosauroids, so it is not relevant for an in-group phylogenetic analysis.

Character 446: Femur, fossa on the posterior surface of the femoral head, just lateral to the articular surface:
(0) – absent or shallow

(1) – narrow channel
(2) – deep, wide fossa

This character is very similar to character 286 in our dataset, which refers to the trochanteric fossa. The fossa being referred to in the Loewen et al. (2013) character is described as ‘just lateral to the articular surface’, indicating that it is more likely to be the ligament sulcus and not the trochanteric fossa. However, we see no distinct differences in the size and depth of the ligament sulcus across tyrannosauroids. For example, *Guanlong* (scored for state 1 by Loewen et al. [2013]) has a deep sulcus just like *Alioramus altai* and *Tyrannosaurus* (scored for state 2). Furthermore, *Xiongguanlong* is scored for state 0 but clearly has a ligament sulcus. As a result, we are confused about what this character represents. It is either referring to the ligament sulcus, in which case we recognize no discrete differences among tyrannosauroids, or to the trochanteric fossa in which case this is already encapsulated by character 286 in our dataset.

Character 447: Femur, anterior trochanter, presence and separation from shaft:
(0) – absent
(1) – separated from greater trochanter by deep cleft
(2) – trochanters separated by small groove

State 1 is present in all tyrannosauroids, so it is not relevant for an in-group phylogenetic analysis.

Character 448: Femur form of anterior trochanter:
(0) – alariform
(1) – cylindrical in cross section

State 0 is present in all tyrannosauroids, so it is not relevant for an in-group phylogenetic analysis.

Character 450: Femur, vertical ridge on lateral face of anterior trochanter:
(0) – present
(1) – absent

State 0 is present in all tyrannosauroids, so it is not relevant for an in-group phylogenetic analysis.

Character 451: Femur, fourth trochanter, position, measurement from proximal margin of head to midpoint of fourth trochanter relative to total length of the femur:
(0) – proximally located, distance 25% or less femoral length
(1) – distally located, distance 30% of greater femoral length

Loewen et al. (2013) slightly modified our character 288 by making small changes to the quantitative cutoffs in our character states, but we prefer our original language.

Character 452: Femur, circular scar (M. adductor femoralis 1) on posterior surface of shaft distal to fourth trochanter, mediolateral position:

(0) – scar absent
(1) – positioned closer to the medial edge of shaft
(2) – positioned closer to the shaft midline

Loewen et al. (2013) modified character 283 in our dataset by adding an additional state. We have modified character 283 here to take into account this information.

Character 453: Femur, anteromedial crest on anterior surface proximal to medial distal condyle:
(0) – without longitudinal crest
(1) – crest present extending proximally from medial condyle on anterior surface of shaft

Loewen et al. (2013) score all score-able tyrannosauroids for state 1, except for three taxa scored for state 0: *Bagaraatan*, *Raptorex*, and *Alectrosaurus*. As discussed above, we do not include *Bagaraatan* and *Alectrosaurus* in our dataset. This leaves *Raptorex* as the only tyrannosauroid potentially possessing state 0. However, we do not recognize any clear, easy-to-define distinction between *Raptorex* and other tyrannosauroids, so we exclude this character.

Character 456: Femur, depression between distal condyles in anterior view:
(0) – relatively flat between condyles
(1) – shallow depression between condyles
(2) – deep depression confluent with extensor groove on anterior surface

This character is referring to the morphology of the anterior surface of the distal end of the femur, particularly whether it is flat, shallowly depressed, or deeply concave. Our character 290 refers to something very similar: the presence/absence of an extensor groove and whether the extensor groove is shallowly depressed or deeply concave. Loewen et al. (2013) do include our character 290 as character 454 in their dataset, and they indicate that character 456 is not referring to the extensor groove, as they note that the depression they are discussing is 'confluent with the extensor groove'. However, we do not understand this character description. We do not recognize any discrete depression on the anterior surface of the distal end of the femur of tyrannosauroids; rather, this depression is the extensor groove. Therefore, we consider this character to be essentially equivalent to character 290 in our dataset, and charater 454 in Loewen et al. (2013), and exclude it.

Character 457: Femur, deep channel between condyles for M. gastrocnemius and flexor digitorum longus group in distal view:
(0) – no discrete channel present between condyles
(1) – wide channel, wider than deep
(2) – channel deeper than wide

Based on our comparative study of tyrannosauroids, we do not find a clear, distinct, easy-to-define difference in the shape of this channel among taxa. For example, Loewen et al. (2013) score *Alioramus* for a 'wider than deep' channel and *Tyrannosaurus* for a 'deeper than wide' channel, but we do not recognize a clear difference between them. We exclude this character for the time being, but may reintroduce it at later time as more data become available.

Character 458: Femur, crista tibiofibularis on lateral condyle in distal view:
(0) – no distinct lateral separation in lateral condyle
(1) – distinct lateral constriction into crista tibiofibularis larger than half the mediolateral width of the lateral condyle
(2) – crista tibiofibularis restricted to medial half lateral condyle

This is cited as a modified version of character 289 in our dataset, but our character refers to something different: the shape of the anterior margin of the lateral condyle of the tibia when it is seen in distal view. The Loewen et al. (2013) character, on the other hand, has to do with the size of the crista tibiofibularis. We agree that there is variation in the size of this structure among tyrannosauroids, and we add this character to our dataset as character 356.

Character 461: Tibia, lateral ridge on the lateral side of the tibia for connection with fibula:
(0) – extending from the proximal articular surface distally
(1) – clearly separated from proximal articular surface

State 1 is present in all tyrannosauroids, so it is not relevant for an in-group phylogenetic analysis.

Character 462: Tibia, lateral condyle on proximal end of tibia:
(0) – confluent with cnemial crest anteriorly in proximal view, or only slightly offset
(1) – strongly offset from cnemial crest

State 1 is present in all tyrannosauroids, so it is not relevant for an in-group phylogenetic analysis.

Character 465: Fibula, excavated medial fossa on the proximal end of the fibula:
(0) – absent
(1) – present, shallow
(2) – present, deep

A similar character is used often in higher-level theropod phylogenetic analyses, including the TWiG coelurosaur analysis. In the latest version of this analysis, Brusatte et al. (2014: character 190) uses a binary character that distinguishes between the two major conditions in coelurosaurs: the absence or presence of a deep oval-shaped fossa with well-defined margins. This fossa is scored as present in all score-able tyrannosauroids. Loewen et al. (2013), in effect, divide the 'present' condition into separate states for 'shallow' and 'deep' fossae. We have two issues with this. First, 'shallow' and 'deep' are not quantified, so they are difficult to score consistently. For example, Loewen et al. (2013) score *Appalachiosaurus* for the 'shallow' condition and *Tyrannosaurus* for the 'deep' condition, but we do not recognize any distinctive difference between them. Second, we do not wish to subdivide this character too finely, which would make it difficult to score across a wide range of theropods, and prefer the simple presence/absence character that encapsulates the major variation among taxa. Therefore, as we consider all tyrannosauroids to have the same condition (a deep ovoid fossa), we exclude this character.

Character 466: Fibula, position of medial fossa:

(0) – not extensive, proximal border starts about 1/3 the distance between the proximal surface and the iliofibularis tubercle
(1) – fills most of the proximal end of the fibula

Loewen et al. (2013) scores all score-able tyrannosauroids for state 1, except for *Guanlong*, which is scored for state 0. We cannot confirm the score in Guanlong because the holotype specimen we have observed has a tibia and fibula that are appressed together, so the medial surface of the fibula cannot be seen clearly. Therefore, although this character may unite all non-*Guanlong* tyrannosauroids, we exclude it because we cannot score it confidently at this time. We may reintroduce it into future versions of our analysis as more data become available.

<u>Character 468</u>: Fibula, elongate groove on medial surface distal to iliofibularis turbercle:
(0) – absent
(1) – present, distal fibula medially concave

Loewen et al. (2013) score the groove as present (state 1) in all score-able tyrannosauroids except for five taxa. Two, *Bagaraatan* and *Alectrosaurus*, are not in our dataset. *Guanlong* cannot be scored confidently for the same reasons as discussed for character 467 above. *Dryptosaurus* is scored incorrectly: the specimen is poorly preserved but there is a shallow groove distally on the medial surface of the fibula shaft. For the final taxon, *Raptorex*, we cannot confirm the score. Therefore, all tyrannosauroids that we can confidently score have state 1. We exclude this character for the time being, but may reintroduce it into future versions of our analysis as more data become available.

<u>Character 470</u>: Astragalus, height of ascending process of astragalus (height vs. height of body):
(0) – lower than astragalar body
(1) – higher than the astragalar body, typically covering only lateral half of anterior surface of distal tibia
(2) – more than twice the height of astragalar body

State 2 is present in all tyrannosauroids, so it is not relevant for an in-group phylogenetic analysis.

<u>Character 471</u>: Astragalus, ascending process height (height vs. width):
(0) – less than half the width of the bone
(1) – half the width of the bone to equal to the width of the bone
(2) – greater than the width of the astragalas and calcaneum

This character was originally used by Brusatte et al. (2009) but was excluded when we critically revised characters for the Brusatte et al. (2010) phylogenetic study, as we were concerned that this character was at least partially redundant with character 297 in our dataset, which concerns the proportions of the base of the ascending process (this character is used as character 472 in the Loewen et al. [2013] dataset). The two characters are scored nearly identically by Loewen et al. (2013), lending support to the interpretation that they are essentially redundant. Therefore, we exclude.

Character 473: Astragaus, anteroposterior thickness of ascending process:
(0) – wedge-shaped/blocky
(1) – plate-like/laminar

State 1 is present in all tyrannosauroids, so it is not relevant for an in-group phylogenetic analysis.

Character 474: Astragalus, ascending process contact with fibula:
(0) – absent or limited
(1) – extensive

State 1 is present in all tyrannosauroids, so it is not relevant for an in-group phylogenetic analysis.

Character 475: Astragalus, fibular facet on body of astragalus:
(0) – large and facing partially proximally
(1) – reduced and confined to anterior half of lateral side of astragalus
(2) – strongly reduced, facing laterally or absent

State 2 is present in all tyrannosauroids, so it is not relevant for an in-group phylogenetic analysis.

Character 476: Astragalar condyles:
(0) – almost entirely below tibia and face distally
(1) – significantly expanded proximally on anterior side of tibia and face anterodistally

State 1 is present in all tyrannosauroids, so it is not relevant for an in-group phylogenetic analysis.

Character 477: Astragalus, horizontal groove across astragalar condyles anteriorly:
(0) – absent
(1) – present

State 0 is present in all tyrannosauroids, so it is not relevant for an in-group phylogenetic analysis.

Character 478: Astragalus, posterior process:
(0) – completely caps distal tibia
(1) – curves only slightly proximally, but not strongly covering the posterior side of the tibia
(2) – significant portion of distal surface exposed on articular facet so that astragalas and cancanium do not cover entire distal tibia

State 1 is present in all tyrannosauroids, so it is not relevant for an in-group phylogenetic analysis.

Character 479: Calcaneum, articulation with tibia:

(0) – without facet for tibia
(1) – with tibial facet on posteromedial corner or posterior surface

State 1 is present in all tyrannosauroids, so it is not relevant for an in-group phylogenetic analysis.

Character 480: Distal tarsal fusion:
(0) – separate, not fused to metatarsals
(1) – fused, forming metatarsal cap with intercondylar prominence that fuses to metatarsal early in postnatal ontogeny

State 0 is present in all tyrannosauroids, so it is not relevant for an in-group phylogenetic analysis.

Character 481: Metatarsals, appression of major metatarsal shafts:
(0) – not appressed against each other beyond proximal half of shaft
(1) – appressed throughout most or all of metatarsus, adjacent surfaces of shafts flattened for contact between metatarsals metatarsals closely appressed and distance between II-III and III-IV is approximately equal
(2) –distance between mt III- mt IV greater than that between mt II- mt III

This is a modified version of character 302 in our dataset. Loewen et al. (2013) add a new state referring to taxa in which the metatarsals are not closely appressed to each other (state 0), which is not relevant to tyrannosauroids. In our dataset we score *Appalachiosaurus* and more derived tyrannosauroids for state 1 (equivalent to state 2 in Loewen et al [2013]) and Guanlong and *Yutyrannus* for state 0 (equivalent to state 1 in Loewen et al [2013]). Loewen et al. (2013) changes several of these scores, and considers *Appalachiosaurus* and *Alioramus remotus* to share the primitive condition with *Guanlong* and *Yutyrannus* (state 1 in their dataset). They are also more liberal in scoring taxa in the affirmative: they score *Dryptosaurus* and *Eotyrannus* for the primitive condition (state 1 in their dataset) and *Bistahieversor* for the derived condition (state 2 in their dataset), whereas we score them conservatively as '?' because we do not feel that enough material is preserved for a confident score. We retain our original character usage and scores based on our comparative study of tyrannosauroids.

Character 482: Metatarsals II and IV, relative length when in articulation:
(0) – metatarsals II and IV subequal in length
(1) – mt II longer than mt IV
(2) – mt II shorter than mt IV

This character is incredibly variable across tyrannosauroids, without a clear phylogenetic signal (i.e., agreement with other characters). It also depends on having a complete and articulated metatarsus, without much distortion, to score it accurately. Therefore, we exclude this character for the time being, but may reintroduce it in the future when more data are available.

Character 484: Metatarsal II, tear drop shaped articular surface for metatarsal III:
(0) – absent

(1) – present

We interpret this character as essentially equivalent to the preceding character in the Loewen et al. (2013) dataset, which is character 303 in our dataset, scoring for the absence/presence of a heavily rugose articular surface on metatarsal II for metatarsal III. Based on our comparative study of tyrannosauroids, the heavily rugose fossa is always large and tear-drop shaped, and we think that these two features are most likely linked, as they both have to do with the morphology of a highly modified articular surface related to the arctometatarsalian condition of derived tyrannosauroids. These two characters are also scored nearly identically by Loewen et al. (2013).

<u>Character 485</u>: Metatarsal II, relative size of midshaft compared to metatarsal IV:
(0) – subequal
(1) – metatarsal II more robust than metatarsal IV

Loewen et al. (2013) finds state 1 to diagnose a group of derived tyrannosauroids more derived than *Appalachiosaurus*, which has state 0. However, based on our comparative study of tyrannosauroids, we find the differences between basal and derived tyrannosauroids to be subtle. In all tyrannosauroids metatarsals II and IV are very nearly the same width. There may be tiny differences between them, but these are usually on the order of just a few percent, and they pale in comparison to the great differences in mt II and IV widths in other theropods. Therefore, we exclude this character.

<u>Character 487</u>: Metatarsal III, outline of proximal articular surface:
(0) – rectangular
(1) – hourglass-shaped
(2) – wedge-shaped with no exposure on plantar surface

State 1 is present in all tyrannosauroids, so it is not relevant for an in-group phylogenetic analysis.

<u>Character 489</u>: Metatarsal III, proximal shaft exposure:
(0) – prominently exposed between MT II and MT IV along entire metapodium
(1) – MT III proximal shaft constricted and much narrower than either II or IV, but still exposed along most of metapodium, subarctometatarsal
(2) – very pinched, poorly exposed exposed along proximal section of metapodium, arctometatarsal condition, mt III wedge-shaped with proximal end covered anteriorly by contact between metatarsals II and IV

This character relates to the arctometatarsalian condition of the pes, a feature that we encapsulate in character 299 in our dataset. We are concerned about including multiple characters that all relate to the same basic feature, and for that reason we exclude this character.

<u>Character 491</u>: Metatarsal IV, proximal end, medial notch for articulation with metatarsal III:
(0) – absent
(1) – present, shallow notch
(2) – present, deep notch

We have two issues with this character. First, it relates to the arctometatarsalian condition: the notch is present in arctometatarsalian taxa. For the same reasons reviewed for character 489, we exclude this character. Second, although it is possible that arctometatarsalian taxa may differ in the morphology of the notch (Loewen et al.'s states 1 and 2), we do not find any clear evidence for this. Loewen et al. (2013) score *Dryptosaurus* and *Appalachiosaurus* for state 1 and tyrannosaurids for state 2. We see no clear, discrete, easy-to-define distinction between these taxa, and in fact, Brusatte et al. (2011) described *Dryptosaurus* as having "the shape and size of the notch for metatarsal III (that is) nearly identical to that of derived tyrannosauroids such as *Albertosaurus*, *Appalachiosaurus*, *Gorgosaurus* (and others)." Therefore, as 'shallow' and 'deep' are not quantified, we exclude this character.

<u>Character 492</u>: Metatarsal IV, well-developed posteromedial flange on proximal end for articulation with metatarsal III:
(0) – absent
(1) – present

State 0 is present in all tyrannosauroids, so it is not relevant for an in-group phylogenetic analysis.

<u>Character 493</u>: Metatarsal IV, midshaft, articular surface for metatarsal III:
(0) – flat
(1) – concave

We consider this character to be scoring for the same morphology encapsulated in characters 483 and 484 in the Loewen et al. (2013) dataset, which concern the presence/absence and morphology of a large, rugose, tear-drop shaped articular surface on metatarsal II for metatarsal III. Those taxa with the fossa on metatarsal II also have a similar fossa on metatarsal IV, which articulates with the other side of metatarsal III. This is related to the arctometatarsalian condition in derived tyrannosauroids. For those taxa that can be scored affirmatively (i.e., not as '?') for both characters, those taxa with a concave articular surface on metatarsal IV (character 493) are also scored for the tear-drop shaped articular surface on metatarsal II (character 484). Therefore, we consider character 493 redundant with characters 483/484 (see our discussion of these characters above).

<u>Character 494</u>: Metatarsal IV, articular surface for metatarsal III tear drop shaped:
(0) – absent
(1) – present

Following the same rationale explained above for characters 483/484, we consider this character to be redundant with character 493.

<u>Character 496</u>: Metatarsal IV, surface between metatarsal III articulation and posterior oval rugosity:
(0) – flat
(1) – concave

(2) – slender ridge between metatarsal III articulation and posterior oval rugosity

We exclude this character because we do not fully understand the differences between the character states and cannot reproduce the character scores of Loewen et al. (2013) based on our own comparative study of tyrannosauroids. As a result, we do not find that we can score this character confidently. We may reintroduce it into our dataset at a later time if more data become available, or if the character is more fully explained and clearly illustrated in the literature.

Character 497: Metatarsal IV, depth of medial collateral ligament fossa:
(0) – shallow, not rimmed
(1) – deep and rimmed

We are not able to recognize any clear, discrete, easy-to-define differences among tyrannosauroids. Loewen et al. (2013) score most tyrannosauroids for state 1, but *Guanlong* and *Dryptosaurus* for state 0. We do not agree with these scores. The holotype specimen of *Guanlong* that we examined has an articulated metatarsus so we cannot assess the state of the medial ligament pit, but judging from the very deep lateral ligament pit on metatarsal IV the medial pit was most likely deep. *Dryptosaurus* has a deep medial ligament pit that appears somewhat shallower because of the very poorly preserved condition of the specimen. Therefore, all score-able tyrannosauroids have state 1, so this character is uninformative for an in-group analysis.

Character 499: Pedal unguals, relative size of unguals II and III:
(0) – subequal
(1) – II much larger forming sickle claw

State 0 is present in all tyrannosauroids, so it is not relevant for an in-group phylogenetic analysis.

Character 501: Pedal unguals, curvature of flexor surface:
(0) – curved
(1) – relatively straight

State 0 is present in all tyrannosauroids, so it is not relevant for an in-group phylogenetic analysis.

5. Extended Discussion on the Tyrannosaurid Body Plan

<u>*Xiongguanlong* + derived Tyrannosauroids – Establishment of the 'Modern' Tyrannosaurid-grade Bauplan</u>: We agree with Li et al. (2010) that the phylogenetic position of *Xiongguanlong* is intermediate between basal, *Proceratosaurus-Stokesosaurus* grade tyrannosauroids and more derived tyrannosauroids. However, we regard *Xiongguanlong* as the earliest and first known example of the true tyrannosaurid grade bauplan. To their characters we add, among others, the transverse orientation of the premaxillary tooth row, mesiodistally asymmetrical carinae of the maxillary teeth, the ventrally convex profile of the dentigerous region of the maxilla, ilia that extend to the dorsal midline, squared-off postacetabular process of the ilium, and the deep extensor groove on the ilium. *Xiongguanlong* represents the first definitive tyrannosaurid-grade tyrannosauroid, where modifications to the jaws, teeth, and hips are seen that suggest an enhanced bite force and cursorial locomotor apparatus.

The relatively great morphological differences of *Xiongguanlong* from more basal tyrannosauroids are consistent with the approximately 20-45 million year temporal gap that separates them. Owing to the imprecise geological age of *Xiongguanlong*, the tyrannosaurid grade bauplan arose sometime between the Kimmeridgian and the Albian, perhaps in Asia, although the highly incomplete fossil record makes this uncertain. Future discoveries in rocks older and younger than *Xiongguanlong* will better reveal the stepwise acquisition of the tyrannosaurid-grade characters.

If the relative success of the basal tyrannosauroid- and tyrannosaurid grade bauplans are to be measured by longevity and diversification, then the suite of features that tyrannosaurids inherited from their common ancestor with *Xiongguanlong* may have ensured their success. Despite its wide distribution and relatively complex phylogenetic history, the basal grade of tyrannosauroids was extinct by the end of the Aptian. If the known diversity of Late Cretaceous tyrannosaurids is any indication, then they experienced a sustained diversification to the end of the Mesozoic since (at least) the Albian.

<u>Along the Line to *Tyrannosaurus* – Trends in Derived Tyrannosauroid Evolution</u>

<u>*Cephalic ornamentation*</u>: The appearance of the lacrimal horn is seen in the common ancestor of *Appalachiosaurus*+ derived tyrannosauroids. In the common ancestor of *Bistahieversor*+derived tyrannosauroids the process is inflated in adults and it is greatly increased in height, making it a prominent feature on the skull. In the common ancestor of albertosaurines+derived tyrannosauroids, the lacrimal horn gains a discrete apex, which enhances its shape relative to the snout. Finally, in the common ancestor of *Tarbosaurus* and *Tyrannosaurus,* the process is so grossly inflated in adults that it is obliterated and effectively absent.

The postorbital horn is seen in the *Bistahieversor*+derived tyrannosauroid clade, where the process is positioned close to the orbit margin. In the common ancestor of albertosaurines+derived tyrannosauroids, the horn extends dorsally past the dorsal edge of the skull, which enhanced its appearance relative to the dorsal skull roof. Finally, in the common ancestor of teratophonines+derived tyrannosaurines, the process is positioned posterodorsal to the orbit margin.

<u>*Enhanced bite force*</u>: Several lines of evidence suggest that the bite force of tyrannosaurid-grade tyrannosauroids increased throughout their evolutionary history. In the common ancestor of

Xiongguanlong and derived tyrannosauroids, the carinae of the maxillary teeth are asymmetrical; this condition is in part produced by a prominent strut that extends along the mesiolabial corner of the tooth from its base, this strut would resist bending under high loads.

In the common ancestor of *Appalachiosaurus* and derived tyrannosauroids, the height of the mandibular ramus is increased, which indicates improved resistance to higher loads imposed by massive adductor musculature and larger prey. Also, a bony brace is seen at the symphysis, which would resist dislocation between the complementary rami during prey capture and feeding.

In the common ancestor of *Bistahieversor* and tyrannosaurids, the maxilla is increased in height, which indicates the necessity for a stable snout capable of resisting the forces of increased loads imposed by prey capture, feeding, and the animal's own enhanced adductor musculature.

In the common ancestor of Tyrannosauridae, a long interfenestral strut provides the snout with a strut capable of withstanding high vertical loadings through the middle of the snout. In addition to this, a brace-like ridge stabilizes the jugopostorbital contact that extends across the postorbital bar. The ridge prevented caudalward separation of the postorbital over the jugal in this transition zone between the snout and temporal chamber.

In the common ancestor of Tyrannosaurinae, the antorbital sinus is more completely enclosed by bone, where the medial edge of the palatal process of the maxilla is extended dorsally, concealing the dorsum of the process from view. This increased enclosure of bone would have reduced the strain of biting. A distinct brace-like process extends from the ventrolateral surface of the palatine to abut the medial surface of the maxilla. This structure implies the necessity to resist the mediolateral forces imposed on the proximal end of the snout. Intracranial buttressing is also seen in the braincase, where the antotic crest of the laterosphenoid is a large strut, which would have resisted mediolateral and vertical loads. Modifications are also seen at the front of the snout, where the premaxillary processes of the nasals are more extensively in apposition with each other, which would have resisted loads at the front of the snout.

In the common ancestor of teratophonines and derived tyrannosaurines, the lateral edge of the dorsotemporal fossa is split into a pair of ridges. This condition implies a change in the size, force, and vectors of the adductor musculature. A more stable contact is seen between the jugal and quadratojugal, where the jugal extends caudally more extensively over the quadratojugal. In turn, the contact of the quadratojugal is dorsoventrally expanded on the lateral surface of the jugal. Evidence for an increase in the size of the adductor musculature is seen in the long and tall sagittal crest of the frontal, which extends the attachment surface for the jaw adductors dorsally. Also, the surangular shelf of the lower jaw extends ventrolaterally, which would have lengthened and oriented the vectors of the laterals slips of the adductors more steeply. The ventral margin of the mandibular ramus also reflects an increase in bite force in that it is sharply angled between the caudal adductor region and the dentigerous region. Finally, derived tyrannosaurines have a reduction in the number of maxillary teeth, which may be an epiphenomenon of tooth enlargement that is necessary to resist the loads of high bite forces.

In the common ancestor of *Lythronax* and derived tyrannosaurines, the verticalization of the adductor surface of the surangular is taken to and extreme, where it faces more laterally than dorsally. This would enhance the effect upon the lateral slips of the adductor musculature imposed by the verticalized surangular shelf.

In the common ancestor of *Daspletosaurus* and derived tyrannosaurines, intracranial buttressing is seen in the form of a strut along the leading edge of the external antorbital fenestra that conceals the promaxillary fenestra from lateral view. The nasomaxillary suture is highly modified into a peg-in-socket and serrate joint surface and suture that was almost certainly required to resist loads from bites that extended from the front half of the tooth row to the top of the snout. The constriction of the nasals between the medially expanded lacrimals may also indicate an arrangement to reduce loads at the zone between the snout and the adductor chamber. Intracranial buttressing is also seen on the lateral surface of the jugal, where a pair of dorsally extending and converging ridges produces a deep fossa in the base of that bone's dorsally extending postorbital process.

Finally, in the common ancestor of *Tarbosaurus* and *Tyrannosaurus*, the maxillary and dentary teeth are greatly increased in height and width, where the width of the crowns is nearly equal to its length. This caniniform shape is consistent with the high bite forces implied by the wide adductor chamber and deep mandibular ramus seen in this clade.

6. References

Averianov, A.O., S.A. Krasnolutskii, and S.V. Ivantsov. 2010. A new basal coelurosaur (Dinosauria: Theropoda) from the Middle Jurassic of Siberia. Proceedings of the Zoological Institute 314: 42–57.

Benson, R.B.J. 2008. New information of *Stokesosaurus*, a tyrannosauroid (Dinosauria: Theropoda) from North America and the United Kingdom. Journal of Vertebrate Paleontology 28: 732–750.

Benson, R.B.J., R.J. Butler, M.T. Carrano, and P.M. O'Connor. 2012. Air-filled postcranial bones in theropod dinosaurs: physiological implications and the 'reptile-bird transition. Biological Reviews 87, 168-193.

Bever, G.S., S.L. Brusatte, T.D. Carr, X. Xu, A.M. Balanoff, and M.A. Norell. 2013. The braincase anatomy of the Late Cretaceous dinosaur *Alioramus* (Theropoda: Tyrannosauroidea). Bulletin of the American Museum of Natural History 376: 1-72.

Britt, B.B. 1993. Pneumatic Postcranial Bones in Dinosaurs and Other Archosaurs. Unpublished PhD Thesis, University of Calgary.

Brochu, C.A. 2003. Osteology of *Tyrannosaurus rex*: insights from a nearly complete skeleton and high-resolution computed tomographic analysis of the skull. Society of Vertebrate Paleontology Memoir 7: 1-138.

Brusatte, S.L. 2013. The Phylogeny of Basal Coelurosaurian Theropods (Archosauria: Dinosauria) and Patterns of Morphological Evolution during the Dinosaur-Bird Transition. Unpublished PhD Dissertation, Columbia University, New York, 958 pp.

Brusatte, S.L., and R.B.J. Benson. 2013. The systematics of Late Jurassic tyrannosauroids (Dinosauria: Theropoda) from Europe and North America. Acta Palaeontologica Polonica 58: 47-54.

Brusatte, S.L., R.B.J. Benson, P.J. Currie, and X.-J. Zhao. 2010b. The skull of *Monolophosaurus jiangi* (Dinosauria: Theropoda) and its implications for early theropod phylogeny and evolution. Zoological Journal of the Linnean Society 158: 573-607.

Brusatte, S.L., R.B.J. Benson, and M.A. Norell. 2011. The anatomy of *Dryptosaurus aquilunguis* (Dinosauria: Theropoda) and a review of its tyrannosauroid affinities. American Museum Novitates 3717: 1-53.

Brusatte, S.L., T.D. Carr, G.M. Erickson, G.S. Bever, and M.A. Norell. 2009. A long-snouted, multi-horned tyrannosaurid from the Late Cretaceous of Mongolia. Proceedings of the National Academy of Sciences (USA) 106: 17261-17266.

Brusatte, S.L., T.D. Carr, and M.A. Norell. 2012. The osteology of Alioramus, a gracile and long-snouted tyrannosaurid (Dinosauria: Theropoda) from the Late Cretaceous of Mongolia. Bulletin of the American Museum of Natural History 366: 1-197.

Brusatte, S.L., G.T. Lloyd, S.C. Wang, and M.A. Norell. 2014. Gradual assembly of avian body plan culminated in rapid rates of evolution across dinosaur-bird transition. Current Biology 24: 2386-2392.

Brusatte, S.L., M.A. Norell, T.D. Carr, G.M. Erickson, J.R. Hutchinson, A.M. Balanoff, G.S. Bever, J.N. Choiniere, P.J. Makovicky, and X. Xu. 2010. Tyrannosaur paleobiology: new research on ancient exemplar organisms. Science 329: 1481-1485.

Carpenter, K.D., D. Russell, D. Baird, and R. Denton. 1997. Redescription of the holotype of *Dryptosaurus aquilunguis* (Dinosauria: Theropoda) from the Upper Cretaceous of New Jersey. Journal of Vertebrate Paleontology 17: 561–573.

Carr, T.D., and D.J. Varricchio. 2014. A new species of *Daspletosaurus* from the Upper Two Medicine Formation (Late Campanian, Cretaceous) of Montana and evidence for anagenesis in tyrannosaurine evolution. Journal of Vertebrate Paleontology: 103A.

Carr, T.D., and T.E. Williamson. 2010. *Bistahieversor sealeyi*, gen. et sp. nov., a new tyrannosauroid from New Mexico and the origin of deep snouts in Tyrannosauroidea. Journal of Vertebrate Paleontology 30: 1-16.

Carr, T.D., T.E. Williamson, B.B. Britt, and K. Stadtman. 2011. Evidence for high taxonomic and morphologic tyrannosauroid diversity in the Late Cretaceous (Late Campanian) of the American Southwest and a new short-skulled tyrannosaurid from the Kaiparowits Formation of Utah. Naturwissenschaften 98: 241-246.

Carr, T.D., T.E. Williamson, and D.R. Schwimmer. 2005. A new genus and species of tyrannosauroid from the Late Cretaceous (Middle Campanian) Demopolis Formation of Alabama. Journal of Vertebrate Paleontology 25: 119–143.

Currie, P.J. 2003. Cranial anatomy of tyrannosaurid dinosaurs from the late Cretaceous Alberta, Canada. Acta Palaeontologica Polonica 48: 191–226.

Felsenstein, J. 1981. Evolutionary trees from DNA sequences: a maximum likelihood approach. Journal of Molecular Evolution 17: 368-376.

Fiorillo, A.R., and R.S. Tykoski. 2014. A diminutive new tyrannosaur from the top of the world. PLoS ONE 9(3): e91287 (doi:10.1371/journal.pone.0091287)

Fowler, D.W., H.N. Woodward, E.A. Freedman, P.L. Larson, and J.R. Horner. 2011. Reanalysis of “*Raptorex kriegsteini*”: a juvenile tyrannosaurid dinosaur from Mongolia. PLoS ONE 6(6): e21376.

Gold, M.E.L., S.L. Brusatte, and M.A. Norell. 2013. The cranial pneumatic sinuses of the tyrannosaurid *Alioramus* (Dinosauria: Theropoda) and the evolution of cranial pneumaticity in theropod dinosaurs. American Museum Novitates 3790: 1-46.

Goloboff, P.A., J.A. Farris, and K.C. Nixon. 2008. TNT, a free program for phylogenetic analysis. Cladistics 24: 774-786.

Holtz, T.R. 2001. The phylogeny and taxonomy of the Tyrannosauridae. *In* D.H. Tanke and K. Carpenter (editors), Mesozoic Vertebrate Life: 64–83. Bloomington: Indiana University Press.

Hone, D.W.E., K. Wang, C. Sullivan, X.-J. Zhao, S. Chen, D. Li, S. Ji, Q. Ji, and X. Xu 2011.A new, large tyrannosaurine theropod from the Upper Cretaceous of China. Cretaceous Research 32: 495-503.

Hutt, S., D.W. Naish, D.M. Martill, M.J. Barker, and P. Newberry. 2001. A preliminary account of a new tyrannosauroid theropod from the Wessex Formation (Early Cretaceous) of southern England. Cretaceous Research 22: 227–242.

Ji, Q., S.-A. Ji, and L.-J. Zhang. 2009. First large tyrannosauroid theropod from the Early Cretaceous Jehol Biota in northeastern China. Geological Bulletin of China 28: 1369-1374.

Jukes, T.H., and Cantor, C.R. 1969. Evolution of protein molecules. In H.N. Munro (editor), Mammalian Protein Metabolism: 21-132. New York: Academic Press.

Kass, R.E., and Raftery, A.E. 1995. Bayes factor. Journal of the American Statistical Association 90: 773-795.

Kurzanov, S.M. 1976. [A new Late Cretaceous carnosaur from Nogon-Tsav, Mongolia]. Joint Soviet-Mongolian Paleontological Expedition Transactions 3: 93–104. [Russian with English summary].

Li, D., M.A. Norell, K. Gao, N.D. Smith, and P.J. Makovicky. 2010. A longirostrine tyrannosauroid from the Early Cretaceous of China. Proceedings of the Royal Society Series B, 277: 183-190.

Loewen, M.A., R.B. irmis, J.J.W. Sertich, P.J. Currie, and S.D. Sampson. 2013. Tyrant dinosaur evolution tracks the rise and fall of Late Cretaceous oceans. PLoS ONE 8(11): e79420 (doi:10.1371/journal.pone.0079420)

Lü, J., L. Yi, S.L. Brusatte, L. Yang, H. Li, and L. Chen. 2014. A new clade of Asian Late Cretaceous long-snouted tyrannosaurids. Nature Communications 5: 3788 (DOI: 10.1038/ncomms4788)

Mader, B.J., and R.L. Bradley. 1989. A redescription and revised diagnosis of the syntypes of the Mongolia tyrannosaur *Alectrosaurus olseni*. Journal of Vertebrate Paleontology 9: 41-55.

Naish, D., S. Hutt, and D. M. Martill. 2001. Saurischian dinosaurs 2: Theropods; pp. 242–309 in D.M. Martill and D. Naish (editors), Dinosaurs of the Isle of Wight: 242-309. London: The Palaeontological Association.

Nesbitt, S.J. 2011. The early evolution of archosaurs: relationships and the origin of major clades. Bulletin of the American Museum of Natural History 352: 1-292.

Novas, F.E., F.L. Agnolín, M.D. Ezcurra, J. Porfiri, and J.I. Canale. 2013. Evolution of the carnivorous dinosaurs during the Cretaceous: the evidence from Patagonia. Cretaceous Research 45: 174-215.

Perle, A. 1977. [On the first finding of *Alectrosaurus* (Tyrannosauridae, Theropoda) in the Late Cretaceous of Mongolia]. Problemy Geologii Mongolii 3: 104–113. [Russian with English summary].

Rauhut, O.W.M. 2003. A tyrannosauroid dinosaur from the Late Jurassic of Portugal. Palaeontology 46: 903-910.

Rauhut, O.W.M., A.C. Milner, and S. Moore-Fay. 2010. Cranial osteology and phylogenetic position of the theropod dinosaur *Proceratosaurus bradleyi* (Woodward, 1910) from the Middle Jurassic of England. Zoological Journal of the Linnean Society 158: 155-195.

Ronquist, F., Teslenko, M., van der Mark, P., Ayres, D., Darling, A., Höhna, S., Larget, B., Liu, L., Suchard, M.A, and Huelsenbeck, J.P. 2012. MrBayes 3.2: efficient Bayesian phylogenetic inference and model choice across a large model space. Systematic Biology 61: 539-542.

Senter, P. 2007. A new look at the phylogeny of Coelurosauria (Dinosauria: Theropoda). Journal of Systematic Palaeontology 5: 429-463.

Sereno, P.C., L. Tan, S.L. Brusatte, H.J. Kriegstein, X. Zhao, and K. Cloward. 2009. Tyrannosaurid skeletal design first evolved at small body size. Science 326: 418-422.

Xu, X., J.M. Clark, C.A. Forster, M.A. Norell, G.M. Erickson, D.A. Eberth, C. Jia, and Q. Zhao. 2006. A basal tyrannosauroid dinosaur from the Late Jurassic of China. Nature 439: 715–718.

Xu, X., M.A. Norell, X. Kuang, X. Wang, Q. Zhao, and C. Jia. 2004. Basal tyrannosauroids from China and evidence for protofeathers in tyrannosauroids. Nature 431: 680–684.

Xu, X., K. Wang, K. Zhang, Q. Ma, L. Xing, C. Sullivan, D. Hu, S. Cheng, and S. Wang. 2012. A gigantic feathered dinosaur from the Lower Cretaceous of China. Nature 484: 92-95.
